# Response of peanut *Arachis hypogaea* roots to the presence of beneficial and pathogenic fungi by transcriptome analysis

**DOI:** 10.1038/s41598-017-01029-3

**Published:** 2017-04-19

**Authors:** Kun Hao, Feng Wang, Xiangqun Nong, Mark Richard McNeill, Shaofang Liu, Guangjun Wang, Guangchun Cao, Zehua Zhang

**Affiliations:** 1 State Key Laboratory for Biology of Plant Diseases and Insect Pests, Institute of Plant Protection, Chinese Academy of Agricultural Sciences, 100193 Beijing, China; 2 AgResearch, Lincoln Science Centre, Private Bag 4749, Canterbury, 8140 New Zealand

## Abstract

Entomopathogenic fungus *Metarhizium anisopliae* obtain survival benefit meanwhile promote the nutrient absorption of root as an endophyte. However, little is known concerning molecular mechanisms in the process. We performed the transcriptome sequencing of *A*. *hypogaea* roots inoculated *M*. *anisopliae* and pathogenic *Fusarium axysporum*, respectively. There were 81323 unigenes from 132023 transcripts. Total 203 differentially expressed genes (DEGs) respond to the two fungi, including specific 76 and 34 DEGs distributed respectively in *M*. *anisopliae* and *F*. *axysporum* treatment. KEGG pathway enrichment for DEGs showed the two top2 were signal transductions of plant-pathogen interaction and plant hormone. By qRT-PCR, the mRNA level of 26 genes involved in plant-fungus interaction confirmed the reliability of the RNA-Seq data. The expression pattern of the key DEGs on jasmonic acid (JA) or salicylic acid (SA) signaling pathway presented regulating consistency with JA or SA concentration detected by HPLC-MS. Those significantly stronger down-regulated DEGs by *M*. *anisopliae* thanby *F*. *axysporum* linking to hypersensitive response and negative regulation of defense, and those specific up-regulated genes in *M*. *anisopliae* treatment may predict that the less immunity is conducive to symbiosis *F*. *axysporum* may trigger JA-mediated defense regulated by ERF branch of JA signaling pathway, whereas M. an*isopliae* does not.

## Introduction

Peanut *Arachis Hypogaea* is a globally important crop for food and oil extraction. It is often attacked by a range of insect species and diseases causing serious yield losses in cultivation. It is widely grown in the semi-arid tropics, wherein China contributes the highest share and India ranks second by 41.6% and 12.5% in world production, respectively. The soil-dwell insect such as root-gnawing white grubs *Holotrichia* spp. and root rot pathogen *Fusarium oxysporum* Schlecht. could critically damaged the root system and impact nut production^1–3^. Pesticides and biopesticides were required repeat applications for controlling the pests and diseases to protect yields. The entomopathogenic fungus *Metarhizium anisopliae* (Metchn.) Sorokin is widely used as a biocontrol agent to reduce crop damage by pests, and has shown high potential efficiency in control of soil-dwelling pests *e*.*g*. beet root maggot *Tetanops myopaeformis*, wireworms *Agriotes* spp and white grubs *Holotrichia parallela* ^4–6^. While having a recognised role in insect control, *M*. *anisopliae* became of increasing interest because of its beneficial role on plant growth. It has been added to a list of fungi as a plant growth-promoter by rhizosphere competence and plant endophytes^7^. When *M*. *anisopliae* was applied to a cabbage experimental field at a rate of 10^13^ spores per ha, the fungal density could remain at 10^5^ propagules/g in the inner rhizosphere, while the amount was only of 10^3^ propagules/g in nonrhizosphere soil after several months^8^. Tomato plants treated with *M*. *anisopliae* had significantly greater plant height, root length, and shoot and root dry weight than those of the untreated control, although the response depended on isolate and inoculation rate^9^. Growth promotion following inoculation with *M*. *anisopliae* was also found for switchgrass (*Panicum virgatum*), haricot beans (*Phaseolus vulgaris*), corn (*Zea mays*) and peanut (*Arachis*. *hypogaea*)^10–12^. Furthermore, isotope labeling has shown that *M*. *anisopliae* has an important role in supplying nutrients to the plant through the transfer of nitrogen and the uptake of phosphorous and other minerals^13, 14^.

However, there is a paucity of information concerning the plant cell molecular responses during the initial colonization phase by *M*. *anisopliae* as both an insect pathogen and endophyte. Although research on invasive processes and mechanisms for pathogenicity of plant pathogenic fungi is extensive, it is assumed that in the field, plants must have different coping strategies to deal with beneficial compared to pathogenic fungi. Our previous research has used *M*. *anisopliae* to control white grubs *Holotrichia parallela* (Coleoptera: Scarabaeidae) and *H*. *oblita* (Coleoptera: Scarabaeidae) in peanut fields and determine persistence and proliferation of *M*. *anisopliae* applied to the peanut rhizosphere^6, 15^. In this study, the aim was to identify differences in molecular response in root tissue following the addition of either *M*. *anisopliae* or *F*. *axysporum*, to determine if beneficial versus pathogenic fungi produce corresponding or dissimilar cellular responses. To this end, we performed *de novo* transcriptome analysis of *A*. *hypogaea* roots following inoculation. Genes that were differentially expressed as the result of fungal induction were categorized and there mode of action determined.

## Results

### Sequencing and de novo transcriptome assembly

To investigate transcriptome expression in *A*. *hypogaea* root tissue treated with either *Metarhizium anisopliae* (AM), or *Fusarium axysporum* (AF), RNA was extracted from roots and sequenced using Illumina paired-end sequencing technology. A total of 180259860 Illumina PE raw reads were generated (Table 1). After removing adaptor sequences, ambiguous nucleotides and low-quality sequences, there were approximate 176 million clean reads remaining. Assembly of clean reads resulted in 132023 transcripts, including 81323 unigenes. They ranged 201–14967 bp with an average length of 786 bp and a N50 length of 1403 bp (Supplementary Fig. 1).

**Table 1:** Summary of *de novo* sequence assembly for *A*. *hypogaea* root treated with either *M*. *anisopliae* (AM), *F*. *axysporum* (AF) or untreated control (AC).

Sample	Raw Reads	Clean reads	Clean bases	Error (%)	Q20 (%)	Q30 (%)	GC (%)
AM_1	28172543	27615027	3.45G	0.03	96.67	93.25	44.37
AM_2	28172543	27615027	3.45G	0.03	95.59	91.65	44.38
AF_1	31723882	30884393	3.86G	0.03	96.81	93.52	44.32
AF_2	31723882	30884393	3.86G	0.03	95.52	91.53	44.31
AC_1	30233505	29523591	3.69G	0.03	96.76	93.45	43.96
AC_2	30233505	29523591	3.69G	0.03	95.71	91.88	43.96

### Annotation of all non-redundant unigenes

After eliminating repeated and short-length sequences, 81323 non-redundant unigenes were screened for similarity in seven public databases (Nr, Nt, Swiss-Prot, KEGG, GO, COG, Pfam) searching. The annotation results showed that 31399 unigenes (38.61%) had significant matches in the Nr database, 23129 (28.44%) in the Nt database. It was found that a smaller percentage 27.27% (22180 unigenes) was obtained when searching against the SWISS-PROT protein database rather than against the Nr database. In total, there were 36412 unigenes (44.77%) successfully annotated in at least one of the seven database, with 5235 unigenes (6.43%) in all seven databases (Table 2).

**Table 2:** Blast analysis of non-redundant unigenes against public databases.

Reference databases	Number of unigenes	Percentage (%)
Annotated in NR	31399	38.61
Annotated in NT	23129	28.44
Annotated in KO	9841	12.1
Annotated in SwissProt	22180	27.27
Annotated in PFAM	22953	28.22
Annotated in GO	23299	28.64
Annotated in KOG	10811	13.29
Annotated in all Databases	5235	6.43
Annotated in at least one Database	36412	44.77
Total Unigenes	81323	100

### Functional classification by GO, COG and KEGG

**Figure 1:**
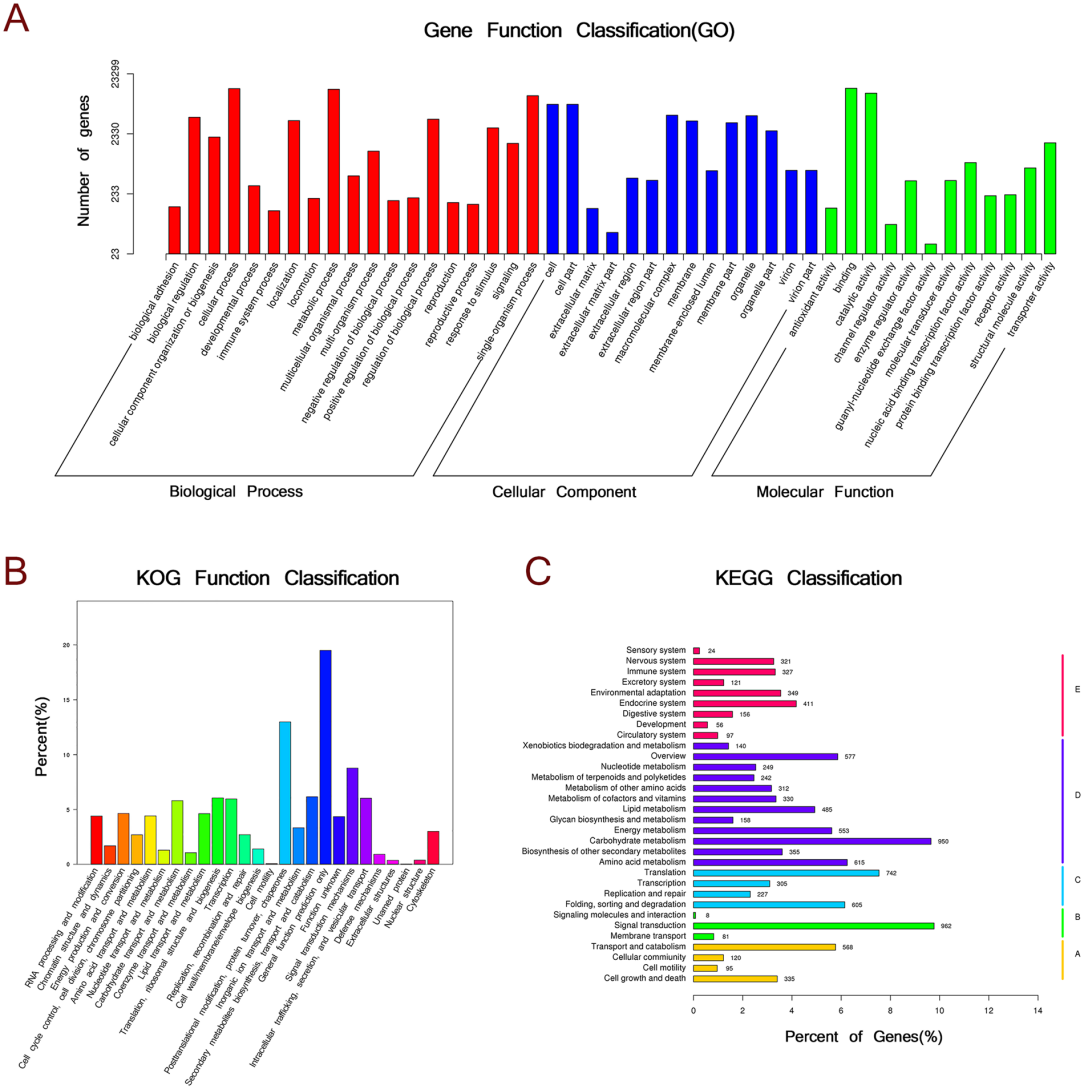
Functional categorization of unigenes specific to *A*. *hypogaea* following exposure to either *M*. *anisopliae* or *F*. *axysporum*, as determined from GO, COG and KEGG biological processes classification.

Gene Ontology (GO), an internationally standardized gene functional classification system, was used to classify the function of the predicted *A*. *hypogaea* unigenes. In total, 23299 unigenes with BLAST matches to known proteins were classified into three major functional ontologies using 1534 functional terms (Fig. 1a, Supplementary Table 1). As shown in Fig. 1a, the majority of the unigenes were assigned to the categories of biological processes (283091, 66.55%), followed by cellular components (80630, 18.95%) and molecular functions (61687, 14.50%). Under the category of biological processes, cellular processes (13205, 4.66%) and metabolic processes (12883, 4.55%) had the highest representation. Within cellular components, cell (7232, 8.97%) and cell part (7231, 8.97%) were highly represented, while for molecular functions, binding (13386, 21.70%) and catalytic activities (11077, 17.96%) were most represented (Supplementary Table 1). However, within each of the three major categories, a small number of genes were assigned to subcategories such as extracellular matrix part, channel regulator activity and guanyl-nucleotide exchange factor activity.

In order to predict and classify possible functions, all unigenes were aligned to the Cluster of Orthologous Groups (COG) database in which orthologous gene products were classified. 10811 non-redundant unigenes were subdivided into 26 COG classifications (Fig. 1b, Table 2). The cluster related to general function prediction only (2108, 19.50%) was the largest group, followed by those for post-translational modification, protein turnover, chaperon (1404, 12.99%) and signal transduction mechanisms (948, 8.77%).

The Kyoto Encyclopaedia of Genes and Genomes (KEGG) pathway database is a knowledge base for the systematic analysis of gene functions in terms of networks of genes and molecules in cells. Based on KEGG analysis, 9841 unigenes were assigned to 267 pathways (Table 2, Supplementary Table 2). The pathways involving the largest number of unique transcripts were signal transduction (962, 6.50%), which may be involved in fungi-plant interactions, followed by carbohydrate metabolism (950, 6.42%), whereas signaling molecules and interaction (8) was the smallest group (Fig. 1c, Supplementary Table 2).

### Global differential expression genes

To reveal the molecular mechanism for plant and fungi interactions, the differential expression genes (DEGs) induced by inoculation with *M*. *anisopliae* and *F*. *axysporum* were analyzed. The resulting Pearson’s correlation coefficient (R^2^) between samples were quite high in AM vs AC (R^2^ = 0.843), AF vs AC (R^2^ = 0.843) and AM vs AF (R^2^ = 0.861) (Supplementary Fig. 2). The expression level of each assembled transcript sequence in different samples was measured through RPKM (Reads per kilo-base per million reads) values, and DEGs (q-value < 0.005 and log2 (fold change) > 1) were defined as genes that were significantly enriched or depleted in one sample relative to the other.

**Figure 2:**
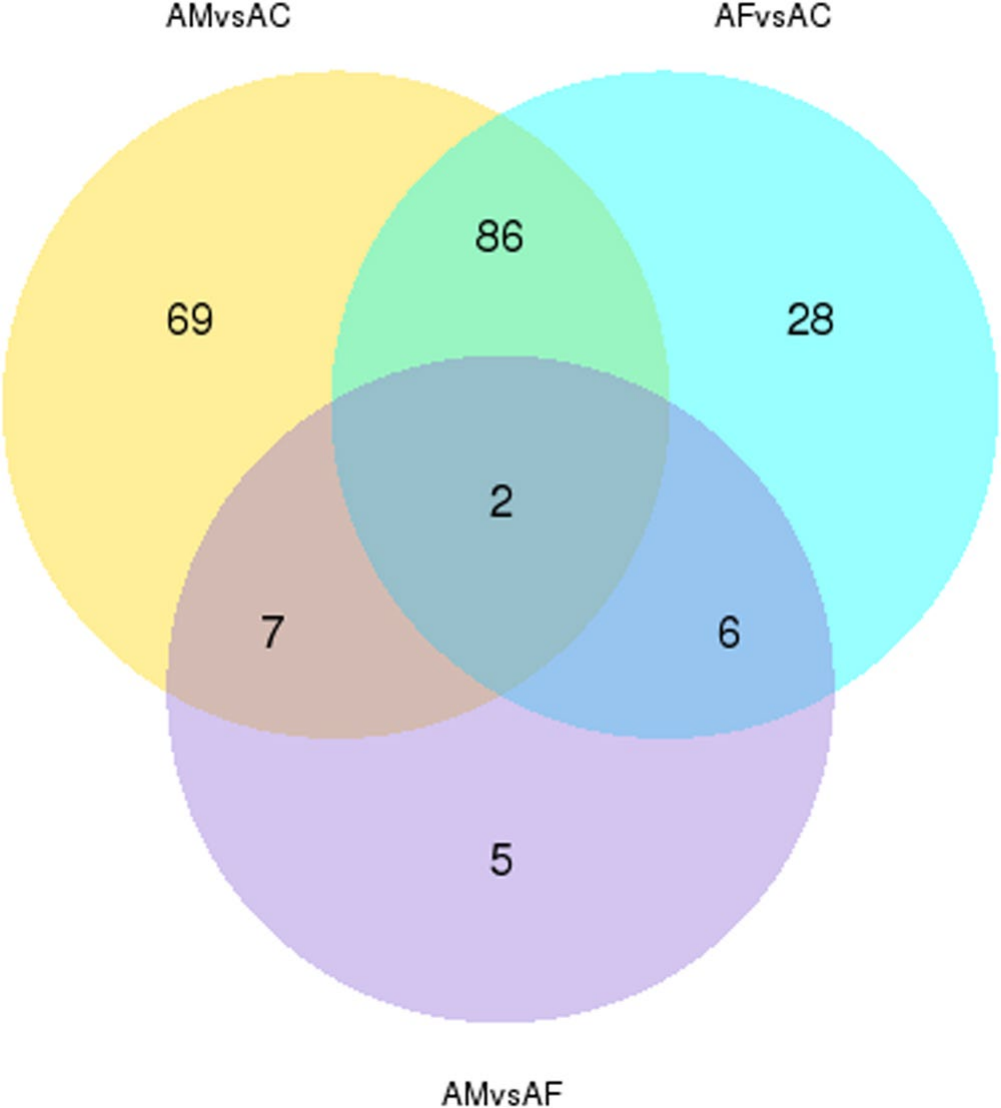
Venn diagram visualizing the number of differential expressed genes for treatments of *M*. *anisopliae* (AM), *F*. *axysporum* (AF) or control (AC). (The numbers of shared DEGs are indicated in the overlapping parts of the circles).

Based on the criteria above, of the 81323 unigenes, 164, 122 and 20 were detected as significantly different in the comparison between AM vs AC, AF vs AC and AM vs AF, respectively. Among these DEGs, 16 were up- and 148 down-regulated, respectively in AM vs AC. For AF vs AC, 28 were up- and 94 down-regulated, respectively. For AM vs AF, 13 up- and 7 down-regulated, respectively (Supplementary Fig. 3). As shown in the Venn diagram, a total of 203 DEGs were shared by AM, AF and AC. Individual inoculation with pathogenic fungus *F*. *axysporum* specifically regulated 34 genes. 76 DEGs with special expression pattern in AM vs AC probably functioned combinative symbiotic process (Fig. 2).

### Plant-fungus interaction genes

From whole non-redundant unigenes, 1468 resistant protein (RP) genes were identified, including 258 TIR-NB-LRR, 25 CC-NB-LRR, 296 LRR receptor protein kinase and 21 LysM receptor protein kinase (Table 3). There were 14 symbiosis-related proteins identified, including SymRK, CASTOR, POLLUX and CYCLOPS (Table 3). According to KEGG annotation, all of them relate to plant-fungus interactions, involved in plant hormone synthesis, pathogen-resistance and other symbiotic related pathways. On the plant hormone signal transduction pathway (ko04075), 311 unigenes were annotated to 41 proteins. On the plant-pathogen interaction pathway (ko04626), 223 unigenes were annotated to 28 proteins. Additionally, 473 unigenes were annotated to 107 proteins, which are associated with eight pathways for synthesizing plant hormones. These pathways were tryptophan metabolism (ko00380), zeatin biosynthesis (ko00908), diterpenoid biosynthesis (ko00904), carotenoid biosynthesis (ko00906), cysteine and methionine metabolism (ko00270), brassinosteroid biosynthesis (ko00905), alpha-Linolenic acid metabolism (ko00592) and phenylalanine metabolism (ko00360) (supplementary Table 3).

**Table 3:** Genes involve in plant-fungus interaction.

Gene Description	Unigene number
TIR-NB-LRR	258
CC-NB-LRR	25
LRR receptor protein kinase	296
LysM receptor protein kinase	21
other disease resistant protein	868
MAPK cascade	71
WRKY transcription factor	116
jasmonate ZIM domain-containing protein	8
coronatine-insensitive protein 1	2
ethylene-responsive transcription factor	136
Transcription factor MYC	6
transcription factor TGA	23
nonexpressor of pathogenesis-related protein	21
proteasome related protein	122
ubiquitin related protein	279
defensin	30
bark storage protein	3
pathogenesis-related protein	9
1-aminocyclopropane-1-carboxylate oxidase and synthase	49
metacaspase	22
respiratory burst oxidase homolog protein	16
cyclic nucleotide-gated ion channel	41
G-protein coupled receptor related	176
calcium-dependent protein kinase	31
Calmodulin	200
Calcineurin	58
phospholipase C	33
symbiosis related protein (SymRK, CASTOR, POLLUX, CYCLOPS)	14

### Functional distribution and connection of differentially expressed genes

**Figure 3:**
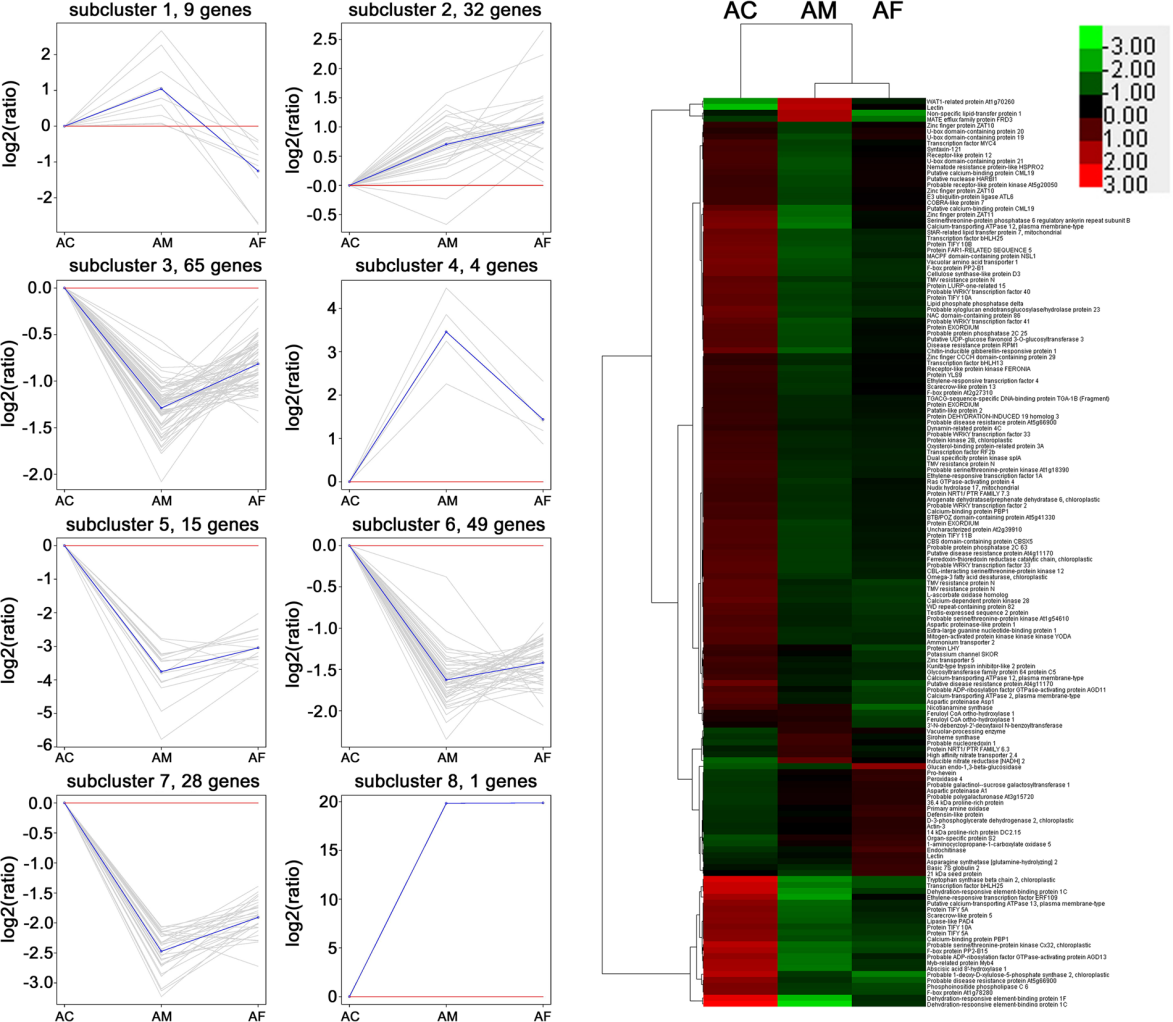
Cluster analysis of DEGs identified by Transcriptome comparisons of *M*. *anisopliae* treatment (AM), *F*. *axysporum* treatment (AF) and control (AC). Right: Heatmap of DEGs across the three treatments. Red indicates high expression and green indicates low expression. Chroma color from red to green indicates log10 (FPKM + 1) from more to less. Left: Expression patterns of the genes in the eight main clusters corresponding to the heatmap. Gray lines represent relative expression of a gene cluster under different experimental conditions, the blue line represents the average of the relative expression of all the genes in the cluster. Y-axis shows the Log2 (ratios) relative expression levels.

All the DEGs in inoculating treatment with *M*. *anisopliae* (AM) and *F*. *axysporum* (AF), were hierarchically clustered into eight sub-clusters based on k-means and identified. For the two fungal treatments compared to the control (AC), the genes with similar down-regulation pattern were assigned to subcluster 3, 5, 6 and 7, and the genes showing similar up-regulation were assigned to subcluster 2, 4 and 8. The genes in subcluster 2 showed significantly higher up-regulation in AF compared to AM, while the genes in subcluster 4 showed significantly higher up-regulation in AM compared to AF. In particular, the genes in subcluster 1 were up-regulated in AM but down-regulated in AF (Fig. 3).

The functional characteristics of the DEGs up-regulated by *M*. *anisopliae* induction were related to following three aspects, (i) oxidation-reduction process (Feruloyl CoA ortho-hydroxylase 1, Inducible nitrate reductase [NADH] 2, Probable nucleoredoxin 1), (ii) transport process (MATE efflux family protein FRD3, lipid transfer protein-like protein, WAT1-related protein At1g70260, high affinity nitrate transporter 2.4, protein NRT1/PTR FAMILY 6.3) and (iii) metabolic process (Siroheme synthase, vacuolar processing enzyme). On the other hand, the genes drastically up-regulated by *F*. *axysporumin* induction in subcluster 2 were related to following five aspects, (i) defense (class I chitinase, pro-hevein, defensin D2, lectin, peroxidase, primary amine oxidase, sieve element occlusion a, actin-3), (ii) oxidation-reduction process (1-aminocyclopropane-1-carboxylate oxidase), (iii) transport process (lipid transfer protein), (iv) metabolic process (glucan endo-1,3-beta -glucosidase-like, galactinol-sucrose galactosyltransferase 1-like, phosphoglycerate dehydrogenase, probable polygalacturonase At3g15720, asparagine synthetase), and (v) proteolysis (eukaryotic aspartyl protease family protein, aspartic proteinase, Ulp1 protease family carboxy-terminal domain protein) (Supplementary Table 4). It is significant that only nine DEGs were up-regulated in the *M*. *anisopliae* treatment but exhibited down-regulation in the *F*. *axysporum* treatment. This portion of the gene is highly likely to be associated with a symbiotic relationship between *M*. *anisopliae* and *A*. *hypogaea*. Five genes were annotated as, 3′-N-debenzoyl-2′-deoxytaxol N-benzoyltransferase, MATE efflux family protein FRD3, Non-specific lipid-transfer protein 1 and two Feruloyl CoA ortho-hydroxylase 1 (Supplementary Table 4). The remaining four genes were unannotated in the current database.

Further, the functional distribution of the up- and down-regulated DEGs were compared in different treatments. Based on GO annotation, only 16 genes in total DEGs were significantly enriched on the two GO stems (Corrected p-value < 0.05) (Table 4). They were all appeared in the *M*. *anisopliae* treatment, with nucleic acid binding transcription factor (GO:0001071) and sequence-specific DNA binding transcription factor (GO:0003700) activity. The directed acyclic graph by topGO showed that sequence-specific DNA binding transcription factor activity (GO:0003700) was subject to nucleic acid binding transcription factor activity (GO:0001071), and then both were classified into the main stem of molecular function (GO:0003674). The 16 DEGs of peanut response to *M*. *anisopliae* included a variety of transcription factor, such as WRKY, MYC, TGA, ethylene-responsive transcription factors, dehydration-responsive element-binding proteins and nitrate transporters (Table 4).

**Table 4:** The differentially expressed transcription factors in AM vs AC enriched on two GO terms.

Gene_id	Length	Swissprot Description	Description
c44598_g1	1070	Probable WRKY transcription factor 2	sequence-specific DNA binding transcription factor activity//nucleic acid binding transcription factor activity
c49605_g1	2199	Protein NRT1/PTR FAMILY 6.3	sequence-specific DNA binding transcription factor activity//nucleic acid binding transcription factor activity
c38925_g1	1570	Dehydration-responsive element-binding protein 1C	sequence-specific DNA binding transcription factor activity//nucleic acid binding transcription factor activity
c40371_g1	1259	Ethylene-responsive transcription factor 4	sequence-specific DNA binding transcription factor activity//nucleic acid binding transcription factor activity
c43589_g1	1156	Ethylene-responsive transcription factor ERF109	sequence-specific DNA binding transcription factor activity//nucleic acid binding transcription factor activity
c44588_g1	1344	Dehydration-responsive element-binding protein 1C	sequence-specific DNA binding transcription factor activity//nucleic acid binding transcription factor activity
c46800_g1	3505	TGACG-sequence-specific DNA-binding protein TGA-1B	sequence-specific DNA binding transcription factor activity//nucleic acid binding transcription factor activity
c47117_g1	1426	—	sequence-specific DNA binding transcription factor activity//nucleic acid binding transcription factor activity
c48161_g1	1219	Dehydration-responsive element-binding protein 1F	sequence-specific DNA binding transcription factor activity//nucleic acid binding transcription factor activity
c48617_g1	3787	Probable WRKY transcription factor 41	sequence-specific DNA binding transcription factor activity//nucleic acid binding transcription factor activity
c49002_g1	3889	Transcription factor RF2b	sequence-specific DNA binding transcription factor activity//nucleic acid binding transcription factor activity
c50433_g1	1549	Probable WRKY transcription factor 33	sequence-specific DNA binding transcription factor activity//nucleic acid binding transcription factor activity
c50672_g1	2840	Probable WRKY transcription factor 33	sequence-specific DNA binding transcription factor activity//nucleic acid binding transcription factor activity
c55830_g1	1714	Ethylene-responsive transcription factor 1A	sequence-specific DNA binding transcription factor activity//nucleic acid binding transcription factor activity
c68816_g1	2082	Transcription factor MYC4	sequence-specific DNA binding transcription factor activity//nucleic acid binding transcription factor activity
c68944_g1	972	Zinc finger protein ZAT11	sequence-specific DNA binding transcription factor activity//nucleic acid binding transcription factor activity

Biological behaviors of DEGs are complicated, being related to many inner-cell metabolic pathways. KEGG pathway enrichment analysis for DEGs revealed the combinative pathways that multidirectional connecting to defense-linked regulation. As showed in Table 5, the interesting results were that signal transductions of plant-pathogen interaction and plant hormone were the top two enriched pathways for the down-regulated DEGs in both AF vs AC and AM vs AC (Corrected p-value < 0.05). They went through MYC or ERF branch on the jasmonic acid (JA) signaling pathway and defense-linked RPM1, CDPK and WRKY points. Individually, glycine, serine and threonine metabolism and phenylalanine metabolism were distinctly enriched for the up-regulate DEGs in AF vs AC (Corrected p-value < 0.05), while nitrogen metabolism, lysosome degradation and antigen processing and presentation pathways were distinctly enriched for the up-regulate DEGs in AM vs AC (Corrected p-value < 0.05). Particularly, nitrogen metabolism, amino sugar and nucleotide sugar metabolism were enriched in AM vs AF DEGs (Corrected p-value < 0.05). The regulating change converged from multi-directions was able to correct some metabolic pathways.

**Table 5:** KEGG pathway enrichment analysis for DEGs.

Term	ID	Corrected **p-value**	Input Unigene ID
AF vs AC down			
Plant-pathogen interaction	ko04626	0.00003808	c50433_g1|c51871_g1|c10189_g1|c20251_g1|c48568_g1|c48568_g2
AF vs AC up			
Glycine, serine and threonine metabolism	ko00260	0.03511172	c50764_g1|c52036_g1
Phenylalanine metabolism	ko00360	0.05348501	c50764_g1|c45822_g1
AM vs AC down			
Plant-pathogen interaction	ko04626	0.00000000	c31769_g1|c50433_g1|c47627_g2|c50672_g1|c51871_g1|c68816_g1|c10189_g1|c20251_g1|c39301_g1|c44598_g1|c48568_g1|c48568_g2
Plant hormone signal transduction	ko04075	0.04087051	c68816_g1|c39301_g1|c48568_g1|c10189_g1|c48568_g2
AM vs AC up			
Nitrogen metabolism	ko00910	0.00007551	c47696_g1|c52585_g1
Antigen processing and presentation	ko04612	0.02083884	c49130_g1
Lysosome	ko04142	0.02083884	c49130_g1
AM vs AF down			
Amino sugar and nucleotide sugar metabolism	ko00520	0.00929927	c22869_g1
AM vs AF up			
Nitrogen metabolism	ko00910	0.00202396	c52585_g1

In spite of the intricate metabolic pathways, a limited signal crossing or gene interacting still could be found and speculated. KEGG pathway showed that phenylalanine metabolic pathway was linked to salicylic acid (SA) biosynthesis. Also, abscisic acid (ABA) biosynthesis was showed linking to carotenoid biosynethesis pathway. L-phenylalanine was known as a substrate for the synthesis of SA, besides that, it was an influence factor on carotenoid aggregation. Although nitrogen metabolic pathways were enriched in both AM vs AC and AF vs AC, nitrogen-transporting protein appeared significantly high, along with nitrate reductase in AM vs AC. In contrast, some immune-related metabolic pathways such as lysosome degradation and antigen processing and presentation were enriched in AF vs AC (Table 5).

### Validation of differentially expressed genes using qRT-PCR

**Figure 4:**
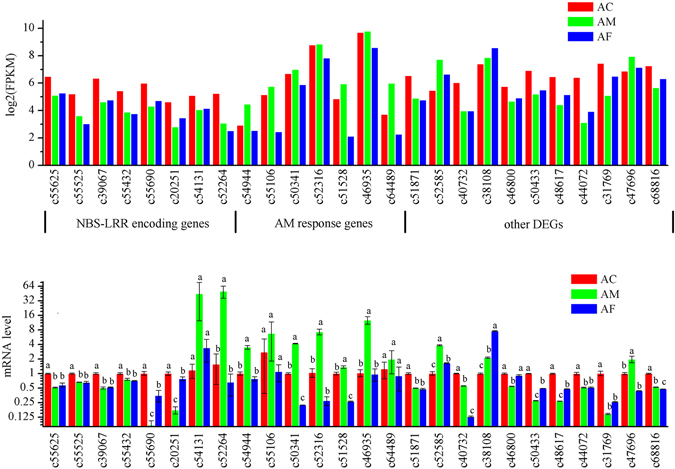
Quantitative real-time PCR (qRT-PCR) validations of 26 differentially expressed genes in AC (red), AM (green) and AF (blue). For each qRT-PCR validation, three technical replications were performed, beta-tubulin gene was used as internal control. All results were expressed as means ± standard error (SE) of the number of experiments. The lowercase ‘a’, ‘b’ and ‘c’ indicated that statistically significant difference of mRNA level was considered on an error probability of p < 0.05 by one-way ANOVA using Duncan’s multiple-range test.

The RNA level of 26 DEGs, including the genes of nine NBS-LRRs (c55625, c55525, c39067, c55432, c55690, c20251, c54131, c52264), a hypothetical transcription factor with zinc knuckle (c54994), a retrotransposon (c55106), a 3′-N-debenzoyl-2′-deoxytaxol N-benzoyltransferase (c50341), two feruloyl CoA ortho-hydroxylase 1 (c52316, c46935), a lipid transfer protein-like protein (c64489), a hypothetical transcript (c51528), a calcium-dependent protein kinase (c51871), a nitrate reductase (c52585), a pathogenesis-related protein (c40732), a plant defensin-like protein (c38108), a TGACG-sequence-specific DNA-binding protein (c46800), a WRKY transcription factor 33 (c50433), a WRKY transcription factor 53 (c48617), a MYB transcription factor (c44072), a calcium-binding protein (c31769), a high affinity nitrate transporter (c47696) and a MYC transcription factor (c68816), were examined by qRT-PCR to confirm the reliability of the RNA-Seq data (Fig. 4). The result showed that most of the unigene expression patterns were consistent with the RNA-seq data, though the fold change of qRT-PCR and DEG analysis was not exactly matched. It indicated that our RNA-Seq data were valid.

### Detection of salicylic acid (SA) and jasmonic acid (JA) in roots of peanuts under different treatments

**Figure 5:**
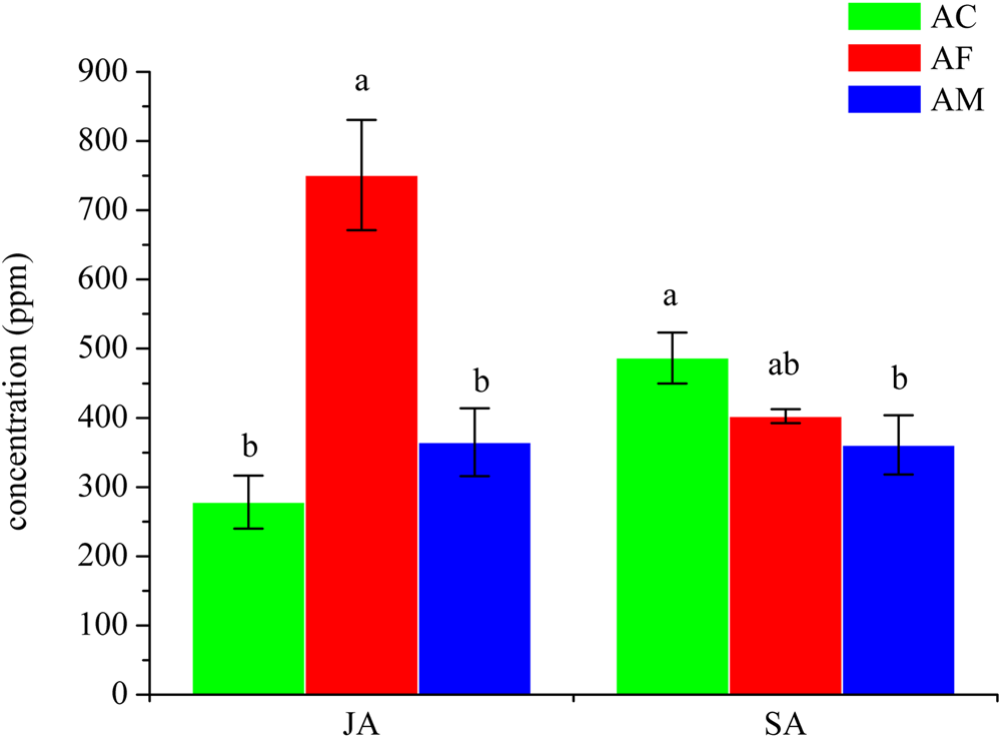
SA and JA concentrations in AC (green), AF (red) and AM (blue). All results were expressed as means ± standard error (SE) of the number of experiments. The lowercase ‘a’, ‘b’ and ‘ab’ indicated that statistically significant difference of concentration was considered on an error probability of p < 0.05 by one-way ANOVA using Duncan’s multiple-range test.

HPLC-MS was used to detect the concentration of JA and SA in roots of peanuts, which were separately treated by distilled water (AC), *F*. *axysporum* (AF) and *M*. *anisopliae* (AM) (Fig. 5). The results showed that there was no significant difference between AM and AC in the concentration of JA, which was around 320 ppm, while the concentration of JA in AF was significantly higher than that in AC or AM, which getting 750 ppm, The concentration of SA seemed similar among AC, AF and AM, which ranged 361–486 ppm. They were not significantly different between AC and AF and between AF and AM. However, the concentration of SA in AM was significantly lower than that in AC. The statistically significant difference of hormone concentration was considered on an error probability of p < 0.05 by one-way ANOVA (SPSS version 16.0, SPSS) using Duncan’s multiple-range test.

Comparing to the DEGs in JA and SA signaling pathways that verified by qRT-PCR as above, we found that the concentration of JA was highest in AF but relatively low in AC and AM, which consistent with the highest expression level of defensin (c38108) by qRT-PCR as well the highest mRNA level of defensin by transcriptome analysis in AC. It is known that defensin is a marker gene in ERF branch of JA signaling pathway. Another coincidence is that mRNA level of MYC transcript factor, a key gene on the JA signaling pathway, is also lower in AC and AM. On the other hand, in SA signaling pathway, mRNA level of those DEGs including transcription factors WRKY (c50433, c48617), MYB (c44072) and TGA (c46800) and pathogenesis-related protein (c40732) are lower in AF and AM than in AC, that consistent with the SA concentration in the above detection. The results provided one more support for RNA-Seq data reliability.

## Discussion

Based on the regulated direction, function distribution and KEGG pathway enrichment of the DEGs in AM vs AC and AF vs AC, a comprehensive integration may resolve the early different response to beneficial *M*. *anisopliae* and pathogenic *F*. *axysporum* in peanut roots, and provide clues to mechanisms underpinning specific symbiosis by *M*. *anisopliae*.

### Similar response of peanut root to inoculation of beneficial and pathogenic fungi

Of the 203 differentially expressed genes (DEGs) identified in AM vs AC and AF vs AC comparisons, 192 (c.94.6%), appeared in the same direction of up- or down-regulation (Fig. 3). It suggested that there were similar pattern in plant cellular response and interaction with pre-invading fungi, whether they are beneficial or pathogenic to plant. The pattern may resemble or equivalent to the first tier of plant immune system. Plant evolved pattern recognition receptors (PRRs) can be activated by recognition of evolutionarily conserved pathogen-associated molecular patterns (PAMPs) which then trigger the mitogen-activated protein kinase (MAPK) cascades. In turn, this activates plant hormone signaling to integrate various aspects of the multi-layered plant defense response (called pattern-triggered immunity or PTI)^16, 17^. The optional signaling may guide different types of immune reaction^18^. Furthermore, it is also acknowledged that fungi can produce effectors to suppress the basal PTI by suppressing immune signaling, inhibiting MAPK cascade or blocking resistant protein expression^19, 20^. These co-evoluted effectors are deployed to modify host cell processes or to associate with the host so that reaching biotrophic lifestyle for part, if not all, of their lifecycle^21^. In turn, plant disease resistance proteins (R proteins) could recognize pathogen effectors to induce effector-triggered immunity (ETI) which usually cause hypersensitive response (HR)^22, 23^. Colonization of the roots by either *M*. *anisopliae* or *F*. *oxysporum* may mean that they are recognized by the same PRRs located on the surface of root cells, which consequently leads to similar cellular responses as found in this study.

In the transcriptome of this experiment, the part of genes up-regulated may be activated to identify and defend against invading pathogens. We found the common up-regulated genes from subclusters 2, 4 and 8 in Fig. 3, linking to several biological processes including chitinase, pro-hevein, peroxidase, proteinase, nitrate reductase and lectin. These proteins and enzymes are directly or indirectly involved in the regulation of plant defense. For example, vacuolar processing enzyme (VPE), (c49130_g1) which was up-regulated in AM vs AC and AF vs AC, may be a cysteine protease responsible for caspase -1 activity and promote programmed cell death (PCD) by disrupting the vacuole in pathogenesis like in tobacco^24^. It may positively regulate HR cell death mediated by NB-LRR recognition of the invading pathogen like metacaspase-1 in a variety of plants^25^. The up-regulated 1-amino-cyclopropane-1-carboxylate oxidase (ACO) may be involved in ethylene synthesis, reasoning from 1-amino-cyclopropane-1-carboxylic acid (ACC) is ethylene precursor^26^. Lectins are a group of sugar-binding proteins which could regulate immune response to guide plant defense^27^. Likewise, chitinases often contain a hevein domain which related to defense by carbohydrate degradation^28, 29^. Besides, one actin-like pathogenesis related protein and one defensin also found in up-regulated DEGs in AM vs AC and AF vs AC.

Another part of genes, down-regulated in both AM vs AC and AF vs AC, may be suppressed by the two fungi to facilitate subsequent invasion. We found commonly recorded down-regulated genes from subcluster 3, 5, 6 and 7 in Fig. 3, that referred to a variety of physiological processes, such as plant-pathogen interaction, plant hormone signal transduction, amino acids metabolism, starch and sucrose metabolism. The similar response to both fungi may be required to directly deal with fungal invasion or to trigger subsequent immune reaction in peanut cells. For example, we identified nine TIR-NB-LRR or CC-NB-LRR resistance proteins, as well as one LRR receptor-like serine/threonine-protein kinase, one chitin elicitor receptor kinase and three receptor-like protein kinase. They may play important role in recognizing specific effectors. In regards to SA, a plant hormone closely related to plant disease resistance, we found a down-regulated heat shock protein HSP70 that was thought to process a defense-promoting function by promoting SA pathway^30^. Some enzymes related to SA synthesis such as one EDS1/PAD4, one tryptophan synthase and one arogenate dehydratase (choloroplastic-like), and some transcription factors increasing SA accumulate by positive regulation of ICS gene encoded in isochorismate synthase pathway, such as MYBs, WRKYs and WIPK, also were down-regulated. On the jasmonic acid signaling pathway, we found two pathogenesis-related marker genes PDF1.2 and VSP2, their down-regulation would be for MYC and ERF branches for JA signaling suppression^17^. The MYC branch and the ERF branch are two major branches of the JA signaling pathway^31^. There were four down-regulated MYC and ERF transcription factors in AM vs AC and AF vs AC, they may be repressed by jasmonate ZIM-domain (JAZ) transcriptional repressors. The COI1-JAZ co-receptor complex leads to ubiquitination and proteasome-dependent degradation of JAZ repressors and release of MYC or ERF transcription factors from transcriptional repression^32–34^. Ubiquitination regulates pattern-recognition receptor signaling that mediates immune responses^35^. There are four E3 ubiquitin protein ligase down-regulated in AMvsAC and AFvsAC. On the other hand, we found down-regulated (+)-abscisic acid 8′-hydroxylase involved in the ABA catabolic pathway. ABA promotes MYC branch by protein phosphatase 2C (PP2C) and antagonizes ERF branch^31, 36^. In addition, A chitin-inducible gibberellin-responsive protein was found down-regulated. It could regulate the growth-repessing DELLA proteins which acts positively on JA signaling by sequestering JAZ repressor^37, 38^. DELLA proteins maybe play an important role in hormonal cross talk to repress defense responses in symbiosis^39^.

As described above, down-regulated DEGs may be suppressed to weaken immune and inhibit development. These multi-pathway process may be very complex, but they do provide us with some important joints resulting transcriptomic and metabolomic data subsets.

### Specific response of peanut roots to the presence of beneficial fungus compared to pathogenic fungus

The genes, up-regulated expression in *M*. *anisopliae* treatment but down-regulated expression in *F*. *axysporum* treatment, should be particularly concerned (Fig. 3, subcluster 1). Ferric Redictase Defective 3 (FRD3), a MATE family member, has been shown to be an efflux transporter of the efficient iron chelator citrate in *A*. *thaliana*. It mediates-citrate release in the apoplastic space, and represents an important process by which efficient iron nutrition is achieved between adjacent tissues lacking symplastic connections, maintaining iron homeostasis throughout plant development^40, 41^. Iron was identified as an essential micronutrient for the legume–rhizobium symbiosis^42^. Another special DEG c50341_g1 up-regulate in AM vs AC but down-regulate in AF vs AC, has acetyltransferase activity and a NB-ARC domain which makes us surmise it as a receptor to symbiotic effectors. And more, some lipid transfer proteins should be required for the successful symbiotic association between a microbe and its host, for example, lipid transfer MtN5 for the symbiosis of *Sinorhizobium meliloti* and *Medicago truncatula* ^43^. The role of high affinity nitrate transporter which was significantly up-regulated in AM vs AC may be closely contacted with the evidence of *M*. *anisopliae* providing more nitrogen sources for the plant^6, 44^. Mutual benefits were an important basis for symbiosis. The other two enzymes, Feruloyl CoA ortho-hydroxylase 1 and 3′-N-debenzoyl-2′-deoxy taxol N-benzoyltransferase, have not been reported as having a role in plant-fungal symbiosis. But there is a possibility that they have an important role on synthesis of scopoletin and taxol, respectively^45, 46^. Scopoletin has been shown to have distinctly antifungal synergistic effects in *Melia azedarach* L.^47^. Taxol, usually extracted from plants and used as antitumor, was identified antimitotic activity produced by endophytic fungi^48^.

Furthermore, in the above 3.1 description, those DEGs in the same direction of up- or down-regulation presented different regulatory levels in *M*. *anisopliae* treatment and in *F*. *axysporum* treatment. After a detailed analysis on their functions and metabolic pathways, we were surprised to find that almost all of significantly stronger down-regulated DEGs in AM than in AF were linked to those transcription factors involved in hypersensitive response (HR) and in negatively regulating defense (Supplemental Table 1). HR represents a strong incompatibility and a rapid response to prevent the spread of microbial infection by PCD in plant. In this experiment, the HR factors CaM, CDPK, PBS, RPM1 and RPS were down-regulated, which may serve to weaken the strength of the defense response in the plants when interacting with the fungi. This phenomenon was also found in other pathogenic fungi infecting to plants. Barley powdery mildew *Blumeria graminis* and the leaf spot disease *Stemphylium lycopersici* can suppress HR-based PCD^49–51^. *Phytophthora infestans* and *Mycosphaerella pinodes* can suppress HR-based defense when they infect host plants^52, 53^. We also found that all of those down-regulated resistance proteins and related kinase in AM vs AC and AF vs AC, including nine TIR/CC-NB-LRR, one LRR receptor-like serine/threonine-protein kinase, one chitin elicitor receptor kinase and three receptor-like protein kinase, were expressed significantly lower in AM vs AC than in AF vs AC. Integrated analysis of the transcription factors down-regulated found that most of them involved in negatively regulating defense by their respective paths, including JA-JAZ-ERF/MYC, FLS-MEKK-WRKY and GA-GID2-DELLA, or by cross regulation of JA path with SA, ET or ABA. Conversely, almost all of significantly stronger up-regulated DEGs in AF than in AM were assigned to enzymes and proteins involved in positively regulating defense. They include PR, peroxidase, chitinase, lectin and indole glucosinolate (Supplemental Table 1). Therefore, these results suggest that, even though both the two fungi encountered a plant resistance defense, the path and strength of the response were different. For *M*. *anisopliae*, the defense was weaker and came from indirect multipath of hormone regulation. The indirect multipath regulation may be accompanied by other responses as description above. For *F*. *axysporum*, the defense was stronger and more direct. It may be because *F*. *axysporum* triggered JA-mediated defenses against necrotrophic fungal pathogens, which regulated by ERF branch of JA signaling pathway, whereas the non-pathogenic fungus *M*. *anisopliae* did not^54^.

As it was well known, plant defense implemented by a refined immune system in which various hormones interact forming complex network. In spite of only 4 d interaction between peanut and the two fungi in the experiment, the DEGs distribution can still expose some clues of hormones cross-regulation. The transcriptional factors MYC and ERF were branched to two major lines of the JA signaling pathway^31^. The MYC controlled the branch including the downstream marker gene vegetative storage protein 2 (VSP2), while the ERF as an ethylene response factor regulated the other branch including the marker gene plant defensing (PDF)^55, 56^. The ERF branch of the JA pathway is associated with enhanced resistance to necrotrophic pathogens. SA activate PRs (pathogen related genes) by the TGA transcription factor^57, 58^. Heat shock protein 70 (HSP70) is thought to possess a defense-promoting function by promoting the SA pathway^30^. There is also cross talk between SA and JA pathway. The growth-repressing DELLA proteins are regulated by gibberellins which acts positively on JA signaling by sequestering JAZ repressor^37, 38^. Ethylene acts synergistically on the expression of ERF branch of JA pathway, whereas it antagonizes the MYC branch^59^. However, ABA promotes MYC branch by PP2C and antagonizes ERF branch^31, 36^. WRKY transcription factors are essential for plant defense response^60^. LRR receptor-like serine/threonine-protein kinase FLS2 activate WRKY25 and WRKY33 to repress defense-related gene induction by MAPK cascades signal transduction^61^. Recently, cross regulation of plant hormone was reported more interpretation^31^. In this study, it can be inferred that cross regulation of peanut hormones guided the down-regulated expression of numerous transcription factors in both *M*. *anisopliae* and *F*. *axysporum* treatments. Moreover, those transcription factors, significantly stronger down-regulated in AM than in AF, directly or indirectly resulted in changing activity of the enzymes and proteins related to defense by negative regulation. Oppositely, the enzymes and proteins related to defense such as PDF (plant defensin), PRs (pathogen related proteins), peroxidase, chitinase and lectin, etc. were significantly higher in AF than in AM. This indicates that in the early stages of infection, both beneficial *M*. *anisopliae* and pathogenic *F*. *axysporum* suppressed some important transcription factors by various hormones pathway and inspired plant resistance. The difference is that *F*. *axysporum* caused stronger immunity than *M*. *anisopliae* done. The predictable result is that the less immunity in *M*. *anisopliae* treatment could be conducive to establish a symbiotic relationship.

### Supposing the pathway factors of *M*. *anisopliae* symbiosis in plant

Beneficial interaction could help *M*. *anisopliae* colonization and symbiosis in plant. Based on the results and analysis of this study, we speculate that *M*. *anisopliae* symbiosis in plant might be divided into two parts. One part might contain weakening HR and regulating defense response, nutrient supply and some specific induction, which related to those significantly stronger down-regulated transcription factors in AM than in AF. Another part might be similar to known common symbiotic way via a central pathway consisted of symbiosis receptor-like protein kinase (SymRK), Ion channel (CASTOR/POLLUT) and calcium- and calmodulin- dependent protein kinase (CCaMK), as if azotobacter in legume and arbuscular mycorrhizal fungi in various plant. The latter part had not yet been induced and differently expressed in the early interactions of plant and fungi in this experiment. Another possibility is that *M*. *anisopliae* may have a different pathway to establish successful symbiosis.

## Materials and Methods

### Sample collection and preparation of peanut *Arachis hypogaea* L. roots

To investigate root response to pathogen invasion of beneficial and harmful fungi we established three treatments: *A*. *hypogaea* inoculated with *M*. *anisopliae* (AM), inoculated with *F*. *axysporum* (AF) and an un-inoculated control (AC). The treatments were established in aseptic condition to minimize cross contamination from other microorganisms associated with the *A*. *hypogaea* rhizosphere, including harmful and beneficial to roots. Furthermore, it is difficult to determine the progression of infection by infection time because *M*. *anisopliae* colonizes the rhizosphere for an indeterminate period before becoming endophytic and enter the root system. The testing peanut *A*. *hypogaea* species was the Chinese cultivar Luhua-11. The soaking seeds were sown in pots containing sterilized vermiculite, one seed in one pot. The plants grow in a controlled climate chamber at 25 °C and L:D 14:10. After seven days, when seedlings were at the two lateral branches stage, 20 mL conidial suspensions of *M*. *anisopliae* (1 × 10^7^ spore/mL) or of *F*. *axysporum* (1 × 10^7^ spore/mL) were carefully drip irrigated to each root, respectively. The control plants received 100 mL of sterile water. Four days following treatment, the samples were collected by carefully pouring pots, sweeping aside vermiculite, removing the plants and washing the roots in sterile water. The roots were cut and immediately immersed in liquid nitrogen, then stored at −70 °C. To increase the accuracy of detection, three subsamples of peanut roots were bulked into one sample for each RNA seq sample.

### RNA extraction, library construction and sequencing

Total RNA was isolated from frozen medullar tissue by using the RNA plant mini kit with column DNase digestion (Qiagen, Hilden, Germany) following the manufacturer’s instructions. RNA degradation and contamination was detected on 1% agarose gels. RNA concentration was then measured using Qubit RNA Assay Kit in Qubit 2.0 Flurometer (Life Technologies, Carlsbad, CA, USA). Additionally, RNA integrity was assessed using the RNA Nano 6000 Assay Kit of the Bioanalyzer 2100 system (Agilent Technologies, Santa Clara, CA, USA).

A total of 3 μ g RNA per sample was used as input material for the RNA preparations. Finally, three samples with RNA integrity number (RIN) values above 8 were used for construction of the libraries. Sequencing libraries were generated using NEBNext Ultra™ RNA Library Prep Kit for Illumina (NEB, USA) following manufacturer’s recommendations and index codes were added to attribute sequences to each sample. Briefly, mRNA was purified from total RNA using poly-T oligo-attached magnetic beads. Fragmentation was carried out using divalent cations under elevated temperature in NEBNext First Strand Synthesis Reaction Buffer. First strand cDNA was synthesized using random hexamer primer and M-MuLV Reverse Transcriptase (RNaseH^−^). Subsequently, second strand cDNA synthesis was performed using DNA Polymerase I and RNase H. Remaining overhangs were converted into blunt ends via exonuclease/polymerase activities. After adenylation of 3′ ends of DNA fragments, NEBNext Adaptor with hairpin loop structure was ligated to prepare for hybridization. In order to select cDNA fragments of preferential 150~200 bp in length, the library fragments were purified with AMPure XP system (Beckman Coulter, Beverly, USA). Then 3 μl USER Enzyme (NEB, USA) was used with size-selected, adaptor-ligated cDNA at 37 °C for 15 min followed by 5 min at 95 °C. This was followed by PCR performed using Phusion High-Fidelity DNA polymerase, Universal PCR primers and Index (X) Primers, respectively. Finally, PCR products were purified (AMPure XP system) and library quality was assessed on the Agilent Bioanalyzer 2100 system.

The clustering of the index-coded samples was performed on a cBot Cluster Generation System using TruSeq PE Cluster Kit v3-cBot-HS (Illumia) according to the manufacturer’s instructions. After cluster generation, the library preparations were sequenced on an Illumina Hiseq 2500 platform and 125 bp paired-end reads were generated.

### Sequence reads mapping, assembly and annotation

Raw data (raw reads) of Fastq format were firstly processed through in-house Perl scripts. In this step, clean data (clean reads) were obtained by removing reads containing adapter, reads containing ploy-N and low quality reads from raw data. At the same time, Q20, Q30, GC-content and the sequence duplication level of the clean data were calculated. All the downstream analyses were based on high quality clean data.

The left files (read1 files) from all samples were pooled into one big left.fq file, and right files (read2 files) into one big right.fq file. Transcriptome assembly was accomplished based on the left.fq and right.fq using Trinity with min_kmer_cov set to a default value of 2 and all other parameters set to default^62^.

Gene function was annotated based on the following seven databases: Nr (NCBI non-redundant Protein sequences), Nt (NCBI non-redundant nucleotide sequences), Pfam (Protein family), KOG/COG (Clusters of Orthologous Groups of proteins), Swiss-Prot (a manually annotated and reviewed protein sequence database), KO (KEGG Ortholog database) and GO (Gene Ontology). Data for each sequenced library was analysed using BLAST with a cutoff E-value of 10^−5^.

### Differential expression analysis

Prior to differential gene expression analysis, for each sequenced library, the read counts were adjusted by edge R program package through one scaling normalized factor. Differential expression analysis of two samples was performed using the DEGseq (2010) R package. The p-value was adjusted using q-value^63^. q-value < 0.005&|log2 (fold change)| > 1 was set as the threshold for significantly differential expression.

### GO and KEGG enrichment analysis of differentially expressed transcripts

Gene Ontology (GO) enrichment analysis of the differentially expressed genes (DEGs) was implemented by the GO seq R packages based on the Wallenius non-central hyper-geometric distribution, which can adjust for gene length bias in DEGs^64^.

KEGG is a database resource for understanding high-level functions and utilities of the biological system, such as the cell, the organism and the ecosystem, from molecular-level information, especially large-scale molecular datasets generated by genome sequencing and other high-throughput experimental technologies (http://www.genome.jp/kegg/)^65^. We used KOBAS software to test the statistical enrichment of differential expression genes in KEGG pathways^66^.

### Confirmation of the expression profiles by qRT-PCR

Differentially expressed genes identified by the above described method were validated using quantitative real-time PCR (qPCR). The real-time PCR was performed with the SYBR Premix ExTaqTM (TaKaRa, Dalian, China) on the ABI 7500 Real-Time PCR System (Applied Biosystems, Foster City, CA, USA). The beta-tubulin gene was used as a reference control. The reaction was performed using the following conditions: denaturation at 95 ration 60 s, followed by 40 cycles of amplification (95 ol. The15s, 60 60 llo 60 s). Each plate was repeated three times in independent runs for all reference and selected genes. Gene expression was evaluated by the 2^−ΔΔCt^ method^66^.

### Statistical analysis

For each sample, three technical replicates of the qRT-PCR assay were used. Results were expressed as means ± standard error (SE) of the number of experiments. The statistically significant difference of gene expression was considered only on an error probability of p < 0.05 by one-way ANOVA (SPSS version 16.0, SPSS) using Duncan’s multiple-range test.

### LC-MS conditions

Analysis was performed on a Thermo Scientific NCS-3500RS Ultimate 3000 HPLC system Binary Rapid system coupled to a Thermo Scientific QExactive Mass spectrometer (Thermo Scientific, Fremont, CA). The TheUltimate 3000 system equipped with a pump (LPG 3X00), auto sampler (ACC-3000), column oven and diode array UV/VIS detector (DAD-3000(RS)). Chromatographic separation was executed on a ACQUITY UPLC HSS T3 C18 column (1.7 µm, 2.1 × 100 mm). SPE was performed with a negative pressure manifold, andevaporation under nitrogen with a TurboVap LV® evaporator fromZymark (Hopkinton, MA, USA).

Gradient elution was performed withmobile phase A (0.1% formic acid in water) and B (acetonitrile) at 0.4 mL/min flow rate and at 40 °C. The initial composition (10% B) was maintained for 1 min, increased from 10% to100% B for 10 min, and returned to initial conditions over 23 min. A 1 min equilibration followed, yielding a total run time of 35 min.

The Q Exactive mass spectrometer was equipped with heated electrospray ionization source (HESI-II) and operated in the positive ionization mode. We optimized the following parameters: spray voltage 3700 V, capillary temperature 320 °C, heater temperature 425 425sheath gas and auxiliary gas flow were optimized at 30 and 10 arbitrary units respectively.

### Preparation of LC-MS standard solutions and sample

Five concentrations (10 mg/L, 50 mg/L, 100 mg/L, 500 mg/L, 1000 mg/L) of SA and six concentrations (10 mg/L, 50 mg/L, 100 mg/L, 500 mg/L, 1000 mg/L, 5000 mg/L) of JA solutions were seperately used for the establishment of calibration curves. The stock solutions were stored at 4 °C. Calibration curves gave the respective equations y = 1699580*X, R² = 0.9956 and y = 672873*X, R² = 0.9965.

1 g of each peanut root samples were taken to grind the homogenate, and then add 1.5 mL 1:1 (v:v) mixture of ethanol and methanol to the homogenate. Ultrasonic for 2 hours followed by 13000 g centrifugation. The supernatant was stored at 4 °C for LC-MS detection. The sample solution was filtered through a 0.22 μm filter before LC-MS injection.

Results were expressed as means ± standard error (SE) of the number of experiments. The statistically significant difference of hormone concentration was considered only on an error probability of p < 0.05 by one-way ANOVA (SPSS version 16.0, SPSS) using Duncan’s multiple-range test.

## Electronic supplementary material

supplementary information

## References

[1] Anitha V, Wightman J, Rogers DJ (2005). Management of white grubs (Coleoptera: Scarabaeidae) on groundnut in southern India. Int J Pest Manage.

[2] Porter DM, Smith DH, Rodríguezkábana R (1984). Compendium of peanut diseases. Journal of Periodontology.

[3] Lynch, R. E. & Mack, T. P. Biological and biotechnical advances for insect management in peanut. *Advances in Peanut Science*. *Stillwater*, *OK*: *American Peanut Research and Education Society* 95–159 (1995).

[4] Jaronski ST, Fullerschaefer C, Larson B, Jacobsen BJ (2007). Effect of three bacterial disease-control agents on the entomopathogenic fungi, Metarhizium anisopliae and Beauveria bassiana. From the. Biennial Meeting.

[5] Guery, B. Metarhizium anisopliae, a means of fighting wireworms soil pests: results and outlook. In 5th Conférence Internationale sur les Méthodes Alternatives de Protection des Plantes, 11–13 mars, 2015, Nouceau Sièle, Lille, France 325–334 (2015).

[6] Liu X (2011). Biocontrol of Peanut White Grubs, Holotrichia parallela, Using Entomopathogenic Fungus *Metarhizium anisopliae* at Sowing Period of Peanut. Chinese Journal of Biological Control.

[7] Ownley BH, Gwinn KD, Vega FE (2010). Endophytic fungal entomopathogens with activity against plant pathogens: ecology and evolution. Biocontrol.

[8] Hu G, St LJ (2003). Field studies using a recombinant mycoinsecticide (Metarhizium anisopliae) reveal that it is rhizosphere competent. Applied & Environmental Microbiology.

[9] Elena GJ, Beatriz PJ, Alejandro P, Lecuona RE (2011). Metarhizium anisopliae (Metschnikoff) Sorokin promotes growth and has endophytic activity in tomato plants. Adv Biol Res.

[10] Sasan RK, Bidochka MJ (2012). The insect-pathogenic fungus Metarhizium robertsii (Clavicipitaceae) is also an endophyte that stimulates plant root development. Am J Bot.

[11] Kabaluk JT, Ericsson JD (2007). Seed Treatment Increases Yield of Field Corn When Applied for Wireworm Control. Agron J.

[12] Liu, S. F. *et al*. (Plant Protection Sciences, 2016).

[13] Behie SW, Zelisko PM, Bidochka MJ (2012). Endophytic insect-parasitic fungi translocate nitrogen directly from insects to plants. Science.

[14] Usuki F, Narisawa K (2007). A mutualistic symbiosis between a dark septate endophytic fungus, Heteroconium chaetospira, and a nonmycorrhizal plant, Chinese cabbage. Mycologia.

[15] Liu, X. *et al*. Persistence and proliferation of a Chinese s.s. isolate in the peanut plant root zone. *Biocontrol Science & Technology* **26**, 1–36 (2016).

[16] Cui H, Tsuda K, Parker JE (2015). Effector-Triggered Immunity: From Pathogen Perception to Robust Defense. Annu Rev Plant Biol.

[17] Pandey D, Gaur M, Sajeesh PK, Kumar A (2016). Plant Defense Signaling and Responses Against Necrotrophic Fungal Pathogens. J Plant Growth Regul.

[18] Akira S, Hemmi H (2003). Recognition of pathogen-associated molecular patterns by TLR family. Immunol Lett.

[19] Meng X, Zhang S (2013). MAPK cascades in plant disease resistance signaling. Annu Rev Phytopathol.

[20] Zhang Z (2012). Disruption of PAMP-Induced MAP Kinase Cascade by a Pseudomonas syringae Effector Activates Plant Immunity Mediated by the NB-LRR Protein SUMM2. Cell Host & Microbe.

[21] Fevre, R. L., Evangelisti, E., Rey, T. & Schornack, S. Modulation of host cell biology by plant pathogenic microbes. *Annual Review of Cell & Developmental Biology* **31**, 201–229 (2015). 10.1146/annurev-cellbio-102314-112502 26436707

[22] Jones JDG, Dangl JL (2006). The plant immune system. Nature.

[23] Liu Y (2007). Chloroplast-generated reactive oxygen species are involved in hypersensitive response-like cell death mediated by a mitogen-activated protein kinase cascade. Plant J.

[24] Kariya K (2013). A novel mechanism of aluminium-induced cell death involving vacuolar processing enzyme and vacuolar collapse in tobacco cell line BY-2. J Inorg Biochem.

[25] Coll NS, Epple P, Dangl JL (2011). Programmed cell death in the plant immune system. Cell Death & Differentiation.

[26] Kakei, Y. *et al*. Transcriptome analysis of hormone-induced gene expression in Brachypodium distachyon. *Scientific Reports* **5**, doi:10.1038/srep14476 (2015). 10.1038/srep14476 PMC4588574 26419335

[27] Sun YY, Liu L, Li J (2015). Three novel B-type mannose-specific lectins of Cynoglossus semilaevis possess varied antibacterial activities against Gram-negative and Gram-positive bacteria. Developmental & Comparative Immunology.

[28] Price NPJ, Momany FA, Schnupf U, Naumann TA (2015). Structure and disulfide bonding pattern of the hevein-like peptide domains from plant class IV chitinases ☆. Physiological & Molecular Plant Pathology.

[29] Asensio JL (2000). Structural basis for chitin recognition by defense proteins: GlcNAc residues are bound in a multivalent fashion by extended binding sites in hevein domains. Chem Biol.

[30] Jelenska J, van Hal JA, Greenberg JT (2010). Pseudomonas syringae hijacks plant stress chaperone machinery for virulence. P Natl Acad Sci USA.

[31] Pieterse CMJ, Does DVD, Zamioudis C, Leonreyes A, Wees SCMV (2012). Hormonal modulation of plant immunity. Annual Review of Cell & Developmental Biology.

[32] Thines B (2007). JAZ repressor proteins are targets of the SCFCOI1 complex during jasmonate signalling. Nature.

[33] Yan Y (2007). A downstream mediator in the growth repression limb of the jasmonate pathway. Plant Cell.

[34] Chini A (2007). The JAZ family of repressors is the missing link in jasmonate signalling. Nature.

[35] Hu H, Sun SC (2016). Ubiquitin signaling in immune responses. Cell Res.

[36] Fernando, V. D. & Schroeder, D. F. Role of ABA in Arabidopsis Salt, Drought, and Desiccation Tolerance. *Abiotic and Biotic Stress in Plants–Recent Advances and Future Perspectives*, 507–524 (2016).

[37] Sun TP (2011). The Molecular Mechanism and Evolution of the GA–GID1–DELLA Signaling Module in Plants. Current Biology Cb.

[38] Wild M (2012). The Arabidopsis DELLA RGA-LIKE3 is a direct target of MYC2 and modulates jasmonate signaling responses. Plant Cell.

[39] Limpens E, Zeijl AV, Geurts R (2015). Lipochitooligosaccharides modulate plant host immunity to enable endosymbioses. Annu Rev Phytopathol.

[40] Roschzttardtz H, Séguélaarnaud M, Briat JF, Vert G, Curie C (2011). The FRD3 citrate effluxer promotes iron nutrition between symplastically disconnected tissues throughout Arabidopsis development. Plant Cell.

[41] Vidhyasekaran, P. Salicylic Acid Signaling in Plant Innate Immunity. In *Plant Hormone Signaling Systems in Plant Innate Immunity* 27–122, doi:10.1007/978-94-017-9285-1 (2015).

[42] Brear EM, Day DA, Smith PMC (2013). Iron: an essential micronutrient for the legume-rhizobium symbiosis. Frontiers in Plant Science.

[43] Pii Y, Astegno A, Peroni E, Zaccardelli M, Pandolfini T, Crimi M (2009). The Medicago truncatula N5 gene encoding a root-specific lipid transfer protein is required for the symbiotic interaction with Sinorhizobium meliloti. Mol Plant Microbe In.

[44] Behie SW, Bidochka MJ (2014). Ubiquity of insect-derived nitrogen transfer to plants by endophytic insect-pathogenic fungi: an additional branch of the soil nitrogen cycle. Applied & Environmental Microbiology.

[45] Kosuke K (2008). Scopoletin is biosynthesized via ortho -hydroxylation of feruloyl CoA by a 2-oxoglutarate-dependent dioxygenase in Arabidopsis thaliana. Plant J.

[46] Walker K, Long R, Croteau R (2002). The final acylation step in taxol biosynthesis: cloning of the taxoid C13-side-chain N-benzoyltransferase from Taxus. Proceedings of the National Academy of Sciences.

[47] Carpinella MC, Ferrayoli CG, Palacios SM (2005). Antifungal Synergistic Effect of Scopoletin, a Hydroxycoumarin Isolated from Melia azedarach L. Fruits. Journal of Agricultural & Food Chemistry.

[48] Garyali S, Kumar A, Reddy MS (2014). Diversity and antimitotic activity of taxol-producing endophytic fungi isolated from Himalayan yew. Ann Microbiol.

[49] Piffanelli P (2002). The Barley MLO Modulator of Defense and Cell Death Is Responsive to Biotic and Abiotic Stress Stimuli. Plant Physiol.

[50] Hückelhoven R, Dechert C, Kogel KH (2003). Overexpression of barley BAX inhibitor 1 induces breakdown of mlo-mediated penetration resistance to Blumeria graminis. Proceedings of the National Academy of Sciences.

[51] Bouarab K, Melton R, Peart J, Baulcombe D, Osbourn A (2002). A saponin-detoxifying enzyme mediates suppression of plant defences. Nature.

[52] Doke, N. Prevention of the hypersensitive reaction of potato cells to infection with an incompatible race of Phytophthora infestans by constituents of the zoospores. Prevention of the hypersensitive reaction of potato cells to infection with an incompatible race of Phytophthora infestans by constituents of the zoospores. *Physiological Plant Pathology* **7**, 1–4, N1, 5–7, doi:10.1016/0048-4059(75)90053-3 (1975).

[53] Yoshioka H, Shiraishi T, Yamada T, Ichinose Y, Oku H (1990). Suppression of Pisatin Production and ATPase Activity in Pea Plasma Membranes by Orthovanadate, Verapamil and a Suppressor from Mycosphaerella pinodes. Plant & Cell Physiology.

[54] Antico C, Colon C, Banks T, Ramonell K (2012). Insights into the role of jasmonic acid-mediated defenses against necrotrophic and biotrophic fungal pathogens. Frontiers in Biology.

[55] Lorenzo O, Chico JM, Sánchezserrano JJ, Solano R (2004). JASMONATE-INSENSITIVE1 encodes a MYC transcription factor essential to discriminate between different jasmonate-regulated defense responses in Arabidopsis. Plant Cell.

[56] Pre M, Atallah M, Champion AVM, Pieterse C, Memelink J (2008). The AP2/ERF domain transcription factor ORA59 integrates jasmonic acid and ethylene signals in plant defense. Plant Physiol.

[57] Fan W, Dong X (2002). *In vivo* interaction between NPR1 and transcription factor TGA2 leads to salicylic acid-mediated gene activation in Arabidopsis. Plant Cell.

[58] Després C, Delong C, Glaze S, Liu E, Fobert PR (2000). The Arabidopsis NPR1/NIM1 protein enhances the DNA binding activity of a subgroup of the TGA family of bZIP transcription factors. Plant Cell.

[59] Pieterse CEMJ, Leon-Reyes A, Ent SVD, Wees SCMV (2009). Networking by small-molecule hormones in plant immunity. Nature Chemical Biology.

[60] Eulgem T, Somssich IE (2007). Networks of WRKY transcription factors in defense signaling. Curr Opin Plant Biol.

[61] Ichimura K (2002). Mitogen-activated protein kinase cascades in plants: a new nomenclature. Trends Plant Sci.

[62] Grabherr MG (2013). Trinity: reconstructing a full-length transcriptome without a genome from RNA-Seq data. Nat Biotechnol..

[63] Storey, J. D. The False Discovery Rate: A Bayesian Interpretation and the q-value. *Annals of Statistics* **31**, 2013–2035 (2003).

[64] Young MD, Wakefield MJ, Smyth GK, Oshlack A (2010). Gene ontology analysis for RNA-seq: accounting for selection bias. Genome Biology.

[65] Kanehisa M (2008). KEGG for linking genomes to life and the environment. Nucleic Acids Res.

[66] Mao X, Cai T, Olyarchuk JG, Wei L (2005). Automated genome annotation and pathway identification using the KEGG Orthology (KO) as a controlled vocabulary. Bioinformatics.

