# Supplementary material for: Response of peanut Arachis hypogaea roots to the presence of beneficial and pathogenic fungi by transcriptome analysis
Source: Sci Rep. 2017 Apr 19;7:964. doi: 10.1038/s41598-017-01029-3 (PMC5430461; doi:10.1038/s41598-017-01029-3)
Supplement: Supplementary file 1 — supplementary information [file 41598_2017_1029_MOESM1_ESM.doc]

Supplementary information of manuscript entitled of response of peanut *Arachis hypogaea* roots to the presence of beneficial and pathogenic fungi by transcriptome analysis by Kun Hao, Feng Wang, Xiangqun Nong, Mark Richard McNeill, Shaofang Liu, Guangjun Wang, Guangchun Cao, Zehua Zhang

Supplementary fig. 1 Transcriptome assemble information


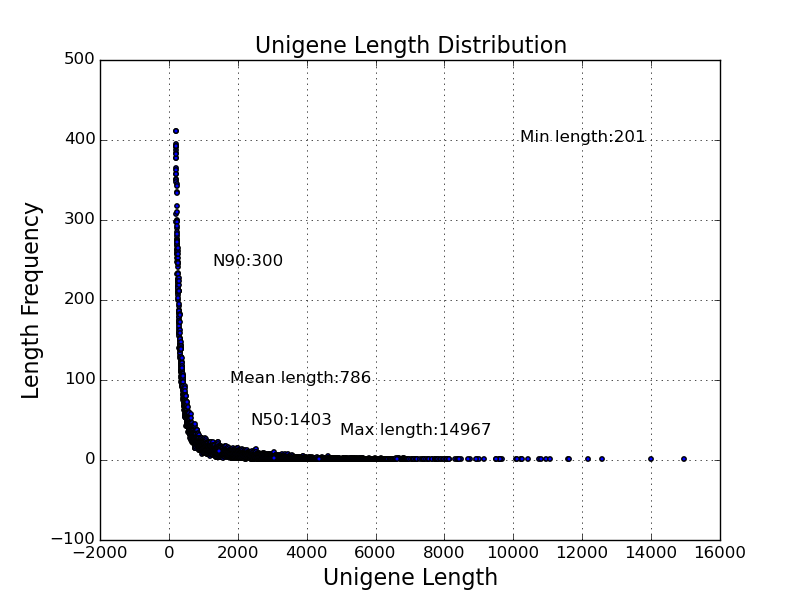


Supplementary fig. 2 Correlation between samples


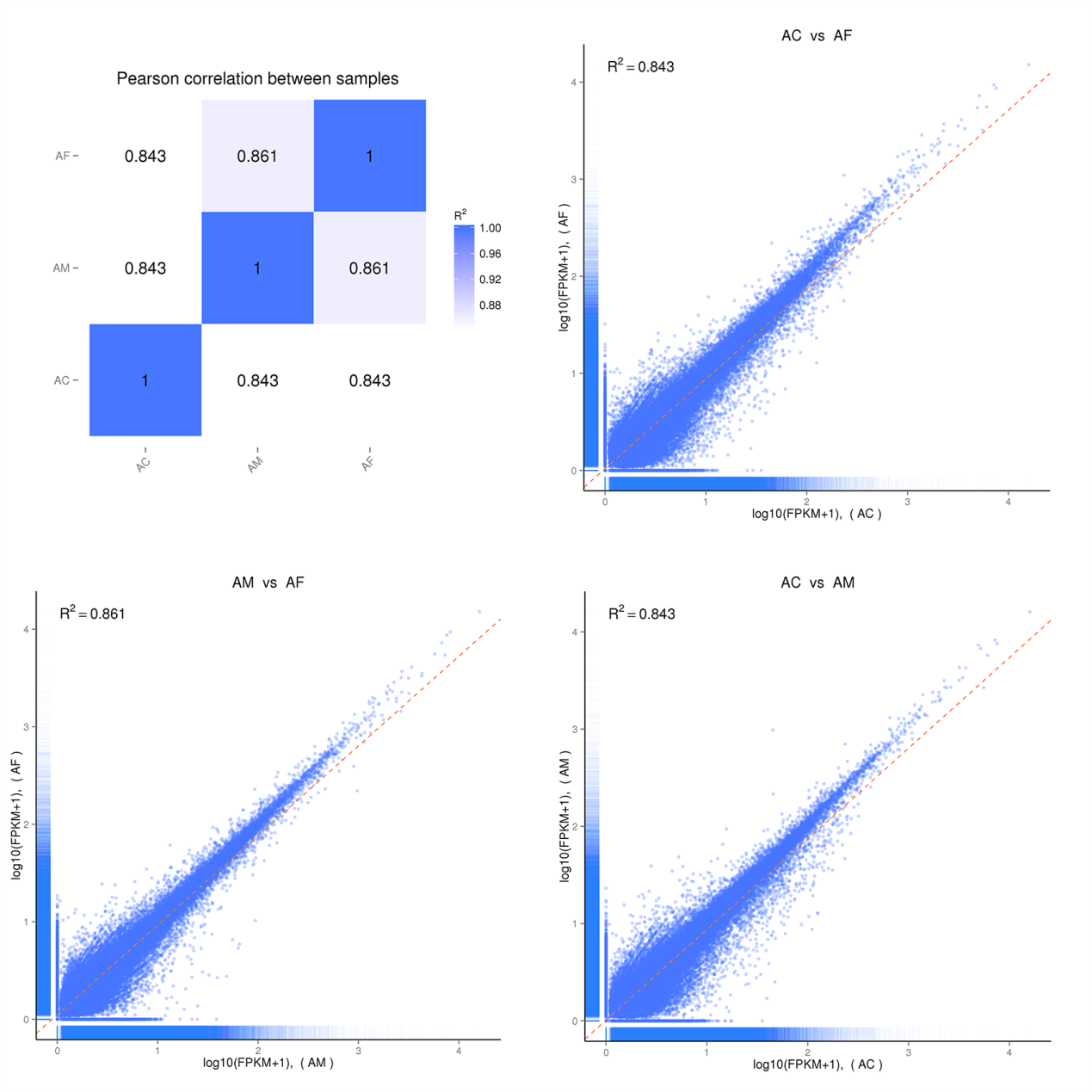


Supplementary fig. 3 Volcanoplot of DEGs


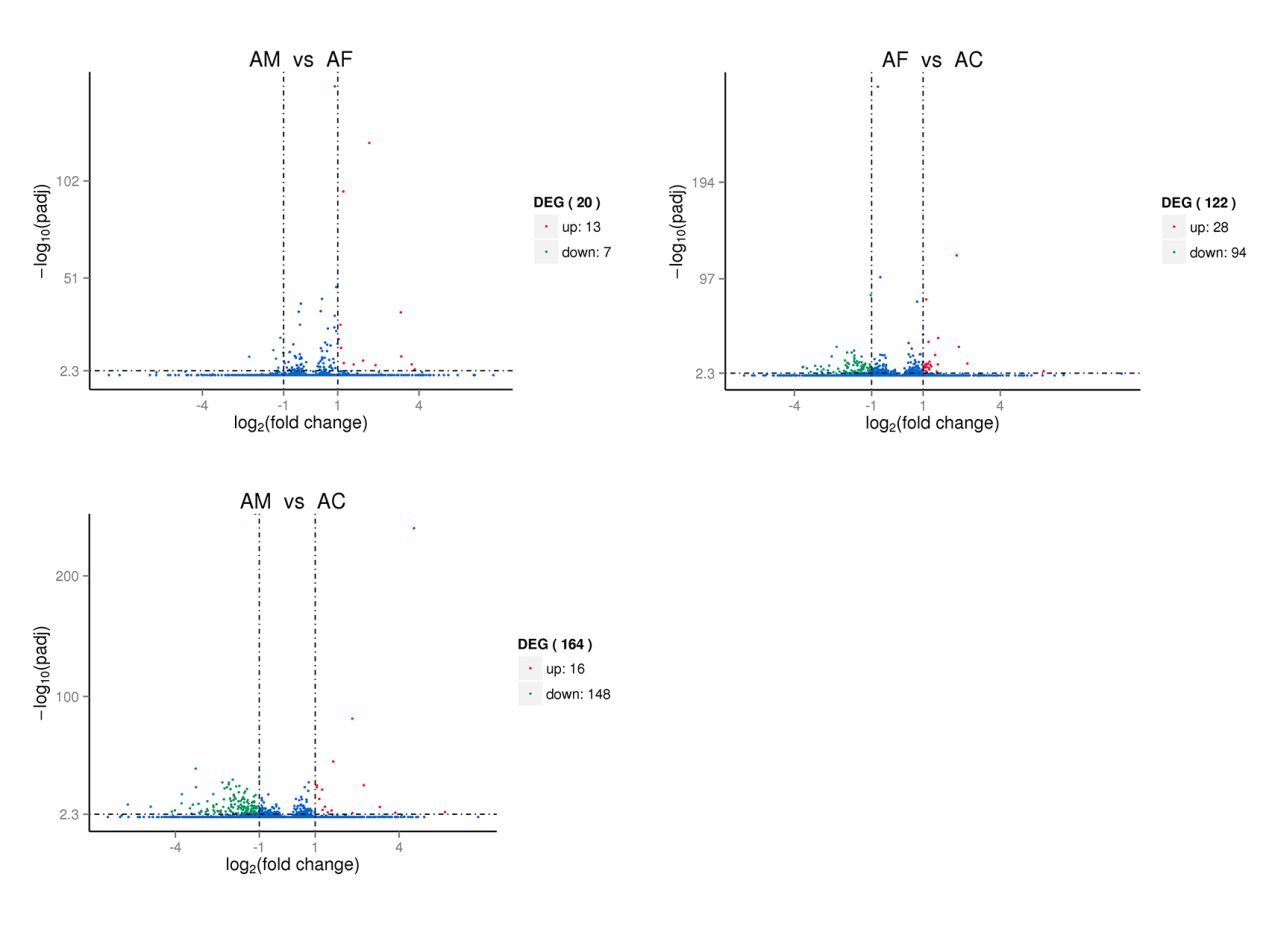


Supplementary table 1 Gene functional classification by GO

| GO Term (Lev2) | GO Term (Lev4) | Gene Number |
| --- | --- | --- |
| binding | cation binding | 3096 |
| binding | anion binding | 3884 |
| binding | vitamin binding | 52 |
| binding | organic acid binding | 57 |
| binding | nucleoside binding | 3382 |
| binding | nucleotide binding | 3812 |
| binding | alcohol binding | 4 |
| binding | chromatin DNA binding | 1 |
| binding | nucleosome binding | 1 |
| binding | monosaccharide binding | 11 |
| binding | polysaccharide binding | 12 |
| binding | protein binding, bridging | 2 |
| binding | iron-sulfur cluster binding | 64 |
| binding | coenzyme binding | 427 |
| binding | peptide binding | 11 |
| binding | nucleoside binding | 3382 |
| binding | steroid binding | 4 |
| binding | nucleoside phosphate binding | 3970 |
| binding | tetrapyrrole binding | 442 |
| binding | nucleic acid binding | 3370 |
| binding | nucleoside binding | 3382 |
| binding | nucleoside phosphate binding | 3970 |
| binding | tetrapyrrole binding | 442 |
| binding | nucleic acid binding | 3370 |
| binding | glycoprotein binding | 1 |
| binding | glycosaminoglycan binding | 3 |
| binding | ribonucleotide binding | 3424 |
| binding | nucleoside binding | 3382 |
| binding | phospholipid binding | 40 |
| binding | steroid binding | 4 |
| binding | polysaccharide binding | 12 |
| binding | cytoskeletal protein binding | 313 |
| binding | protein dimerization activity | 108 |
| binding | enzyme binding | 114 |
| binding | clathrin binding | 5 |
| binding | protein binding, bridging | 2 |
| binding | protein domain specific binding | 2 |
| binding | transcription factor binding | 9 |
| binding | SNARE binding | 3 |
| binding | protein complex binding | 183 |
| binding | glycoprotein binding | 1 |
| binding | identical protein binding | 95 |
| binding | receptor binding | 239 |
| binding | protein kinase A binding | 1 |
| binding | small conjugating protein binding | 3 |
| binding | cytokine binding | 3 |
| biological adhesion | single organism cell adhesion | 2 |
| biological adhesion | cell-substrate adhesion | 2 |
| biological adhesion | cell-cell adhesion | 9 |
| biological adhesion | adhesion of symbiont to host cell | 16 |
| biological phase | prophase | 8 |
| biological phase | M phase | 9 |
| biological regulation | negative regulation of molecular function | 54 |
| biological regulation | positive regulation of molecular function | 22 |
| biological regulation | regulation of catalytic activity | 221 |
| biological regulation | regulation of reproductive process | 2 |
| biological regulation | regulation of response to stimulus | 167 |
| biological regulation | regulation of circadian rhythm | 1 |
| biological regulation | regulation of cellular process | 3908 |
| biological regulation | regulation of multi-organism process | 44 |
| biological regulation | regulation of localization | 33 |
| biological regulation | regulation of metabolic process | 2532 |
| biological regulation | regulation of developmental process | 24 |
| biological regulation | regulation of cellular component organization | 89 |
| biological regulation | regulation of multicellular organismal process | 37 |
| biological regulation | regulation of locomotion | 14 |
| biological regulation | positive regulation of biological process | 200 |
| biological regulation | regulation of cell killing | 2 |
| biological regulation | regulation of growth | 1 |
| biological regulation | negative regulation of biological process | 180 |
| biological regulation | regulation of cellular component biogenesis | 20 |
| biological regulation | regulation of signaling | 133 |
| biological regulation | regulation of immune system process | 26 |
| biological regulation | modification of morphology or physiology of other organism | 109 |
| biological regulation | regulation of appetite | 2 |
| biological regulation | regulation of body fluid levels | 72 |
| biological regulation | regulation of protein stability | 10 |
| biological regulation | regulation of female receptivity | 4 |
| biological regulation | homeostatic process | 257 |
| biological regulation | regulation of hormone levels | 108 |
| biological regulation | regulation of anatomical structure size | 28 |
| biological regulation | maintenance of location | 64 |
| biological regulation | regulation of RNA stability | 8 |
| catalytic activity | ligase activity, forming carbon-sulfur bonds | 8 |
| catalytic activity | ligase activity, forming carbon-oxygen bonds | 144 |
| catalytic activity | ligase activity, forming carbon-nitrogen bonds | 135 |
| catalytic activity | ligase activity, forming carbon-carbon bonds | 9 |
| catalytic activity | ligase activity, forming nitrogen-metal bonds | 117 |
| catalytic activity | ligase activity, forming phosphoric ester bonds | 32 |
| catalytic activity | intramolecular transferase activity | 44 |
| catalytic activity | intramolecular lyase activity | 6 |
| catalytic activity | intramolecular oxidoreductase activity | 35 |
| catalytic activity | DNA topoisomerase activity | 26 |
| catalytic activity | racemase and epimerase activity | 28 |
| catalytic activity | cis-trans isomerase activity | 40 |
| catalytic activity | phosphorus-oxygen lyase activity | 18 |
| catalytic activity | carbon-oxygen lyase activity | 142 |
| catalytic activity | carbon-sulfur lyase activity | 2 |
| catalytic activity | carbon-carbon lyase activity | 200 |
| catalytic activity | carbon-nitrogen lyase activity | 27 |
| catalytic activity | transferase activity, transferring phosphorus-containing groups | 2662 |
| catalytic activity | transferase activity, transferring glycosyl groups | 548 |
| catalytic activity | transferase activity, transferring acyl groups | 348 |
| catalytic activity | transferase activity, transferring sulfur-containing groups | 24 |
| catalytic activity | transferase activity, transferring alkyl or aryl (other than methyl) groups | 96 |
| catalytic activity | transferase activity, transferring aldehyde or ketonic groups | 13 |
| catalytic activity | transferase activity, transferring one-carbon groups | 545 |
| catalytic activity | ubiquitin-protein transferase activity | 7 |
| catalytic activity | transferase activity, transferring nitrogenous groups | 27 |
| catalytic activity | heme-copper terminal oxidase activity | 77 |
| catalytic activity | oxidoreductase activity, acting on superoxide radicals as acceptor | 8 |
| catalytic activity | oxidoreductase activity, acting on CH or CH2 groups | 17 |
| catalytic activity | oxidoreductase activity, acting on other nitrogenous compounds as donors | 8 |
| catalytic activity | oxidoreductase activity, acting on hydrogen as donor | 2 |
| catalytic activity | oxidoreductase activity, acting on X-H and Y-H to form an X-Y bond | 1 |
| catalytic activity | oxidoreductase activity, acting on diphenols and related substances as donors | 66 |
| catalytic activity | oxidoreductase activity, acting on a sulfur group of donors | 143 |
| catalytic activity | oxidoreductase activity, acting on iron-sulfur proteins as donors | 3 |
| catalytic activity | oxidoreductase activity, acting on the aldehyde or oxo group of donors | 83 |
| catalytic activity | oxidoreductase activity, acting on single donors with incorporation of molecular oxygen | 107 |
| catalytic activity | oxidoreductase activity, acting on the CH-NH2 group of donors | 36 |
| catalytic activity | monooxygenase activity | 74 |
| catalytic activity | steroid dehydrogenase activity | 96 |
| catalytic activity | oxidoreductase activity, acting on peroxide as acceptor | 127 |
| catalytic activity | oxidoreductase activity, acting on the CH-CH group of donors | 127 |
| catalytic activity | dioxygenase activity | 230 |
| catalytic activity | oxidoreductase activity, acting on a heme group of donors | 75 |
| catalytic activity | oxidoreductase activity, acting on NAD(P)H | 158 |
| catalytic activity | oxidoreductase activity, acting on the CH-NH group of donors | 52 |
| catalytic activity | oxidoreductase activity, oxidizing metal ions | 1 |
| catalytic activity | oxidoreductase activity, acting on CH-OH group of donors | 380 |
| catalytic activity | oxidoreductase activity, acting on paired donors, with incorporation or reduction of molecular oxygen | 222 |
| catalytic activity | hydrolase activity, acting on acid halide bonds | 2 |
| catalytic activity | palmitoyl hydrolase activity | 4 |
| catalytic activity | peptidase activity | 855 |
| catalytic activity | deacetylase activity | 4 |
| catalytic activity | ubiquitinyl hydrolase activity | 51 |
| catalytic activity | hydrolase activity, acting on acid anhydrides | 1366 |
| catalytic activity | serine hydrolase activity | 348 |
| catalytic activity | hydrolase activity, acting on ether bonds | 6 |
| catalytic activity | hydrolase activity, acting on glycosyl bonds | 635 |
| catalytic activity | hydrolase activity, acting on carbon-nitrogen (but not peptide) bonds | 86 |
| catalytic activity | hydrolase activity, acting on ester bonds | 1017 |
| catalytic activity | hydrolase activity, acting on acid carbon-carbon bonds | 2 |
| catalytic activity | adenosine deaminase activity | 9 |
| catalytic activity | adenosine-phosphate deaminase activity | 2 |
| cell | dihydrolipoyl dehydrogenase complex | 1 |
| cell | NADH dehydrogenase complex | 9 |
| cell | respiratory chain complex III | 10 |
| cell | external encapsulating structure | 198 |
| cell | intracellular | 6426 |
| cell | oxidoreductase complex | 85 |
| cell | envelope | 470 |
| cell | cell periphery | 626 |
| cell | cell projection part | 20 |
| cell | cell leading edge | 2 |
| cell | plasma membrane | 221 |
| cell | cell projection | 106 |
| cell | protein serine/threonine phosphatase complex | 22 |
| cell | periplasmic space | 12 |
| cell | endomembrane system | 623 |
| cell | external encapsulating structure part | 23 |
| cell | nuclear outer membrane-endoplasmic reticulum membrane network | 160 |
| cell | plasma membrane part | 221 |
| cell | coated pit | 14 |
| cell | intracellular part | 6425 |
| cell | intracellular immature spore | 5 |
| cell junction | cell-substrate adherens junction | 4 |
| cell junction | occluding junction | 2 |
| cell junction | apical junction complex | 2 |
| cell junction | gap junction | 2 |
| cell junction | adherens junction | 4 |
| cell killing | cytolysis in other organism | 11 |
| cell killing | regulation of killing of cells of other organism | 1 |
| cell killing | killing of cells in other organism involved in symbiotic interaction | 11 |
| cell killing | positive regulation of killing of cells of other organism | 1 |
| cell killing | positive regulation of killing of cells of other organism | 1 |
| cell killing | regulation of killing of cells of other organism | 1 |
| cell killing | positive regulation of cell killing | 1 |
| cell part | bacterial-type flagellum | 17 |
| cell part | cell projection part | 20 |
| cell part | cilium | 61 |
| cell part | intracellular part | 6425 |
| cell part | cell wall | 14 |
| cell part | external encapsulating structure part | 23 |
| cell part | plasma membrane part | 221 |
| cell part | dihydrolipoyl dehydrogenase complex | 1 |
| cell part | NADH dehydrogenase complex | 9 |
| cell part | respiratory chain complex III | 10 |
| cell part | endoplasmic reticulum membrane | 107 |
| cell part | endosome | 2 |
| cell part | endoplasmic reticulum | 219 |
| cell part | coated pit | 14 |
| cell part | nuclear envelope | 94 |
| cell part | Golgi apparatus | 149 |
| cell part | transport vesicle | 48 |
| cell part | secretory granule | 4 |
| cell part | nuclear outer membrane-endoplasmic reticulum membrane network | 160 |
| cell part | organelle envelope | 436 |
| cell part | cell envelope | 34 |
| cell part | ciliary part | 3 |
| cell part | bacterial-type flagellum part | 17 |
| cell part | proteasome core complex | 9 |
| cell part | proton-transporting ATP synthase complex | 175 |
| cell part | protein acetyltransferase complex | 24 |
| cell part | organelle outer membrane | 87 |
| cell part | cytoplasm | 2235 |
| cell part | proteasome complex | 27 |
| cell part | bacterial-type flagellum part | 17 |
| cell part | methyltransferase complex | 12 |
| cell part | DNA polymerase complex | 19 |
| cell part | transcription factor complex | 43 |
| cell part | intracellular organelle | 4634 |
| cell part | RNA polymerase complex | 145 |
| cell part | Holliday junction resolvase complex | 141 |
| cell part | DNA helicase complex | 142 |
| cell part | ubiquitin ligase complex | 230 |
| cell part | ubiquitin conjugating enzyme complex | 1 |
| cell part | thylakoid part | 237 |
| cell part | proton-transporting ATP synthase complex, catalytic core F(1) | 8 |
| cell part | light-harvesting complex | 13 |
| cell part | mannosyltransferase complex | 7 |
| cell part | respiratory chain complex IV | 2 |
| cell part | extrachromosomal DNA | 8 |
| cell part | ribonucleoprotein complex | 815 |
| cell part | nonhomologous end joining complex | 4 |
| cell part | respiratory chain complex III | 10 |
| cell part | proteasome accessory complex | 7 |
| cell part | intracellular organelle part | 2600 |
| cell part | DNA polymerase processivity factor complex | 1 |
| cell part | extrinsic component of cytoplasmic side of plasma membrane | 12 |
| cell part | cytoplasmic part | 2235 |
| cell part | proton-transporting ATP synthase complex, coupling factor F(o) | 137 |
| cell part | exosome (RNase complex) | 2 |
| cell part | organelle inner membrane | 234 |
| cell part | thylakoid | 237 |
| cell part | external encapsulating structure | 198 |
| cell part | cell cortex | 85 |
| cell part | plasma membrane | 221 |
| cell part | extrinsic component of plasma membrane | 25 |
| cell part | intrinsic component of plasma membrane | 78 |
| cell part | dystroglycan complex | 18 |
| cell part | MHC protein complex | 10 |
| cell part | synaptic membrane | 9 |
| cell part | voltage-gated calcium channel complex | 5 |
| cell part | dystrophin-associated glycoprotein complex | 18 |
| cell part | integral component of plasma membrane | 63 |
| cellular component organization or biogenesis | cellular component assembly | 444 |
| cellular component organization or biogenesis | regulation of cellular component biogenesis | 20 |
| cellular component organization or biogenesis | protein complex biogenesis | 271 |
| cellular component organization or biogenesis | membrane biogenesis | 1 |
| cellular component organization or biogenesis | cellular component macromolecule biosynthetic process | 30 |
| cellular component organization or biogenesis | ribonucleoprotein complex biogenesis | 678 |
| cellular component organization or biogenesis | cell wall biogenesis | 40 |
| cellular component organization or biogenesis | positive regulation of cellular component organization | 16 |
| cellular component organization or biogenesis | regulation of cellular component size | 11 |
| cellular component organization or biogenesis | cellular component disassembly | 46 |
| cellular component organization or biogenesis | chromatin organization | 115 |
| cellular component organization or biogenesis | endomembrane system organization | 27 |
| cellular component organization or biogenesis | external encapsulating structure organization | 116 |
| cellular component organization or biogenesis | DNA packaging | 49 |
| cellular component organization or biogenesis | cellular component assembly | 444 |
| cellular component organization or biogenesis | cell projection organization | 44 |
| cellular component organization or biogenesis | membrane organization | 205 |
| cellular component organization or biogenesis | extracellular structure organization | 10 |
| cellular component organization or biogenesis | cellular component morphogenesis | 52 |
| cellular component organization or biogenesis | macromolecular complex subunit organization | 335 |
| cellular component organization or biogenesis | organelle organization | 697 |
| cellular component organization or biogenesis | regulation of cellular component organization | 89 |
| cellular component organization or biogenesis | virion assembly | 53 |
| cellular component organization or biogenesis | negative regulation of cellular component organization | 19 |
| cellular process | negative regulation of protein complex disassembly | 2 |
| cellular process | negative regulation of cellular metabolic process | 58 |
| cellular process | negative regulation of cell communication | 11 |
| cellular process | negative regulation of organelle organization | 14 |
| cellular process | negative regulation of cell cycle | 28 |
| cellular process | negative regulation of cell death | 13 |
| cellular process | negative regulation of cell cycle process | 14 |
| cellular process | negative regulation of cell projection organization | 2 |
| cellular process | negative regulation of gene silencing | 3 |
| cellular process | vitamin B6 metabolic process | 8 |
| cellular process | cell growth | 3 |
| cellular process | halogenated hydrocarbon metabolic process | 29 |
| cellular process | membrane docking | 42 |
| cellular process | ethanolamine-containing compound metabolic process | 7 |
| cellular process | electron transport chain | 218 |
| cellular process | phenylpropanoid metabolic process | 1 |
| cellular process | cellular component movement | 168 |
| cellular process | coenzyme M metabolic process | 1 |
| cellular process | microtubule-based process | 167 |
| cellular process | cellular component disassembly | 46 |
| cellular process | secretion by cell | 127 |
| cellular process | plasmid maintenance | 1 |
| cellular process | dsRNA fragmentation | 1 |
| cellular process | toxin metabolic process | 33 |
| cellular process | actin filament-based process | 45 |
| cellular process | inositol phosphate metabolic process | 7 |
| cellular process | aminoglycoside antibiotic biosynthetic process | 62 |
| cellular process | cell death | 73 |
| cellular process | chlorophyll biosynthetic process | 5 |
| cellular process | urea metabolic process | 3 |
| cellular process | protein glycosylation | 37 |
| cellular process | regulation of cellular component size | 11 |
| cellular process | energy derivation by oxidation of organic compounds | 329 |
| cellular process | inositol metabolic process | 10 |
| cellular process | cellular aldehyde metabolic process | 29 |
| cellular process | cell cycle process | 222 |
| cellular process | cellular carbohydrate biosynthetic process | 447 |
| cellular process | cell recognition | 81 |
| cellular process | alditol metabolic process | 18 |
| cellular process | cell cycle | 238 |
| cellular process | cellular potassium ion transport | 39 |
| cellular process | cobalamin metabolic process | 42 |
| cellular process | vitamin biosynthetic process | 219 |
| cellular process | DNA repair | 148 |
| cellular process | maintenance of location in cell | 64 |
| cellular process | nucleobase-containing small molecule metabolic process | 1527 |
| cellular process | cellular homeostasis | 166 |
| cellular process | single-organism organelle organization | 344 |
| cellular process | chromosome segregation | 43 |
| cellular process | heme metabolic process | 74 |
| cellular process | execution phase of apoptosis | 5 |
| cellular process | cellular lactam metabolic process | 2 |
| cellular process | extracellular structure organization | 10 |
| cellular process | transmembrane transport | 659 |
| cellular process | cell communication | 1732 |
| cellular process | thiamine-containing compound metabolic process | 30 |
| cellular process | cellular process involved in reproduction in multicellular organism | 17 |
| cellular process | disaccharide metabolic process | 382 |
| cellular process | cell projection organization | 44 |
| cellular process | cellular developmental process | 128 |
| cellular process | cellular lipid metabolic process | 961 |
| cellular process | siderophore metabolic process | 1 |
| cellular process | signal transduction by phosphorylation | 7 |
| cellular process | energy derivation by oxidation of reduced inorganic compounds | 44 |
| cellular process | organic acid metabolic process | 1509 |
| cellular process | cell activation | 63 |
| cellular process | riboflavin metabolic process | 20 |
| cellular process | cellular ketone metabolic process | 113 |
| cellular process | gene silencing | 29 |
| cellular process | phytochelatin metabolic process | 8 |
| cellular process | transposition | 111 |
| cellular process | cell division | 194 |
| cellular process | cellular alkane metabolic process | 54 |
| cellular process | one-carbon metabolic process | 1 |
| cellular process | positive regulation of cellular metabolic process | 167 |
| cellular process | negative regulation of cellular metabolic process | 58 |
| cellular process | toxin metabolic process | 33 |
| cellular process | cellular aldehyde metabolic process | 29 |
| cellular process | cellular carbohydrate metabolic process | 793 |
| cellular process | sulfur compound metabolic process | 239 |
| cellular process | organic acid metabolic process | 1509 |
| cellular process | photosynthesis | 54 |
| cellular process | cellular biosynthetic process | 5413 |
| cellular process | cellular aromatic compound metabolic process | 5847 |
| cellular process | organometal metabolic process | 3 |
| cellular process | cellular catabolic process | 1050 |
| cellular process | cellular ketone metabolic process | 113 |
| cellular process | cellular hormone metabolic process | 108 |
| cellular process | cofactor metabolic process | 728 |
| cellular process | generation of precursor metabolites and energy | 594 |
| cellular process | xenobiotic metabolic process | 41 |
| cellular process | translational initiation | 76 |
| cellular process | one-carbon metabolic process | 1 |
| cellular process | cellular alkane metabolic process | 54 |
| cellular process | cellular nitrogen compound metabolic process | 5965 |
| cellular process | prenylation | 20 |
| cellular process | thioester metabolic process | 26 |
| cellular process | cellular lipid metabolic process | 961 |
| cellular process | cellular macromolecule metabolic process | 7637 |
| cellular process | reactive oxygen species metabolic process | 20 |
| cellular process | phosphorus metabolic process | 2924 |
| cellular process | regulation of cellular metabolic process | 2370 |
| cellular process | heterocycle metabolic process | 5897 |
| cellular process | drug metabolic process | 68 |
| cellular process | multi-organism intercellular transport | 36 |
| cellular process | cytolysis in other organism | 11 |
| cellular process | homeostasis of number of cells in a free-living population | 4 |
| cellular process | genetic transfer | 5 |
| cellular process | aggregation of single cell organisms | 3 |
| cellular process | conjugation | 9 |
| cellular process | viral process | 449 |
| cellular process | cellular response to stress | 555 |
| cellular process | cellular response to extracellular stimulus | 50 |
| cellular process | cellular response to abiotic stimulus | 1 |
| cellular process | cellular response to chemical stimulus | 82 |
| cellular process | signal transduction | 878 |
| cellular process | positive regulation of protein complex disassembly | 2 |
| cellular process | positive regulation of cell differentiation | 3 |
| cellular process | positive regulation of cellular metabolic process | 167 |
| cellular process | positive regulation of cell death | 10 |
| cellular process | positive regulation of cell communication | 2 |
| cellular process | positive regulation of organelle organization | 4 |
| cellular process | regulation of cell division | 35 |
| cellular process | regulation of organelle organization | 41 |
| cellular process | negative regulation of cellular process | 124 |
| cellular process | regulation of cellular component movement | 11 |
| cellular process | regulation of actin filament-based process | 11 |
| cellular process | positive regulation of cellular process | 196 |
| cellular process | regulation of cellular metabolic process | 2370 |
| cellular process | signal transduction | 878 |
| cellular process | regulation of cellular response to stress | 3 |
| cellular process | regulation of protein complex disassembly | 33 |
| cellular process | regulation of cell morphogenesis | 1 |
| cellular process | regulation of cell proliferation | 3 |
| cellular process | regulation of microtubule-based process | 3 |
| cellular process | regulation of cellular localization | 12 |
| cellular process | regulation of vesicle-mediated transport | 11 |
| cellular process | regulation of transposition | 1 |
| cellular process | regulation of cell differentiation | 7 |
| cellular process | regulation of cell projection organization | 6 |
| cellular process | regulation of cell communication | 133 |
| cellular process | regulation of viral process | 7 |
| cellular process | modulation by symbiont of host cellular process | 21 |
| cellular process | regulation of gene silencing | 3 |
| cellular process | regulation of cell death | 47 |
| cellular process | regulation of cell cycle | 59 |
| cellular process | cytolysis in other organism | 11 |
| cellular process | regulation of organelle organization | 41 |
| cellular process | organelle inheritance | 3 |
| cellular process | organelle fission | 112 |
| cellular process | microtubule organizing center organization | 3 |
| cellular process | vesicle organization | 12 |
| cellular process | plastid organization | 6 |
| cellular process | negative regulation of organelle organization | 14 |
| cellular process | single-organism organelle organization | 344 |
| cellular process | peroxisome organization | 22 |
| cellular process | chromosome organization | 209 |
| cellular process | organelle assembly | 85 |
| cellular process | vacuole organization | 14 |
| cellular process | nucleus organization | 15 |
| cellular process | mitochondrion organization | 52 |
| cellular process | cytoskeleton organization | 148 |
| cellular process | organelle fusion | 16 |
| cellular process | positive regulation of organelle organization | 4 |
| cellular process | cell wall organization | 107 |
| cellular process | cell wall macromolecule metabolic process | 99 |
| cellular process | plant-type cell wall organization or biogenesis | 44 |
| cellular process | cell wall biogenesis | 40 |
| cellular process | cell envelope organization | 1 |
| cellular process | capsule organization | 3 |
| cellular process | cell wall organization | 107 |
| cellular process | multi-organism intercellular transport | 36 |
| channel regulator activity | ion channel inhibitor activity | 36 |
| developmental process | sex determination | 3 |
| developmental process | floral whorl development | 2 |
| developmental process | reproductive structure development | 5 |
| developmental process | seed maturation | 1 |
| developmental process | pollen tube development | 1 |
| developmental process | sex differentiation | 12 |
| developmental process | eggshell formation | 13 |
| developmental process | spermatid differentiation | 4 |
| developmental process | floral organ development | 2 |
| developmental process | seed dormancy process | 1 |
| developmental process | ovarian follicle cell development | 13 |
| developmental process | germ cell development | 17 |
| developmental process | root morphogenesis | 3 |
| developmental process | cellular component assembly involved in morphogenesis | 12 |
| developmental process | angiogenesis | 2 |
| developmental process | cellular developmental process | 128 |
| developmental process | post-embryonic development | 40 |
| developmental process | post-embryonic organ development | 2 |
| developmental process | seed dormancy process | 1 |
| developmental process | muscle structure development | 2 |
| developmental process | hematopoietic or lymphoid organ development | 1 |
| developmental process | embryo development | 14 |
| developmental process | root development | 3 |
| developmental process | regeneration | 8 |
| developmental process | floral whorl development | 2 |
| developmental process | blood vessel morphogenesis | 2 |
| developmental process | sporulation | 24 |
| developmental process | blood vessel development | 2 |
| developmental process | developmental maturation | 2 |
| developmental process | organ morphogenesis | 3 |
| developmental process | developmental growth | 11 |
| developmental process | multicellular organismal development | 97 |
| developmental process | shoot system development | 3 |
| developmental process | eggshell formation | 13 |
| developmental process | cell development | 20 |
| developmental process | phyllome development | 2 |
| developmental process | blood vessel development | 2 |
| developmental process | reproductive structure development | 5 |
| developmental process | system development | 27 |
| developmental process | floral whorl development | 2 |
| developmental process | regeneration | 8 |
| developmental process | organ development | 21 |
| developmental process | embryo development | 14 |
| developmental process | pollen tube development | 1 |
| developmental process | anatomical structure morphogenesis | 119 |
| developmental process | muscle structure development | 2 |
| developmental process | tissue development | 35 |
| developmental process | positive regulation of cell differentiation | 3 |
| developmental process | positive regulation of sporulation | 3 |
| developmental process | regulation of cell differentiation | 7 |
| developmental process | positive regulation of developmental process | 3 |
| developmental process | regulation of multicellular organismal development | 16 |
| developmental process | regulation of anatomical structure morphogenesis | 10 |
| developmental process | regulation of sporulation | 7 |
| developmental process | negative regulation of developmental process | 1 |
| developmental process | regulation of anatomical structure morphogenesis | 10 |
| developmental process | anatomical structure formation involved in morphogenesis | 38 |
| developmental process | post-embryonic morphogenesis | 35 |
| developmental process | shoot system morphogenesis | 1 |
| developmental process | developmental growth involved in morphogenesis | 3 |
| developmental process | organ morphogenesis | 3 |
| developmental process | blood vessel morphogenesis | 2 |
| developmental process | root morphogenesis | 3 |
| developmental process | cellular component morphogenesis | 52 |
| developmental process | seed dormancy process | 1 |
| developmental process | negative regulation of post-embryonic development | 1 |
| developmental process | eggshell formation | 13 |
| developmental process | cellular component assembly involved in morphogenesis | 12 |
| developmental process | angiogenesis | 2 |
| developmental process | sporulation resulting in formation of a cellular spore | 7 |
| enzyme regulator activity | kinase activator activity | 2 |
| enzyme regulator activity | protein kinase regulator activity | 22 |
| enzyme regulator activity | kinase inhibitor activity | 17 |
| enzyme regulator activity | protein phosphatase regulator activity | 26 |
| enzyme regulator activity | phosphatase inhibitor activity | 4 |
| enzyme regulator activity | GTPase regulator activity | 99 |
| enzyme regulator activity | ATPase regulator activity | 41 |
| enzyme regulator activity | kinase activator activity | 2 |
| enzyme regulator activity | GTPase activator activity | 69 |
| enzyme regulator activity | phosphatase inhibitor activity | 4 |
| enzyme regulator activity | peptidase inhibitor activity | 76 |
| enzyme regulator activity | metalloenzyme inhibitor activity | 3 |
| enzyme regulator activity | ATPase inhibitor activity | 4 |
| enzyme regulator activity | kinase inhibitor activity | 17 |
| enzyme regulator activity | peptidase inhibitor activity | 76 |
| enzyme regulator activity | endopeptidase regulator activity | 76 |
| enzyme regulator activity | metalloenzyme inhibitor activity | 3 |
| extracellular matrix | fibril | 4 |
| extracellular matrix | basal lamina | 11 |
| extracellular matrix | basement membrane | 15 |
| extracellular matrix | laminin complex | 11 |
| extracellular matrix | fibril | 4 |
| extracellular matrix | basement membrane | 15 |
| extracellular matrix part | laminin complex | 11 |
| extracellular matrix part | basal lamina | 11 |
| extracellular region | plasma lipoprotein particle | 4 |
| extracellular region | other organism part | 171 |
| extracellular region | fibril | 4 |
| extracellular region | extraorganismal space | 171 |
| extracellular region | proteinaceous extracellular matrix | 53 |
| extracellular region | extracellular space | 62 |
| extracellular region | laminin complex | 11 |
| extracellular region | basement membrane | 15 |
| extracellular region | basal lamina | 11 |
| extracellular region part | laminin complex | 11 |
| extracellular region part | basal lamina | 11 |
| extracellular region part | other organism membrane | 48 |
| extracellular region part | host cell part | 167 |
| extracellular region part | other organism cell | 171 |
| extracellular region part | basement membrane | 15 |
| extracellular region part | fibril | 4 |
| extracellular region part | plasma lipoprotein particle | 4 |
| extracellular region part | other organism | 171 |
| growth | developmental cell growth | 3 |
| growth | unidimensional cell growth | 1 |
| growth | developmental growth involved in morphogenesis | 3 |
| growth | developmental cell growth | 3 |
| immune system process | hematopoietic or lymphoid organ development | 1 |
| immune system process | activation of innate immune response | 1 |
| immune system process | immune response-activating signal transduction | 3 |
| immune system process | negative regulation of immune system process | 16 |
| immune system process | positive regulation of immune system process | 4 |
| immune system process | regulation of immune response | 4 |
| immune system process | regulation of immune effector process | 22 |
| immune system process | leukocyte degranulation | 3 |
| immune system process | leukocyte mediated immunity | 5 |
| immune system process | cell activation involved in immune response | 3 |
| immune system process | negative regulation of immune effector process | 16 |
| immune system process | regulation of immune effector process | 22 |
| immune system process | defense response to virus | 22 |
| immune system process | cell activation involved in immune response | 3 |
| immune system process | humoral immune response | 4 |
| immune system process | adaptive immune response | 2 |
| immune system process | regulation of immune response | 4 |
| immune system process | innate immune response | 7 |
| immune system process | positive regulation of immune response | 4 |
| immune system process | inflammatory response to antigenic stimulus | 2 |
| immune system process | positive regulation of immune response | 4 |
| immune system process | negative regulation of immune effector process | 16 |
| immune system process | leukocyte activation involved in immune response | 3 |
| immune system process | myeloid leukocyte activation | 3 |
| localization | single-organism cellular localization | 137 |
| localization | establishment of localization in cell | 1015 |
| localization | cellular macromolecule localization | 833 |
| localization | regulation of cellular localization | 12 |
| localization | maintenance of location in cell | 64 |
| localization | localization within membrane | 19 |
| localization | organelle localization | 2 |
| localization | maintenance of location in cell | 64 |
| localization | maintenance of protein location | 64 |
| localization | regulation of cellular component movement | 11 |
| localization | regulation of transport | 22 |
| localization | regulation of cellular localization | 12 |
| localization | regulation of protein localization | 20 |
| localization | cell motility | 29 |
| localization | single-organism cellular localization | 137 |
| localization | polysaccharide localization | 3 |
| localization | lipid localization | 57 |
| localization | RNA localization | 36 |
| localization | protein localization | 1028 |
| localization | cellular macromolecule localization | 833 |
| localization | establishment of RNA localization | 36 |
| localization | establishment of localization in cell | 1015 |
| localization | establishment of protein localization | 970 |
| localization | transport | 3362 |
| locomotion | regulation of cell motility | 11 |
| locomotion | cell migration | 11 |
| locomotion | chemotaxis | 3 |
| locomotion | entry into other organism involved in symbiotic interaction | 38 |
| locomotion | movement in other organism involved in symbiotic interaction | 36 |
| locomotion | movement in host environment | 74 |
| locomotion | regulation of cell motility | 11 |
| locomotion | dissemination or transmission of symbiont from host | 14 |
| macromolecular complex | euchromatin | 1 |
| macromolecular complex | nuclear chromatin | 2 |
| macromolecular complex | small nucleolar ribonucleoprotein complex | 8 |
| macromolecular complex | ribosomal subunit | 26 |
| macromolecular complex | signal recognition particle | 14 |
| macromolecular complex | spliceosomal complex | 8 |
| macromolecular complex | RNAi effector complex | 1 |
| macromolecular complex | small nuclear ribonucleoprotein complex | 7 |
| macromolecular complex | preribosome | 66 |
| macromolecular complex | ribosome | 34 |
| macromolecular complex | proteasome core complex | 9 |
| macromolecular complex | dystrophin-associated glycoprotein complex | 18 |
| macromolecular complex | Holliday junction resolvase complex | 141 |
| macromolecular complex | catalytic complex | 1284 |
| macromolecular complex | light-harvesting complex | 13 |
| macromolecular complex | respiratory chain complex IV | 2 |
| macromolecular complex | proton-transporting two-sector ATPase complex | 386 |
| macromolecular complex | photosystem | 181 |
| macromolecular complex | BLOC complex | 10 |
| macromolecular complex | condensed chromosome outer kinetochore | 53 |
| macromolecular complex | kinetochore | 58 |
| macromolecular complex | MHC protein complex | 10 |
| macromolecular complex | tricarboxylic acid cycle enzyme complex | 2 |
| macromolecular complex | microtubule associated complex | 80 |
| macromolecular complex | pore complex | 76 |
| macromolecular complex | microtubule | 9 |
| macromolecular complex | translocon complex | 15 |
| macromolecular complex | dystroglycan complex | 18 |
| macromolecular complex | transporter complex | 89 |
| macromolecular complex | laminin complex | 11 |
| macromolecular complex | RNA cap binding complex | 1 |
| macromolecular complex | transcription factor complex | 43 |
| macromolecular complex | proton-transporting two-sector ATPase complex, proton-transporting domain | 305 |
| macromolecular complex | intermediate filament | 6 |
| macromolecular complex | transcriptional repressor complex | 1 |
| macromolecular complex | membrane coat | 102 |
| macromolecular complex | receptor complex | 16 |
| macromolecular complex | DNA bending complex | 27 |
| macromolecular complex | proteasome complex | 27 |
| macromolecular complex | DNA packaging complex | 31 |
| macromolecular complex | transcription export complex | 14 |
| macromolecular complex | ubiquitin conjugating enzyme complex | 1 |
| macromolecular complex | nonhomologous end joining complex | 4 |
| macromolecular complex | DNA repair complex | 4 |
| macromolecular complex | proteasome accessory complex | 7 |
| macromolecular complex | proton-transporting two-sector ATPase complex, catalytic domain | 56 |
| macromolecular complex | SWI/SNF superfamily-type complex | 1 |
| macromolecular complex | DNA polymerase processivity factor complex | 1 |
| macromolecular complex | THO complex | 14 |
| macromolecular complex | cytochrome complex | 130 |
| macromolecular complex | origin recognition complex | 16 |
| macromolecular complex | exosome (RNase complex) | 2 |
| macromolecular complex | AP-type membrane coat adaptor complex | 24 |
| macromolecular complex | chromosome, centromeric region | 84 |
| macromolecular complex | plasma lipoprotein particle | 4 |
| membrane | organelle outer membrane | 87 |
| membrane | endoplasmic reticulum membrane | 107 |
| membrane | membrane raft | 2 |
| membrane | nuclear outer membrane-endoplasmic reticulum membrane network | 160 |
| membrane | cation-transporting ATPase complex | 6 |
| membrane | photosystem | 181 |
| membrane | thylakoid membrane | 37 |
| membrane | endosome membrane | 2 |
| membrane | nuclear membrane | 6 |
| membrane | plastid membrane | 3 |
| membrane | vacuolar membrane | 38 |
| membrane | endoplasmic reticulum membrane | 107 |
| membrane | organelle outer membrane | 87 |
| membrane | Golgi membrane | 74 |
| membrane | microbody membrane | 21 |
| membrane | mitochondrial membrane | 311 |
| membrane | organelle inner membrane | 234 |
| membrane | vesicle membrane | 83 |
| membrane | bounding membrane of organelle | 443 |
| membrane | extrinsic component of organelle membrane | 3 |
| membrane | endoplasmic reticulum membrane | 107 |
| membrane | cytoplasmic side of membrane | 3 |
| membrane | nuclear outer membrane-endoplasmic reticulum membrane network | 160 |
| membrane | proton-transporting two-sector ATPase complex, proton-transporting domain | 305 |
| membrane | proton-transporting two-sector ATPase complex, catalytic domain | 56 |
| membrane | ATPase dependent transmembrane transport complex | 8 |
| membrane | coated pit | 14 |
| membrane | respiratory chain complex III | 10 |
| membrane | plasma membrane part | 221 |
| membrane | pore complex | 76 |
| membrane | intrinsic component of membrane | 2704 |
| membrane | photosystem | 181 |
| membrane | ion channel complex | 49 |
| membrane | endoplasmic reticulum membrane | 107 |
| membrane | proton-transporting two-sector ATPase complex | 386 |
| membrane | respiratory chain complex IV | 2 |
| membrane | mitochondrial membrane part | 219 |
| membrane | transmembrane transporter complex | 87 |
| membrane | membrane coat | 102 |
| membrane | anchored component of membrane | 1 |
| membrane | AP-type membrane coat adaptor complex | 24 |
| membrane | respiratory chain | 94 |
| membrane | nuclear membrane part | 6 |
| membrane | integral component of membrane | 309 |
| membrane | side of membrane | 3 |
| membrane | NADH dehydrogenase complex | 9 |
| membrane | membrane region | 162 |
| membrane | translocon complex | 15 |
| membrane | extrinsic component of membrane | 28 |
| membrane | plasma membrane part | 221 |
| membrane | membrane coat | 102 |
| membrane part | intrinsic component of mitochondrial membrane | 6 |
| membrane part | mitochondrial proton-transporting ATP synthase complex | 145 |
| membrane part | mitochondrial respiratory chain | 21 |
| membrane part | integral component of mitochondrial membrane | 6 |
| membrane part | ion channel complex | 49 |
| membrane part | ATPase dependent transmembrane transport complex | 8 |
| membrane part | respiratory chain complex III | 10 |
| membrane part | extrinsic component of organelle membrane | 3 |
| membrane part | extrinsic component of plasma membrane | 25 |
| membrane part | respiratory chain complex I | 9 |
| membrane part | proton-transporting two-sector ATPase complex, proton-transporting domain | 305 |
| membrane part | proton-transporting ATP synthase complex | 175 |
| membrane part | proton-transporting V-type ATPase complex | 190 |
| membrane part | proton-transporting two-sector ATPase complex, catalytic domain | 56 |
| membrane part | membrane raft | 2 |
| membrane part | endoplasmic reticulum membrane | 107 |
| membrane part | nuclear outer membrane-endoplasmic reticulum membrane network | 160 |
| membrane part | photosystem I | 16 |
| membrane part | photosystem II | 91 |
| membrane part | cation channel complex | 49 |
| membrane part | cytoplasmic side of membrane | 3 |
| membrane part | intrinsic component of endoplasmic reticulum membrane | 45 |
| membrane part | rough endoplasmic reticulum membrane | 24 |
| membrane part | voltage-gated calcium channel complex | 5 |
| membrane part | synaptic membrane | 9 |
| membrane part | integral component of plasma membrane | 63 |
| membrane part | dystrophin-associated glycoprotein complex | 18 |
| membrane part | dystroglycan complex | 18 |
| membrane part | intrinsic component of plasma membrane | 78 |
| membrane part | extrinsic component of plasma membrane | 25 |
| membrane part | MHC protein complex | 10 |
| membrane part | intrinsic component of organelle membrane | 125 |
| membrane part | intrinsic component of plasma membrane | 78 |
| membrane part | anchored component of membrane | 1 |
| membrane part | intrinsic component of thylakoid membrane | 2 |
| membrane part | integral component of membrane | 309 |
| membrane part | proton-transporting V-type ATPase, V1 domain | 34 |
| membrane part | proton-transporting ATP synthase complex, catalytic core F(1) | 8 |
| membrane part | mitochondrial respiratory chain | 21 |
| membrane part | respiratory chain complex I | 9 |
| membrane part | respiratory chain complex III | 10 |
| membrane part | respiratory chain complex IV | 2 |
| membrane part | intrinsic component of nuclear inner membrane | 6 |
| membrane part | cation-transporting ATPase complex | 6 |
| membrane part | transmembrane transporter complex | 87 |
| membrane part | pore complex | 76 |
| membrane part | integral component of organelle membrane | 113 |
| membrane part | integral component of plasma membrane | 63 |
| membrane part | ion channel complex | 49 |
| membrane part | proton-transporting ATP synthase complex, coupling factor F(o) | 137 |
| membrane part | proton-transporting V-type ATPase, V0 domain | 1 |
| membrane part | AP-type membrane coat adaptor complex | 24 |
| membrane part | vesicle coat | 69 |
| membrane part | clathrin coat | 36 |
| membrane part | endoplasmic reticulum membrane | 107 |
| membrane-enclosed lumen | intracellular organelle lumen | 559 |
| membrane-enclosed lumen | mitochondrial intermembrane space | 2 |
| metabolic process | positive regulation of biosynthetic process | 65 |
| metabolic process | single-organism biosynthetic process | 2075 |
| metabolic process | organic substance biosynthetic process | 5606 |
| metabolic process | cellular biosynthetic process | 5413 |
| metabolic process | regulation of biosynthetic process | 2013 |
| metabolic process | negative regulation of biosynthetic process | 52 |
| metabolic process | cellular hormone metabolic process | 108 |
| metabolic process | regulation of primary metabolic process | 2315 |
| metabolic process | positive regulation of metabolic process | 167 |
| metabolic process | regulation of nitrogen compound metabolic process | 2136 |
| metabolic process | regulation of catalytic activity | 221 |
| metabolic process | negative regulation of metabolic process | 88 |
| metabolic process | regulation of cellular metabolic process | 2370 |
| metabolic process | regulation of biosynthetic process | 2013 |
| metabolic process | regulation of macromolecule metabolic process | 2256 |
| metabolic process | regulation of catabolic process | 220 |
| metabolic process | negative regulation of cellular metabolic process | 58 |
| metabolic process | negative regulation of nitrogen compound metabolic process | 56 |
| metabolic process | negative regulation of macromolecule metabolic process | 77 |
| metabolic process | negative regulation of biosynthetic process | 52 |
| metabolic process | single-organism catabolic process | 750 |
| metabolic process | regulation of catabolic process | 220 |
| metabolic process | cellular catabolic process | 1050 |
| metabolic process | positive regulation of catabolic process | 97 |
| metabolic process | organic substance catabolic process | 1141 |
| metabolic process | viral gene expression | 40 |
| metabolic process | heterocycle metabolic process | 5897 |
| metabolic process | drug metabolic process | 68 |
| metabolic process | regulation of cellular metabolic process | 2370 |
| metabolic process | reactive oxygen species metabolic process | 20 |
| metabolic process | phosphorus metabolic process | 2924 |
| metabolic process | thioester metabolic process | 26 |
| metabolic process | cellular macromolecule metabolic process | 7637 |
| metabolic process | cellular lipid metabolic process | 961 |
| metabolic process | prenylation | 20 |
| metabolic process | cellular nitrogen compound metabolic process | 5965 |
| metabolic process | one-carbon metabolic process | 1 |
| metabolic process | cellular alkane metabolic process | 54 |
| metabolic process | generation of precursor metabolites and energy | 594 |
| metabolic process | xenobiotic metabolic process | 41 |
| metabolic process | translational initiation | 76 |
| metabolic process | cofactor metabolic process | 728 |
| metabolic process | cellular hormone metabolic process | 108 |
| metabolic process | cellular ketone metabolic process | 113 |
| metabolic process | cellular catabolic process | 1050 |
| metabolic process | organometal metabolic process | 3 |
| metabolic process | organic acid metabolic process | 1509 |
| metabolic process | cellular biosynthetic process | 5413 |
| metabolic process | cellular aromatic compound metabolic process | 5847 |
| metabolic process | photosynthesis | 54 |
| metabolic process | toxin metabolic process | 33 |
| metabolic process | sulfur compound metabolic process | 239 |
| metabolic process | cellular aldehyde metabolic process | 29 |
| metabolic process | cellular carbohydrate metabolic process | 793 |
| metabolic process | negative regulation of cellular metabolic process | 58 |
| metabolic process | positive regulation of cellular metabolic process | 167 |
| metabolic process | cellular ketone metabolic process | 113 |
| metabolic process | poly(hydroxyalkanoate) metabolic process | 1 |
| metabolic process | lipid metabolic process | 1114 |
| metabolic process | cellular alkane metabolic process | 54 |
| metabolic process | carbon fixation | 21 |
| metabolic process | organophosphate metabolic process | 1357 |
| metabolic process | organic substance catabolic process | 1141 |
| metabolic process | cellular aldehyde metabolic process | 29 |
| metabolic process | fatty acid derivative metabolic process | 7 |
| metabolic process | carbohydrate derivative metabolic process | 1620 |
| metabolic process | organometal metabolic process | 3 |
| metabolic process | organic acid metabolic process | 1509 |
| metabolic process | flavonoid metabolic process | 2 |
| metabolic process | organic hydroxy compound metabolic process | 151 |
| metabolic process | olefin metabolic process | 12 |
| metabolic process | organonitrogen compound metabolic process | 2977 |
| metabolic process | ether metabolic process | 6 |
| metabolic process | coenzyme M metabolic process | 1 |
| metabolic process | glycosyl compound metabolic process | 882 |
| metabolic process | polyketide metabolic process | 60 |
| metabolic process | macromolecule metabolic process | 8438 |
| metabolic process | organic substance biosynthetic process | 5606 |
| metabolic process | carbohydrate metabolic process | 1244 |
| metabolic process | organic cyclic compound metabolic process | 6002 |
| metabolic process | thioester metabolic process | 26 |
| metabolic process | halogenated hydrocarbon metabolic process | 29 |
| metabolic process | macromolecule methylation | 154 |
| metabolic process | nucleobase-containing compound metabolic process | 5417 |
| metabolic process | carbohydrate metabolic process | 1244 |
| metabolic process | regulation of primary metabolic process | 2315 |
| metabolic process | cellular amino acid metabolic process | 1164 |
| metabolic process | tricarboxylic acid cycle | 21 |
| metabolic process | protein metabolic process | 4112 |
| metabolic process | lipid metabolic process | 1114 |
| metabolic process | nitric oxide metabolic process | 5 |
| metabolic process | negative regulation of nitrogen compound metabolic process | 56 |
| metabolic process | cellular nitrogen compound metabolic process | 5965 |
| metabolic process | reactive nitrogen species metabolic process | 9 |
| metabolic process | positive regulation of nitrogen compound metabolic process | 63 |
| metabolic process | nitrogen cycle metabolic process | 23 |
| metabolic process | organonitrogen compound metabolic process | 2977 |
| metabolic process | regulation of nitrogen compound metabolic process | 2136 |
| metabolic process | dsRNA fragmentation | 1 |
| metabolic process | cellular aldehyde metabolic process | 29 |
| metabolic process | oxidation-reduction process | 507 |
| metabolic process | fatty acid derivative metabolic process | 7 |
| metabolic process | chromosome condensation | 4 |
| metabolic process | lipid metabolic process | 1114 |
| metabolic process | secondary metabolic process | 108 |
| metabolic process | carbon fixation | 21 |
| metabolic process | cellular alkane metabolic process | 54 |
| metabolic process | single-organism catabolic process | 750 |
| metabolic process | telomere maintenance | 3 |
| metabolic process | DNA repair | 148 |
| metabolic process | pigment metabolic process | 276 |
| metabolic process | small molecule metabolic process | 2953 |
| metabolic process | halogenated hydrocarbon metabolic process | 29 |
| metabolic process | histone modification | 23 |
| metabolic process | single-organism biosynthetic process | 2075 |
| metabolic process | single-organism carbohydrate metabolic process | 1166 |
| metabolic process | coenzyme M metabolic process | 1 |
| metabolic process | glycosyl compound metabolic process | 882 |
| metabolic process | signal transduction by phosphorylation | 7 |
| metabolic process | positive regulation of nitrogen compound metabolic process | 63 |
| metabolic process | positive regulation of cellular metabolic process | 167 |
| metabolic process | positive regulation of catabolic process | 97 |
| metabolic process | positive regulation of biosynthetic process | 65 |
| metabolic process | positive regulation of macromolecule metabolic process | 68 |
| molecular transducer activity | receptor signaling protein activity | 49 |
| molecular transducer activity | signaling receptor activity | 216 |
| multicellular organismal process | regulation of body fluid levels | 72 |
| multicellular organismal process | negative regulation of multicellular organismal process | 1 |
| multicellular organismal process | cell recognition | 81 |
| multicellular organismal process | post-embryonic development | 40 |
| multicellular organismal process | seed germination | 2 |
| multicellular organismal process | molting cycle | 8 |
| multicellular organismal process | molting cycle process | 8 |
| multicellular organismal process | system process | 69 |
| multicellular organismal process | tissue remodeling | 1 |
| multicellular organismal process | multicellular organismal development | 97 |
| multicellular organismal process | vascular transport | 1 |
| multicellular organismal process | seed dormancy process | 1 |
| multicellular organismal process | coagulation | 72 |
| multicellular organismal process | adult behavior | 2 |
| multicellular organismal process | cellular process involved in reproduction in multicellular organism | 17 |
| multicellular organismal process | multicellular organismal reproductive process | 42 |
| multicellular organismal process | pollination | 76 |
| multicellular organismal process | multicellular organismal reproductive behavior | 4 |
| multi-organism process | modification of morphology or physiology of other organism involved in symbiotic interaction | 106 |
| multi-organism process | modulation of programmed cell death in other organism | 9 |
| multi-organism process | disruption of cells of other organism | 14 |
| multi-organism process | mating | 7 |
| multi-organism process | sexual reproduction | 54 |
| multi-organism process | multi-organism reproductive behavior | 7 |
| multi-organism process | pollination | 76 |
| multi-organism process | viral process | 449 |
| multi-organism process | conjugation | 9 |
| multi-organism process | aggregation of single cell organisms | 3 |
| multi-organism process | genetic transfer | 5 |
| multi-organism process | homeostasis of number of cells in a free-living population | 4 |
| multi-organism process | cytolysis in other organism | 11 |
| multi-organism process | multi-organism intercellular transport | 36 |
| multi-organism process | positive regulation of killing of cells of other organism | 1 |
| multi-organism process | adhesion of symbiont to host cell | 16 |
| multi-organism process | pollination | 76 |
| multi-organism process | multicellular organismal reproductive behavior | 4 |
| multi-organism process | negative regulation of defense response to virus | 16 |
| multi-organism process | viral gene expression | 40 |
| multi-organism process | regulation of killing of cells of other organism | 1 |
| multi-organism process | regulation of symbiosis, encompassing mutualism through parasitism | 15 |
| multi-organism process | negative regulation of multi-organism process | 16 |
| multi-organism process | regulation of defense response to virus | 22 |
| multi-organism process | positive regulation of multi-organism process | 1 |
| multi-organism process | multi-organism reproductive behavior | 7 |
| multi-organism process | response to bacterium | 94 |
| multi-organism process | response to symbiont | 1 |
| multi-organism process | response to defenses of other organism involved in symbiotic interaction | 23 |
| multi-organism process | response to host | 23 |
| multi-organism process | defense response to other organism | 120 |
| multi-organism process | response to virus | 50 |
| multi-organism process | response to fungus | 10 |
| multi-organism process | multi-organism membrane fusion | 27 |
| multi-organism process | transport of virus | 47 |
| multi-organism process | multi-organism intercellular transport | 36 |
| multi-organism process | multi-organism intracellular transport | 11 |
| multi-organism process | interaction with host | 198 |
| multi-organism process | symbiosis, encompassing mutualism through parasitism | 614 |
| negative regulation of biological process | negative regulation of protein transport | 8 |
| negative regulation of biological process | negative regulation of endocytosis | 1 |
| negative regulation of biological process | negative regulation of post-embryonic development | 1 |
| negative regulation of biological process | negative regulation of signal transduction | 9 |
| negative regulation of biological process | negative regulation of immune effector process | 16 |
| negative regulation of biological process | negative regulation of protein complex disassembly | 2 |
| negative regulation of biological process | negative regulation of organelle assembly | 2 |
| negative regulation of biological process | negative regulation of organelle organization | 14 |
| negative regulation of biological process | negative regulation of endocytosis | 1 |
| negative regulation of biological process | negative regulation of cell projection organization | 2 |
| negative regulation of biological process | negative regulation of defense response to virus | 16 |
| negative regulation of biological process | negative regulation of signal transduction | 9 |
| negative regulation of biological process | negative regulation of response to biotic stimulus | 16 |
| negative regulation of biological process | negative regulation of defense response | 16 |
| negative regulation of biological process | negative regulation of response to external stimulus | 18 |
| negative regulation of biological process | negative regulation of protein complex disassembly | 2 |
| negative regulation of biological process | negative regulation of organelle organization | 14 |
| negative regulation of biological process | negative regulation of cell communication | 11 |
| negative regulation of biological process | negative regulation of cellular metabolic process | 58 |
| negative regulation of biological process | negative regulation of cell cycle | 28 |
| negative regulation of biological process | negative regulation of cell death | 13 |
| negative regulation of biological process | negative regulation of cell projection organization | 2 |
| negative regulation of biological process | negative regulation of cell cycle process | 14 |
| negative regulation of biological process | negative regulation of gene silencing | 3 |
| negative regulation of biological process | negative regulation of post-embryonic development | 1 |
| negative regulation of biological process | negative regulation of cellular metabolic process | 58 |
| negative regulation of biological process | negative regulation of nitrogen compound metabolic process | 56 |
| negative regulation of biological process | negative regulation of macromolecule metabolic process | 77 |
| negative regulation of biological process | negative regulation of biosynthetic process | 52 |
| organelle | intracellular non-membrane-bounded organelle | 1976 |
| organelle | bacterial-type flagellum | 17 |
| organelle | intracellular organelle part | 2600 |
| organelle | ciliary part | 3 |
| organelle | bacterial-type flagellum part | 17 |
| organelle | intrinsic component of organelle membrane | 125 |
| organelle | organelle lumen | 559 |
| organelle | organelle membrane | 694 |
| organelle | integral component of organelle membrane | 113 |
| organelle | organellar ribosome | 5 |
| organelle | contractile fiber part | 1 |
| organelle | intracellular organelle part | 2600 |
| organelle | intracellular non-membrane-bounded organelle | 1976 |
| organelle | intracellular membrane-bounded organelle | 3313 |
| organelle | cytoplasmic vesicle | 107 |
| organelle | intracellular membrane-bounded organelle | 3313 |
| organelle | organelle subcompartment | 11 |
| organelle | organelle envelope | 436 |
| organelle | organelle membrane | 694 |
| organelle | cilium | 61 |
| organelle | membrane-bounded vesicle | 102 |
| organelle | cytoplasmic vesicle | 107 |
| organelle part | intrinsic component of nuclear inner membrane | 6 |
| organelle part | integral component of organelle membrane | 113 |
| organelle part | intrinsic component of peroxisomal membrane | 18 |
| organelle part | intrinsic component of Golgi membrane | 38 |
| organelle part | intrinsic component of endoplasmic reticulum membrane | 45 |
| organelle part | intrinsic component of mitochondrial membrane | 6 |
| organelle part | bacterial-type flagellum basal body | 2 |
| organelle part | bacterial-type flagellum basal body, distal rod | 2 |
| organelle part | bacterial-type flagellum basal body, rod | 2 |
| organelle part | mitochondrial membrane | 311 |
| organelle part | organelle inner membrane | 234 |
| organelle part | microbody membrane | 21 |
| organelle part | bounding membrane of organelle | 443 |
| organelle part | vesicle membrane | 83 |
| organelle part | Golgi membrane | 74 |
| organelle part | extrinsic component of organelle membrane | 3 |
| organelle part | endosome membrane | 2 |
| organelle part | plastid membrane | 3 |
| organelle part | nuclear membrane | 6 |
| organelle part | organelle outer membrane | 87 |
| organelle part | endoplasmic reticulum membrane | 107 |
| organelle part | vacuolar membrane | 38 |
| organelle part | integral component of mitochondrial membrane | 6 |
| organelle part | integral component of Golgi membrane | 7 |
| organelle part | integral component of endoplasmic reticulum membrane | 9 |
| organelle part | organelle subcompartment | 11 |
| organelle part | cytoskeletal part | 402 |
| organelle part | microbody part | 21 |
| organelle part | endosomal part | 2 |
| organelle part | plastid part | 136 |
| organelle part | nuclear part | 897 |
| organelle part | endoplasmic reticulum part | 218 |
| organelle part | vacuolar part | 38 |
| organelle part | mitochondrial part | 349 |
| organelle part | extrinsic component of organelle membrane | 3 |
| organelle part | ribosomal subunit | 26 |
| organelle part | organelle envelope | 436 |
| organelle part | chromosomal part | 732 |
| organelle part | organelle envelope lumen | 7 |
| organelle part | intracellular organelle lumen | 559 |
| organelle part | Golgi apparatus part | 148 |
| organelle part | cytoplasmic vesicle part | 91 |
| organelle part | intracellular organelle lumen | 559 |
| organelle part | organellar small ribosomal subunit | 1 |
| organelle part | mitochondrial ribosome | 1 |
| organelle part | ciliary transition zone | 3 |
| organelle part | sarcomere | 1 |
| organelle part | myofilament | 1 |
| positive regulation of biological process | positive regulation of protein transport | 10 |
| positive regulation of biological process | positive regulation of intracellular transport | 10 |
| positive regulation of biological process | positive regulation of immune response | 4 |
| positive regulation of biological process | positive regulation of immune response | 4 |
| positive regulation of biological process | positive regulation of defense response | 1 |
| positive regulation of biological process | positive regulation of signal transduction | 2 |
| positive regulation of biological process | positive regulation of killing of cells of other organism | 1 |
| positive regulation of biological process | positive regulation of cellular metabolic process | 167 |
| positive regulation of biological process | positive regulation of catabolic process | 97 |
| positive regulation of biological process | positive regulation of biosynthetic process | 65 |
| positive regulation of biological process | positive regulation of macromolecule metabolic process | 68 |
| positive regulation of biological process | positive regulation of nitrogen compound metabolic process | 63 |
| positive regulation of biological process | positive regulation of organelle organization | 4 |
| positive regulation of biological process | positive regulation of establishment of protein localization to plasma membrane | 10 |
| positive regulation of biological process | positive regulation of protein complex disassembly | 2 |
| positive regulation of biological process | positive regulation of protein complex assembly | 4 |
| positive regulation of biological process | positive regulation of organelle organization | 4 |
| positive regulation of biological process | positive regulation of cell communication | 2 |
| positive regulation of biological process | positive regulation of cell death | 10 |
| positive regulation of biological process | positive regulation of protein complex disassembly | 2 |
| positive regulation of biological process | positive regulation of cell differentiation | 3 |
| positive regulation of biological process | positive regulation of cellular metabolic process | 167 |
| positive regulation of biological process | positive regulation of cell differentiation | 3 |
| positive regulation of biological process | positive regulation of sporulation | 3 |
| positive regulation of biological process | positive regulation of killing of cells of other organism | 1 |
| positive regulation of biological process | positive regulation of signal transduction | 2 |
| protein binding transcription factor activity | RNA polymerase II transcription factor binding transcription factor activity | 126 |
| protein binding transcription factor activity | transcription cofactor activity | 141 |
| protein binding transcription factor activity | core DNA-dependent RNA polymerase binding promoter specificity activity | 60 |
| receptor activity | peptide receptor activity | 10 |
| receptor activity | steroid hormone receptor activity | 8 |
| receptor activity | photoreceptor activity | 2 |
| receptor activity | transmembrane signaling receptor activity | 123 |
| regulation of biological process | regulation of cell motility | 11 |
| regulation of biological process | regulation of coagulation | 2 |
| regulation of biological process | regulation of hemostasis | 2 |
| regulation of biological process | regulation of multicellular organismal development | 16 |
| regulation of biological process | negative regulation of multicellular organismal process | 1 |
| regulation of biological process | regulation of system process | 19 |
| regulation of biological process | negative regulation of response to stimulus | 29 |
| regulation of biological process | positive regulation of response to stimulus | 6 |
| regulation of biological process | regulation of behavior | 3 |
| regulation of biological process | regulation of response to biotic stimulus | 22 |
| regulation of biological process | regulation of response to stress | 28 |
| regulation of biological process | regulation of signal transduction | 117 |
| regulation of biological process | regulation of response to external stimulus | 29 |
| regulation of biological process | regulation of immune response | 4 |
| regulation of biological process | regulation of anatomical structure morphogenesis | 10 |
| regulation of biological process | regulation of sporulation | 7 |
| regulation of biological process | negative regulation of developmental process | 1 |
| regulation of biological process | regulation of cell differentiation | 7 |
| regulation of biological process | regulation of multicellular organismal development | 16 |
| regulation of biological process | positive regulation of developmental process | 3 |
| regulation of biological process | regulation of cell projection organization | 6 |
| regulation of biological process | negative regulation of cellular component organization | 19 |
| regulation of biological process | regulation of protein localization to plasma membrane | 10 |
| regulation of biological process | regulation of protein complex disassembly | 33 |
| regulation of biological process | regulation of endocytosis | 1 |
| regulation of biological process | regulation of cell morphogenesis | 1 |
| regulation of biological process | regulation of organelle organization | 41 |
| regulation of biological process | regulation of protein complex assembly | 11 |
| regulation of biological process | regulation of cell septum assembly | 3 |
| regulation of biological process | positive regulation of cellular component organization | 16 |
| regulation of biological process | regulation of seed development | 1 |
| regulation of biological process | negative regulation of cellular process | 124 |
| regulation of biological process | regulation of actin filament-based process | 11 |
| regulation of biological process | regulation of cellular component movement | 11 |
| regulation of biological process | positive regulation of cellular process | 196 |
| regulation of biological process | signal transduction | 878 |
| regulation of biological process | regulation of cellular metabolic process | 2370 |
| regulation of biological process | regulation of organelle organization | 41 |
| regulation of biological process | regulation of cell division | 35 |
| regulation of biological process | regulation of protein complex disassembly | 33 |
| regulation of biological process | regulation of cellular response to stress | 3 |
| regulation of biological process | regulation of transposition | 1 |
| regulation of biological process | regulation of cell differentiation | 7 |
| regulation of biological process | regulation of cell morphogenesis | 1 |
| regulation of biological process | regulation of cell proliferation | 3 |
| regulation of biological process | regulation of vesicle-mediated transport | 11 |
| regulation of biological process | regulation of cellular localization | 12 |
| regulation of biological process | regulation of microtubule-based process | 3 |
| regulation of biological process | modulation by symbiont of host cellular process | 21 |
| regulation of biological process | regulation of gene silencing | 3 |
| regulation of biological process | regulation of cell death | 47 |
| regulation of biological process | regulation of cell cycle | 59 |
| regulation of biological process | regulation of cell communication | 133 |
| regulation of biological process | regulation of cell projection organization | 6 |
| regulation of biological process | regulation of viral process | 7 |
| regulation of biological process | positive regulation of developmental process | 3 |
| regulation of biological process | positive regulation of cellular process | 196 |
| regulation of biological process | positive regulation of cellular component organization | 16 |
| regulation of biological process | positive regulation of response to stimulus | 6 |
| regulation of biological process | positive regulation of multi-organism process | 1 |
| regulation of biological process | positive regulation of immune system process | 4 |
| regulation of biological process | positive regulation of cell killing | 1 |
| regulation of biological process | positive regulation of signaling | 2 |
| regulation of biological process | positive regulation of metabolic process | 167 |
| regulation of biological process | positive regulation of transport | 10 |
| regulation of biological process | positive regulation of cell killing | 1 |
| regulation of biological process | regulation of killing of cells of other organism | 1 |
| regulation of biological process | negative regulation of multicellular organismal process | 1 |
| regulation of biological process | negative regulation of metabolic process | 88 |
| regulation of biological process | negative regulation of signaling | 11 |
| regulation of biological process | negative regulation of developmental process | 1 |
| regulation of biological process | negative regulation of multi-organism process | 16 |
| regulation of biological process | negative regulation of immune system process | 16 |
| regulation of biological process | negative regulation of cellular component organization | 19 |
| regulation of biological process | negative regulation of cellular process | 124 |
| regulation of biological process | negative regulation of transport | 9 |
| regulation of biological process | negative regulation of response to stimulus | 29 |
| regulation of biological process | regulation of protein localization | 20 |
| regulation of biological process | regulation of cellular localization | 12 |
| regulation of biological process | regulation of transport | 22 |
| regulation of biological process | regulation of cellular component movement | 11 |
| regulation of biological process | regulation of killing of cells of other organism | 1 |
| regulation of biological process | regulation of symbiosis, encompassing mutualism through parasitism | 15 |
| regulation of biological process | regulation of defense response to virus | 22 |
| regulation of biological process | negative regulation of multi-organism process | 16 |
| regulation of biological process | positive regulation of multi-organism process | 1 |
| regulation of biological process | regulation of cell septum assembly | 3 |
| regulation of biological process | regulation of protein complex assembly | 11 |
| regulation of biological process | regulation of cell projection assembly | 6 |
| regulation of biological process | regulation of organelle assembly | 6 |
| regulation of biological process | regulation of signal transduction | 117 |
| regulation of biological process | receptor recycling | 2 |
| regulation of biological process | positive regulation of signaling | 2 |
| regulation of biological process | negative regulation of signaling | 11 |
| regulation of biological process | regulation of immune response | 4 |
| regulation of biological process | regulation of immune effector process | 22 |
| regulation of biological process | negative regulation of immune system process | 16 |
| regulation of biological process | positive regulation of immune system process | 4 |
| regulation of biological process | regulation of cellular metabolic process | 2370 |
| regulation of biological process | negative regulation of metabolic process | 88 |
| regulation of biological process | regulation of nitrogen compound metabolic process | 2136 |
| regulation of biological process | regulation of catalytic activity | 221 |
| regulation of biological process | regulation of catabolic process | 220 |
| regulation of biological process | regulation of macromolecule metabolic process | 2256 |
| regulation of biological process | regulation of biosynthetic process | 2013 |
| regulation of biological process | regulation of primary metabolic process | 2315 |
| regulation of biological process | positive regulation of metabolic process | 167 |
| reproduction | meiotic nuclear division | 40 |
| reproduction | meiotic cell cycle process | 40 |
| reproduction | fertilization | 7 |
| reproduction | mating | 7 |
| reproduction | gamete generation | 32 |
| reproduction | cellular process involved in reproduction in multicellular organism | 17 |
| reproduction | multicellular organismal reproductive process | 42 |
| reproductive process | post-mating behavior | 4 |
| reproductive process | multi-organism reproductive behavior | 7 |
| reproductive process | pollination | 76 |
| reproductive process | multi-organism reproductive behavior | 7 |
| reproductive process | sexual reproduction | 54 |
| reproductive process | mating | 7 |
| reproductive process | floral organ development | 2 |
| reproductive process | gamete generation | 32 |
| reproductive process | seed dormancy process | 1 |
| reproductive process | ovarian follicle cell development | 13 |
| reproductive process | germ cell development | 17 |
| reproductive process | fertilization | 7 |
| reproductive process | eggshell formation | 13 |
| reproductive process | mating type determination | 3 |
| reproductive process | spermatid differentiation | 4 |
| reproductive process | floral whorl development | 2 |
| reproductive process | seed development | 2 |
| reproductive process | seed maturation | 1 |
| reproductive process | reproductive shoot system development | 2 |
| reproductive process | regulation of seed development | 1 |
| reproductive process | gamete generation | 32 |
| reproductive process | multicellular organismal reproductive behavior | 4 |
| reproductive process | seed dormancy process | 1 |
| reproductive process | seed maturation | 1 |
| reproductive process | pollen tube development | 1 |
| reproductive process | sex differentiation | 12 |
| reproductive process | eggshell formation | 13 |
| reproductive process | spermatid differentiation | 4 |
| reproductive process | floral organ development | 2 |
| reproductive process | seed dormancy process | 1 |
| reproductive process | ovarian follicle cell development | 13 |
| reproductive process | germ cell development | 17 |
| reproductive process | sex determination | 3 |
| reproductive process | floral whorl development | 2 |
| reproductive process | reproductive structure development | 5 |
| reproductive process | seed maturation | 1 |
| response to stimulus | cellular response to chemical stimulus | 82 |
| response to stimulus | response to toxic substance | 26 |
| response to stimulus | response to food | 2 |
| response to stimulus | response to arsenic-containing substance | 1 |
| response to stimulus | detection of chemical stimulus | 2 |
| response to stimulus | response to drug | 67 |
| response to stimulus | response to oxygen-containing compound | 17 |
| response to stimulus | response to xenobiotic stimulus | 41 |
| response to stimulus | response to transition metal nanoparticle | 6 |
| response to stimulus | response to acid | 17 |
| response to stimulus | response to nitrogen compound | 3 |
| response to stimulus | response to inorganic substance | 31 |
| response to stimulus | chemotaxis | 3 |
| response to stimulus | response to organic substance | 85 |
| response to stimulus | cellular response to stress | 555 |
| response to stimulus | response to oxidative stress | 5 |
| response to stimulus | response to wounding | 80 |
| response to stimulus | response to starvation | 18 |
| response to stimulus | response to water deprivation | 1 |
| response to stimulus | regulation of response to stress | 28 |
| response to stimulus | response to osmotic stress | 4 |
| response to stimulus | response to topologically incorrect protein | 5 |
| response to stimulus | defense response | 169 |
| response to stimulus | adaptive immune response | 2 |
| response to stimulus | humoral immune response | 4 |
| response to stimulus | cell activation involved in immune response | 3 |
| response to stimulus | inflammatory response to antigenic stimulus | 2 |
| response to stimulus | innate immune response | 7 |
| response to stimulus | positive regulation of immune response | 4 |
| response to stimulus | regulation of immune response | 4 |
| response to stimulus | response to hormone | 27 |
| response to stimulus | response to organonitrogen compound | 2 |
| response to stimulus | cellular response to endogenous stimulus | 28 |
| response to stimulus | detection of external stimulus | 8 |
| response to stimulus | detection of stimulus involved in sensory perception | 2 |
| response to stimulus | detection of chemical stimulus | 2 |
| response to stimulus | detection of abiotic stimulus | 8 |
| response to stimulus | response to pH | 1 |
| response to stimulus | response to osmotic stress | 4 |
| response to stimulus | cellular response to abiotic stimulus | 1 |
| response to stimulus | response to temperature stimulus | 9 |
| response to stimulus | detection of abiotic stimulus | 8 |
| response to stimulus | response to oxygen levels | 1 |
| response to stimulus | response to water | 1 |
| response to stimulus | response to radiation | 28 |
| response to stimulus | positive regulation of immune response | 4 |
| response to stimulus | positive regulation of signal transduction | 2 |
| response to stimulus | positive regulation of defense response | 1 |
| response to stimulus | cellular response to stress | 555 |
| response to stimulus | cellular response to extracellular stimulus | 50 |
| response to stimulus | cellular response to chemical stimulus | 82 |
| response to stimulus | signal transduction | 878 |
| response to stimulus | cellular response to abiotic stimulus | 1 |
| response to stimulus | regulation of behavior | 3 |
| response to stimulus | feeding behavior | 2 |
| response to stimulus | multi-organism behavior | 7 |
| response to stimulus | single-organism behavior | 2 |
| response to stimulus | reproductive behavior | 7 |
| response to stimulus | rhythmic behavior | 1 |
| response to stimulus | regulation of response to external stimulus | 29 |
| response to stimulus | regulation of immune response | 4 |
| response to stimulus | regulation of signal transduction | 117 |
| response to stimulus | positive regulation of response to stimulus | 6 |
| response to stimulus | negative regulation of response to stimulus | 29 |
| response to stimulus | regulation of response to stress | 28 |
| response to stimulus | regulation of behavior | 3 |
| response to stimulus | regulation of response to biotic stimulus | 22 |
| response to stimulus | negative regulation of response to external stimulus | 18 |
| response to stimulus | negative regulation of defense response | 16 |
| response to stimulus | negative regulation of signal transduction | 9 |
| response to stimulus | negative regulation of response to biotic stimulus | 16 |
| response to stimulus | regulation of response to external stimulus | 29 |
| response to stimulus | negative regulation of response to external stimulus | 18 |
| response to stimulus | response to external biotic stimulus | 159 |
| response to stimulus | detection of external stimulus | 8 |
| response to stimulus | cellular response to external stimulus | 50 |
| response to stimulus | response to extracellular stimulus | 50 |
| response to stimulus | taxis | 27 |
| response to stimulus | negative regulation of response to biotic stimulus | 16 |
| response to stimulus | regulation of response to biotic stimulus | 22 |
| response to stimulus | response to external biotic stimulus | 159 |
| rhythmic process | circadian behavior | 1 |
| rhythmic process | regulation of circadian rhythm | 1 |
| rhythmic process | circadian behavior | 1 |
| signaling | regulation of signaling | 133 |
| signaling | cell-cell signaling | 59 |
| signaling | signal transduction | 878 |
| signaling | positive regulation of signaling | 2 |
| signaling | negative regulation of signaling | 11 |
| single-organism process | reproductive shoot system development | 2 |
| single-organism process | seed maturation | 1 |
| single-organism process | seed development | 2 |
| single-organism process | floral whorl development | 2 |
| single-organism process | spermatid differentiation | 4 |
| single-organism process | fertilization | 7 |
| single-organism process | eggshell formation | 13 |
| single-organism process | mating type determination | 3 |
| single-organism process | gamete generation | 32 |
| single-organism process | seed dormancy process | 1 |
| single-organism process | ovarian follicle cell development | 13 |
| single-organism process | germ cell development | 17 |
| single-organism process | floral organ development | 2 |
| single-organism process | cell cycle process | 222 |
| single-organism process | cellular aldehyde metabolic process | 29 |
| single-organism process | inositol metabolic process | 10 |
| single-organism process | cellular carbohydrate biosynthetic process | 447 |
| single-organism process | cell cycle | 238 |
| single-organism process | alditol metabolic process | 18 |
| single-organism process | cell recognition | 81 |
| single-organism process | cellular potassium ion transport | 39 |
| single-organism process | chlorophyll biosynthetic process | 5 |
| single-organism process | urea metabolic process | 3 |
| single-organism process | energy derivation by oxidation of organic compounds | 329 |
| single-organism process | regulation of cellular component size | 11 |
| single-organism process | protein glycosylation | 37 |
| single-organism process | toxin metabolic process | 33 |
| single-organism process | dsRNA fragmentation | 1 |
| single-organism process | secretion by cell | 127 |
| single-organism process | plasmid maintenance | 1 |
| single-organism process | actin filament-based process | 45 |
| single-organism process | cell death | 73 |
| single-organism process | aminoglycoside antibiotic biosynthetic process | 62 |
| single-organism process | inositol phosphate metabolic process | 7 |
| single-organism process | vitamin B6 metabolic process | 8 |
| single-organism process | membrane docking | 42 |
| single-organism process | halogenated hydrocarbon metabolic process | 29 |
| single-organism process | cell growth | 3 |
| single-organism process | cellular component movement | 168 |
| single-organism process | phenylpropanoid metabolic process | 1 |
| single-organism process | electron transport chain | 218 |
| single-organism process | ethanolamine-containing compound metabolic process | 7 |
| single-organism process | microtubule-based process | 167 |
| single-organism process | cellular component disassembly | 46 |
| single-organism process | coenzyme M metabolic process | 1 |
| single-organism process | energy derivation by oxidation of reduced inorganic compounds | 44 |
| single-organism process | riboflavin metabolic process | 20 |
| single-organism process | cell activation | 63 |
| single-organism process | organic acid metabolic process | 1509 |
| single-organism process | gene silencing | 29 |
| single-organism process | cellular ketone metabolic process | 113 |
| single-organism process | one-carbon metabolic process | 1 |
| single-organism process | cellular alkane metabolic process | 54 |
| single-organism process | cell division | 194 |
| single-organism process | transposition | 111 |
| single-organism process | phytochelatin metabolic process | 8 |
| single-organism process | disaccharide metabolic process | 382 |
| single-organism process | cellular lipid metabolic process | 961 |
| single-organism process | cellular developmental process | 128 |
| single-organism process | cell projection organization | 44 |
| single-organism process | siderophore metabolic process | 1 |
| single-organism process | signal transduction by phosphorylation | 7 |
| single-organism process | transmembrane transport | 659 |
| single-organism process | extracellular structure organization | 10 |
| single-organism process | cellular process involved in reproduction in multicellular organism | 17 |
| single-organism process | thiamine-containing compound metabolic process | 30 |
| single-organism process | cell communication | 1732 |
| single-organism process | DNA repair | 148 |
| single-organism process | cobalamin metabolic process | 42 |
| single-organism process | vitamin biosynthetic process | 219 |
| single-organism process | chromosome segregation | 43 |
| single-organism process | single-organism organelle organization | 344 |
| single-organism process | cellular homeostasis | 166 |
| single-organism process | nucleobase-containing small molecule metabolic process | 1527 |
| single-organism process | maintenance of location in cell | 64 |
| single-organism process | heme metabolic process | 74 |
| single-organism process | cellular lactam metabolic process | 2 |
| single-organism process | execution phase of apoptosis | 5 |
| single-organism process | single-organism biosynthetic process | 2075 |
| single-organism process | histone modification | 23 |
| single-organism process | single-organism carbohydrate metabolic process | 1166 |
| single-organism process | glycosyl compound metabolic process | 882 |
| single-organism process | coenzyme M metabolic process | 1 |
| single-organism process | signal transduction by phosphorylation | 7 |
| single-organism process | DNA repair | 148 |
| single-organism process | pigment metabolic process | 276 |
| single-organism process | telomere maintenance | 3 |
| single-organism process | small molecule metabolic process | 2953 |
| single-organism process | halogenated hydrocarbon metabolic process | 29 |
| single-organism process | chromosome condensation | 4 |
| single-organism process | secondary metabolic process | 108 |
| single-organism process | lipid metabolic process | 1114 |
| single-organism process | cellular alkane metabolic process | 54 |
| single-organism process | carbon fixation | 21 |
| single-organism process | single-organism catabolic process | 750 |
| single-organism process | oxidation-reduction process | 507 |
| single-organism process | dsRNA fragmentation | 1 |
| single-organism process | cellular aldehyde metabolic process | 29 |
| single-organism process | fatty acid derivative metabolic process | 7 |
| single-organism process | adult behavior | 2 |
| single-organism process | single-organism cellular localization | 137 |
| single-organism process | organic acid transport | 131 |
| single-organism process | xenobiotic transport | 2 |
| single-organism process | gas transport | 1 |
| single-organism process | drug transport | 67 |
| single-organism process | ion transport | 1460 |
| single-organism process | carbohydrate derivative transport | 24 |
| single-organism process | organic hydroxy compound transport | 3 |
| single-organism process | lipid transport | 8 |
| single-organism process | hydrogen transport | 613 |
| single-organism process | iron coordination entity transport | 25 |
| single-organism process | protein import | 84 |
| single-organism process | sulfur compound transport | 22 |
| single-organism process | organophosphate ester transport | 2 |
| single-organism process | cofactor transport | 25 |
| single-organism process | phagocytosis | 1 |
| single-organism process | amine transport | 1 |
| single-organism process | vascular transport | 1 |
| single-organism process | one-carbon compound transport | 3 |
| single-organism process | amide transport | 8 |
| single-organism process | single-organism intracellular transport | 419 |
| single-organism process | vitamin transport | 7 |
| single-organism process | secretion | 127 |
| single-organism process | transmembrane transport | 659 |
| single-organism process | carbohydrate transport | 49 |
| single-organism process | cell death | 73 |
| single-organism process | regulation of cell proliferation | 3 |
| single-organism process | negative regulation of signaling | 11 |
| single-organism process | positive regulation of signaling | 2 |
| single-organism process | cell-cell signaling | 59 |
| single-organism process | signal transduction | 878 |
| single-organism process | regulation of signaling | 133 |
| single-organism process | shoot system development | 3 |
| single-organism process | eggshell formation | 13 |
| single-organism process | developmental maturation | 2 |
| single-organism process | organ morphogenesis | 3 |
| single-organism process | developmental growth | 11 |
| single-organism process | multicellular organismal development | 97 |
| single-organism process | blood vessel development | 2 |
| single-organism process | root development | 3 |
| single-organism process | floral whorl development | 2 |
| single-organism process | blood vessel morphogenesis | 2 |
| single-organism process | regeneration | 8 |
| single-organism process | sporulation | 24 |
| single-organism process | embryo development | 14 |
| single-organism process | seed dormancy process | 1 |
| single-organism process | muscle structure development | 2 |
| single-organism process | hematopoietic or lymphoid organ development | 1 |
| single-organism process | cellular developmental process | 128 |
| single-organism process | post-embryonic development | 40 |
| single-organism process | post-embryonic organ development | 2 |
| single-organism process | root morphogenesis | 3 |
| single-organism process | cellular component assembly involved in morphogenesis | 12 |
| single-organism process | angiogenesis | 2 |
| single-organism process | adult behavior | 2 |
| single-organism process | coagulation | 72 |
| single-organism process | seed dormancy process | 1 |
| single-organism process | vascular transport | 1 |
| single-organism process | multicellular organismal development | 97 |
| single-organism process | tissue remodeling | 1 |
| single-organism process | system process | 69 |
| single-organism process | molting cycle process | 8 |
| single-organism process | molting cycle | 8 |
| single-organism process | seed germination | 2 |
| single-organism process | post-embryonic development | 40 |
| single-organism process | cell recognition | 81 |
| single-organism process | negative regulation of multicellular organismal process | 1 |
| single-organism process | regulation of body fluid levels | 72 |
| single-organism process | membrane assembly | 1 |
| single-organism process | protein localization to membrane | 133 |
| single-organism process | single-organism membrane fusion | 16 |
| single-organism process | plastid membrane organization | 6 |
| single-organism process | mitochondrial membrane organization | 10 |
| single-organism process | plasma membrane organization | 10 |
| structural molecule activity | extracellular matrix structural constituent conferring compression resistance | 2 |
| synapse | synaptic membrane | 9 |
| transporter activity | iron chelate transmembrane transporter activity | 1 |
| transporter activity | passive transmembrane transporter activity | 241 |
| transporter activity | drug transmembrane transporter activity | 2 |
| transporter activity | phosphate transmembrane transporter activity | 18 |
| transporter activity | hydrolase activity, acting on acid anhydrides, catalyzing transmembrane movement of substances | 169 |
| transporter activity | organic hydroxy compound transmembrane transporter activity | 5 |
| transporter activity | active transmembrane transporter activity | 392 |
| transporter activity | efflux transmembrane transporter activity | 1 |
| transporter activity | vitamin transmembrane transporter activity | 2 |
| transporter activity | sulfur compound transmembrane transporter activity | 22 |
| transporter activity | substrate-specific transmembrane transporter activity | 1271 |
| transporter activity | nucleoside transmembrane transporter activity | 21 |
| transporter activity | purine ribonucleotide transmembrane transporter activity | 12 |
| transporter activity | neurotransmitter:sodium symporter activity | 2 |
| transporter activity | azole transporter activity | 2 |
| transporter activity | drug transmembrane transporter activity | 2 |
| transporter activity | vitamin transmembrane transporter activity | 2 |
| transporter activity | lipid transporter activity | 15 |
| transporter activity | substrate-specific transmembrane transporter activity | 1271 |
| transporter activity | protein transporter activity | 7 |
| transporter activity | carbohydrate transmembrane transporter activity | 57 |
| virion | viral membrane | 242 |
| virion | viral capsid | 52 |
| virion part | icosahedral viral capsid | 1 |

Supplementary table 2 Gene functional classification by KEGG

| KEGG Pathway | Gene Number |
| --- | --- |
| Apoptosis | 68 |
| Cell cycle | 140 |
| Cell cycle - Caulobacter | 25 |
| Cell cycle - yeast | 88 |
| Meiosis - yeast | 90 |
| Oocyte meiosis | 120 |
| p53 signaling pathway | 41 |
| Bacterial chemotaxis | 2 |
| Regulation of actin cytoskeleton | 93 |
| Adherens junction | 41 |
| Focal adhesion | 60 |
| Gap junction | 55 |
| Tight junction | 31 |
| Endocytosis | 246 |
| Lysosome | 95 |
| Peroxisome | 111 |
| Phagosome | 122 |
| Regulation of autophagy | 41 |
| ABC transporters | 69 |
| Bacterial secretion system | 12 |
| AMPK signaling pathway | 140 |
| Calcium signaling pathway | 46 |
| ErbB signaling pathway | 35 |
| FoxO signaling pathway | 81 |
| HIF-1 signaling pathway | 78 |
| Hedgehog signaling pathway | 17 |
| Hippo signaling pathway | 50 |
| Hippo signaling pathway -fly | 31 |
| Jak-STAT signaling pathway | 4 |
| MAPK signaling pathway | 76 |
| MAPK signaling pathway - fly | 21 |
| MAPK signaling pathway - yeast | 8 |
| NF-kappa B signaling pathway | 66 |
| Notch signaling pathway | 13 |
| PI3K-Akt signaling pathway | 150 |
| Phosphatidylinositol signaling system | 73 |
| Plant hormone signal transduction | 311 |
| Rap1 signaling pathway | 47 |
| Ras signaling pathway | 83 |
| TGF-beta signaling pathway | 47 |
| TNF signaling pathway | 26 |
| Two-component system | 15 |
| VEGF signaling pathway | 38 |
| Wnt signaling pathway | 69 |
| cAMP signaling pathway | 91 |
| cGMP - PKG signaling pathway | 63 |
| mTOR signaling pathway | 69 |
| Cell adhesion molecules (CAMs) | 1 |
| Neuroactive ligand-receptor interaction | 7 |
| Proteasome | 56 |
| Protein export | 40 |
| Protein processing in endoplasmic reticulum | 242 |
| RNA degradation | 113 |
| SNARE interactions in vesicular transport | 46 |
| Sulfur relay system | 10 |
| Ubiquitin mediated proteolysis | 140 |
| Base excision repair | 53 |
| DNA replication | 80 |
| Fanconi anemia pathway | 54 |
| Homologous recombination | 56 |
| Mismatch repair | 47 |
| Non-homologous end-joining | 9 |
| Nucleotide excision repair | 76 |
| Basal transcription factors | 48 |
| RNA polymerase | 55 |
| Spliceosome | 202 |
| Aminoacyl-tRNA biosynthesis | 62 |
| RNA transport | 169 |
| Ribosome | 327 |
| Ribosome biogenesis in eukaryotes | 88 |
| mRNA surveillance pathway | 138 |
| Alanine, aspartate and glutamate metabolism | 63 |
| Arginine and proline metabolism | 101 |
| Cysteine and methionine metabolism | 107 |
| Glycine, serine and threonine metabolism | 76 |
| Histidine metabolism | 29 |
| Lysine biosynthesis | 13 |
| Lysine degradation | 47 |
| Phenylalanine metabolism | 136 |
| Phenylalanine, tyrosine and tryptophan biosynthesis | 48 |
| Tryptophan metabolism | 48 |
| Tyrosine metabolism | 65 |
| Valine, leucine and isoleucine biosynthesis | 22 |
| Valine, leucine and isoleucine degradation | 48 |
| Aflatoxin biosynthesis | 1 |
| Anthocyanin biosynthesis | 2 |
| Betalain biosynthesis | 4 |
| Butirosin and neomycin biosynthesis | 8 |
| Caffeine metabolism | 5 |
| Flavone and flavonol biosynthesis | 6 |
| Flavonoid biosynthesis | 39 |
| Glucosinolate biosynthesis | 8 |
| Indole alkaloid biosynthesis | 1 |
| Isoflavonoid biosynthesis | 10 |
| Isoquinoline alkaloid biosynthesis | 32 |
| Novobiocin biosynthesis | 5 |
| Phenylpropanoid biosynthesis | 233 |
| Stilbenoid, diarylheptanoid and gingerol biosynthesis | 22 |
| Streptomycin biosynthesis | 16 |
| Tropane, piperidine and pyridine alkaloid biosynthesis | 30 |
| Amino sugar and nucleotide sugar metabolism | 169 |
| Ascorbate and aldarate metabolism | 53 |
| Butanoate metabolism | 26 |
| C5-Branched dibasic acid metabolism | 10 |
| Citrate cycle (TCA cycle) | 80 |
| Fructose and mannose metabolism | 70 |
| Galactose metabolism | 77 |
| Glycolysis / Gluconeogenesis | 187 |
| Glyoxylate and dicarboxylate metabolism | 67 |
| Inositol phosphate metabolism | 81 |
| Pentose and glucuronate interconversions | 111 |
| Pentose phosphate pathway | 81 |
| Propanoate metabolism | 25 |
| Pyruvate metabolism | 139 |
| Starch and sucrose metabolism | 292 |
| Carbon fixation in photosynthetic organisms | 106 |
| Carbon fixation pathways in prokaryotes | 62 |
| Methane metabolism | 106 |
| Nitrogen metabolism | 36 |
| Oxidative phosphorylation | 188 |
| Photosynthesis | 65 |
| Photosynthesis - antenna proteins | 21 |
| Sulfur metabolism | 40 |
| Glycosaminoglycan biosynthesis - chondroitin sulfate / dermatan sulfate | 2 |
| Glycosaminoglycan biosynthesis - heparan sulfate / heparin | 7 |
| Glycosaminoglycan degradation | 21 |
| Glycosphingolipid biosynthesis - ganglio series | 6 |
| Glycosphingolipid biosynthesis - globo series | 13 |
| Glycosylphosphatidylinositol(GPI)-anchor biosynthesis | 23 |
| Lipopolysaccharide biosynthesis | 8 |
| N-Glycan biosynthesis | 51 |
| Other glycan degradation | 27 |
| Other types of O-glycan biosynthesis | 9 |
| Peptidoglycan biosynthesis | 2 |
| Various types of N-glycan biosynthesis | 38 |
| Arachidonic acid metabolism | 16 |
| Biosynthesis of unsaturated fatty acids | 35 |
| Cutin, suberine and wax biosynthesis | 26 |
| Ether lipid metabolism | 59 |
| Fatty acid biosynthesis | 51 |
| Fatty acid degradation | 77 |
| Fatty acid elongation | 32 |
| Glycerolipid metabolism | 80 |
| Glycerophospholipid metabolism | 135 |
| Linoleic acid metabolism | 44 |
| Sphingolipid metabolism | 32 |
| Steroid biosynthesis | 29 |
| Steroid hormone biosynthesis | 3 |
| Synthesis and degradation of ketone bodies | 10 |
| alpha-Linolenic acid metabolism | 64 |
| Biotin metabolism | 19 |
| Folate biosynthesis | 18 |
| Lipoic acid metabolism | 4 |
| Nicotinate and nicotinamide metabolism | 26 |
| One carbon pool by folate | 26 |
| Pantothenate and CoA biosynthesis | 36 |
| Porphyrin and chlorophyll metabolism | 66 |
| Retinol metabolism | 49 |
| Riboflavin metabolism | 14 |
| Thiamine metabolism | 16 |
| Ubiquinone and other terpenoid-quinone biosynthesis | 50 |
| Vitamin B6 metabolism | 10 |
| Cyanoamino acid metabolism | 99 |
| D-Glutamine and D-glutamate metabolism | 3 |
| Glutathione metabolism | 118 |
| Phosphonate and phosphinate metabolism | 11 |
| Selenocompound metabolism | 20 |
| Taurine and hypotaurine metabolism | 15 |
| beta-Alanine metabolism | 64 |
| Biosynthesis of ansamycins | 3 |
| Biosynthesis of siderophore group nonribosomal peptides | 1 |
| Brassinosteroid biosynthesis | 11 |
| Carotenoid biosynthesis | 36 |
| Diterpenoid biosynthesis | 47 |
| Geraniol degradation | 2 |
| Limonene and pinene degradation | 20 |
| Monoterpenoid biosynthesis | 16 |
| Polyketide sugar unit biosynthesis | 2 |
| Sesquiterpenoid and triterpenoid biosynthesis | 15 |
| Terpenoid backbone biosynthesis | 56 |
| Tetracycline biosynthesis | 10 |
| Zeatin biosynthesis | 24 |
| Purine metabolism | 194 |
| Pyrimidine metabolism | 155 |
| 2-Oxocarboxylic acid metabolism | 67 |
| Biosynthesis of amino acids | 300 |
| Carbon metabolism | 349 |
| Degradation of aromatic compounds | 30 |
| Fatty acid metabolism | 85 |
| Aminobenzoate degradation | 10 |
| Benzoate degradation | 6 |
| Bisphenol degradation | 6 |
| Chloroalkane and chloroalkene degradation | 39 |
| Chlorocyclohexane and chlorobenzene degradation | 1 |
| Drug metabolism - cytochrome P450 | 82 |
| Drug metabolism - other enzymes | 23 |
| Fluorobenzoate degradation | 1 |
| Metabolism of xenobiotics by cytochrome P450 | 79 |
| Naphthalene degradation | 25 |
| Polycyclic aromatic hydrocarbon degradation | 6 |
| Styrene degradation | 4 |
| Toluene degradation | 1 |
| Adrenergic signaling in cardiomyocytes | 63 |
| Cardiac muscle contraction | 28 |
| Vascular smooth muscle contraction | 39 |
| Axon guidance | 51 |
| Dorso-ventral axis formation | 21 |
| Osteoclast differentiation | 38 |
| Bile secretion | 60 |
| Carbohydrate digestion and absorption | 12 |
| Fat digestion and absorption | 7 |
| Gastric acid secretion | 5 |
| Mineral absorption | 24 |
| Pancreatic secretion | 43 |
| Protein digestion and absorption | 6 |
| Salivary secretion | 5 |
| Adipocytokine signaling pathway | 52 |
| Estrogen signaling pathway | 80 |
| GnRH signaling pathway | 65 |
| Insulin secretion | 1 |
| Insulin signaling pathway | 149 |
| Melanogenesis | 39 |
| Oxytocin signaling pathway | 80 |
| PPAR signaling pathway | 46 |
| Progesterone-mediated oocyte maturation | 75 |
| Prolactin signaling pathway | 33 |
| Renin-angiotensin system | 1 |
| Thyroid hormone signaling pathway | 77 |
| Thyroid hormone synthesis | 20 |
| Circadian entrainment | 24 |
| Circadian rhythm | 39 |
| Circadian rhythm - fly | 11 |
| Circadian rhythm - plant | 58 |
| Plant-pathogen interaction | 223 |
| Aldosterone-regulated sodium reabsorption | 18 |
| Collecting duct acid secretion | 29 |
| Endocrine and other factor-regulated calcium reabsorption | 45 |
| Proximal tubule bicarbonate reclamation | 10 |
| Vasopressin-regulated water reabsorption | 45 |
| Antigen processing and presentation | 87 |
| B cell receptor signaling pathway | 47 |
| Chemokine signaling pathway | 41 |
| Cytosolic DNA-sensing pathway | 25 |
| Fc epsilon RI signaling pathway | 30 |
| Fc gamma R-mediated phagocytosis | 104 |
| Leukocyte transendothelial migration | 14 |
| NOD-like receptor signaling pathway | 38 |
| Natural killer cell mediated cytotoxicity | 38 |
| Platelet activation | 34 |
| RIG-I-like receptor signaling pathway | 7 |
| T cell receptor signaling pathway | 40 |
| Toll-like receptor signaling pathway | 86 |
| Cholinergic synapse | 23 |
| Dopaminergic synapse | 58 |
| GABAergic synapse | 36 |
| Glutamatergic synapse | 72 |
| Long-term depression | 29 |
| Long-term potentiation | 47 |
| Neurotrophin signaling pathway | 121 |
| Retrograde endocannabinoid signaling | 34 |
| Serotonergic synapse | 23 |
| Synaptic vesicle cycle | 90 |
| Inflammatory mediator regulation of TRP channels | 16 |
| Olfactory transduction | 5 |
| Phototransduction | 7 |
| Phototransduction - fly | 11 |
| Taste transduction | 2 |

Supplementary table 3 Genes involve in plant hormone signal transduction pathway, plant-pathogen interaction pathway and plant hormones biosynthesis pathway.

| Pathway | Pathway ID | KO | KO Definition | UniGene |
| --- | --- | --- | --- | --- |
| plant hormone signal transduction | ko04075 | K14486 | auxin response factor | 14 |
|  |  | K14487 | auxin responsive GH3 gene family | 8 |
|  |  | K14484 | auxin-responsive protein IAA | 29 |
|  |  | K14485 | transport inhibitor response 1 | 3 |
|  |  | K14488 | SAUR family protein | 38 |
|  |  | K14489 | arabidopsis histidine kinase 2/3/4 (cytokinin receptor) | 9 |
|  |  | K13449 | pathogenesis-related protein 1 | 7 |
|  |  | K13946 | auxin influx carrier (AUX1 LAX family) | 14 |
|  |  | K13415 | protein brassinosteroid insensitive 1 | 4 |
|  |  | K13416 | brassinosteroid insensitive 1-associated receptor kinase 1 | 5 |
|  |  | K14432 | ABA responsive element binding factor | 8 |
|  |  | K14431 | transcription factor TGA | 15 |
|  |  | K14510 | serine/threonine-protein kinase CTR1 | 2 |
|  |  | K14512 | mitogen-activated protein kinase 6 | 1 |
|  |  | K14513 | ethylene-insensitive protein 2 | 3 |
|  |  | K14514 | ethylene-insensitive protein 3 | 2 |
|  |  | K14515 | EIN3-binding F-box protein | 5 |
|  |  | K14516 | ethylene-responsive transcription factor 1 | 8 |
|  |  | K14495 | F-box protein GID2 | 1 |
|  |  | K14494 | DELLA protein | 5 |
|  |  | K14497 | protein phosphatase 2C | 9 |
|  |  | K14496 | abscisic acid receptor PYR/PYL family | 11 |
|  |  | K14491 | two-component response regulator ARR-B family | 9 |
|  |  | K14490 | histidine-containing phosphotransfer peotein | 8 |
|  |  | K14493 | gibberellin receptor GID1 | 2 |
|  |  | K14492 | two-component response regulator ARR-A family | 9 |
|  |  | K14499 | BRI1 kinase inhibitor 1 | 2 |
|  |  | K16189 | phytochrome-interacting factor 4 | 1 |
|  |  | K14498 | serine/threonine-protein kinase SRK2 | 21 |
|  |  | K14506 | jasmonic acid-amino synthetase | 4 |
|  |  | K14505 | cyclin D3, plant | 5 |
|  |  | K14504 | xyloglucan:xyloglucosyl transferase TCH4 | 2 |
|  |  | K14503 | brassinosteroid resistant 1/2 | 3 |
|  |  | K14502 | protein brassinosteroid insensitive 2 | 3 |
|  |  | K14500 | BR-signaling kinase | 7 |
|  |  | K14509 | ethylene receptor | 6 |
|  |  | K14508 | regulatory protein NPR1 | 6 |
|  |  | K13464 | jasmonate ZIM domain-containing protein | 8 |
|  |  | K12126 | phytochrome-interacting factor 3 | 7 |
|  |  | K13463 | coronatine-insensitive protein 1 | 3 |
|  |  | K13422 | transcription factor MYC2 | 4 |
| Plant-pathogen interaction | ko04626 | K12795 | suppressor of G2 allele of SKP1 | 4 |
|  |  | K02358 | elongation factor Tu | 7 |
|  |  | K13462 | guanine nucleotide-exchange factor | 1 |
|  |  | K13414 | mitogen-activated protein kinase kinase kinase 1, plant | 6 |
|  |  | K13416 | brassinosteroid insensitive 1-associated receptor kinase 1 | 5 |
|  |  | K09487 | heat shock protein 90kDa beta | 5 |
|  |  | K13412 | calcium-dependent protein kinase | 28 |
|  |  | K13413 | mitogen-activated protein kinase kinase 4/5, plant | 1 |
|  |  | K13456 | RPM1-interacting protein 4 | 1 |
|  |  | K13457 | disease resistance protein RPM1 | 19 |
|  |  | K13458 | disease resistance protein | 1 |
|  |  | K05391 | cyclic nucleotide gated channel, other eukaryote | 40 |
|  |  | K13430 | serine/threonine-protein kinase PBS1 | 3 |
|  |  | K02183 | calmodulin | 5 |
|  |  | K13463 | coronatine-insensitive protein 1 | 3 |
|  |  | K04079 | molecular chaperone HtpG | 12 |
|  |  | K00864 | glycerol kinase | 1 |
|  |  | K13429 | chitin elicitor receptor kinase 1 | 9 |
|  |  | K13464 | jasmonate ZIM domain-containing protein | 8 |
|  |  | K04368 | mitogen-activated protein kinase kinase 1 | 4 |
|  |  | K13449 | pathogenesis-related protein 1 | 7 |
|  |  | K13448 | calcium-binding protein CML | 30 |
|  |  | K13447 | respiratory burst oxidase | 9 |
|  |  | K13420 | LRR receptor-like serine/threonine-protein kinase FLS2 | 2 |
|  |  | K13422 | transcription factor MYC2 | 4 |
|  |  | K13425 | WRKY transcription factor 22 | 4 |
|  |  | K13424 | WRKY transcription factor 33 | 3 |
|  |  | K13427 | nitric-oxide synthase, plant | 1 |
| Tryptophan metabolism | ko00380 | K03781 | catalase | 3 |
|  |  | K11816 | indole-3-pyruvate monooxygenase | 12 |
|  |  | K16903 | L-tryptophan---pyruvate aminotransferase | 5 |
|  |  | K00164 | 2-oxoglutarate dehydrogenase E1 component | 9 |
|  |  | K00128 | aldehyde dehydrogenase (NAD+) | 14 |
|  |  | K14085 | aldehyde dehydrogenase family 7 member A1 | 1 |
|  |  | K00626 | acetyl-CoA C-acetyltransferase | 2 |
|  |  | K11820 | N-hydroxythioamide S-beta-glucosyltransferase | 2 |
| Zeatin biosynthesis | ko00908 | K13495 | cis-zeatin O-glucosyltransferase | 2 |
|  |  | K00279 | cytokinin dehydrogenase | 15 |
|  |  | K10717 | cytokinin trans-hydroxylase | 1 |
|  |  | K00791 | tRNA dimethylallyltransferase | 2 |
|  |  | K10760 | adenylate isopentenyltransferase (cytokinin synthase) | 4 |
| Diterpenoid biosynthesis | ko00904 | K17961 | cytochrome P450, family 82, subfamily G, polypeptide 1 | 3 |
|  |  | K04124 | gibberellin 3-beta-dioxygenase | 5 |
|  |  | K04125 | gibberellin 2-oxidase | 7 |
|  |  | K04120 | ent-copalyl diphosphate synthase | 6 |
|  |  | K04121 | ent-kaurene synthase | 1 |
|  |  | K04122 | ent-kaurene oxidase | 1 |
|  |  | K04123 | ent-kaurenoic acid hydroxylase | 12 |
|  |  | K05282 | gibberellin 20-oxidase | 12 |
| Carotenoid biosynthesis | ko00906 | K17912 | 9-cis-beta-carotene 9',10'-cleaving dioxygenase | 1 |
|  |  | K14593 | capsanthin/capsorubin synthase | 1 |
|  |  | K15746 | beta-carotene 3-hydroxylase | 1 |
|  |  | K15747 | beta-ring hydroxylase | 1 |
|  |  | K15744 | zeta-carotene isomerase | 1 |
|  |  | K09843 | (+)-abscisic acid 8'-hydroxylase | 6 |
|  |  | K17911 | beta-carotene isomerase | 1 |
|  |  | K02291 | phytoene synthase | 4 |
|  |  | K17913 | carlactone synthase / all-trans-10'-apo-beta-carotenal 13,14-cleaving dioxygenase | 1 |
|  |  | K02293 | 15-cis-phytoene desaturase | 1 |
|  |  | K06444 | lycopene epsilon-cyclase | 2 |
|  |  | K09835 | prolycopene isomerase | 1 |
|  |  | K09837 | carotene epsilon-monooxygenase | 1 |
|  |  | K09840 | 9-cis-epoxycarotenoid dioxygenase | 5 |
|  |  | K06443 | lycopene beta-cyclase | 1 |
|  |  | K09838 | zeaxanthin epoxidase | 2 |
|  |  | K09839 | violaxanthin de-epoxidase | 2 |
|  |  | K00514 | zeta-carotene desaturase | 1 |
|  |  | K09842 | abscisic-aldehyde oxidase | 3 |
| Cysteine and methionine metabolism | ko00270 | K00899 | 5-methylthioribose kinase | 2 |
|  |  | K01244 | 5'-methylthioadenosine nucleosidase | 1 |
|  |  | K16054 | methylthioribulose 1-phosphate dehydratase / enolase-phosphatase E1 | 1 |
|  |  | K01011 | thiosulfate/3-mercaptopyruvate sulfurtransferase | 1 |
|  |  | K00811 | aspartate aminotransferase, chloroplastic | 1 |
|  |  | K00815 | tyrosine aminotransferase | 4 |
|  |  | K01761 | methionine-gamma-lyase | 1 |
|  |  | K01760 | cystathionine beta-lyase | 1 |
|  |  | K01762 | 1-aminocyclopropane-1-carboxylate synthase | 7 |
|  |  | K05933 | aminocyclopropanecarboxylate oxidase | 9 |
|  |  | K00133 | aspartate-semialdehyde dehydrogenase | 1 |
|  |  | K14454 | aspartate aminotransferase, cytoplasmic | 1 |
|  |  | K00640 | serine O-acetyltransferase | 7 |
|  |  | K00547 | homocysteine S-methyltransferase | 2 |
|  |  | K12524 | bifunctional aspartokinase / homoserine dehydrogenase 1 | 1 |
|  |  | K00789 | S-adenosylmethionine synthetase | 9 |
|  |  | K01739 | cystathionine gamma-synthase | 1 |
|  |  | K00016 | L-lactate dehydrogenase | 1 |
|  |  | K00549 | 5-methyltetrahydropteroyltriglutamate--homocysteine methyltransferase | 6 |
|  |  | K08967 | 1,2-dihydroxy-3-keto-5-methylthiopentene dioxygenase | 4 |
|  |  | K01251 | adenosylhomocysteinase | 4 |
|  |  | K08963 | methylthioribose-1-phosphate isomerase | 1 |
|  |  | K00025 | malate dehydrogenase | 3 |
|  |  | K01611 | S-adenosylmethionine decarboxylase | 6 |
|  |  | K00928 | aspartate kinase | 1 |
|  |  | K14455 | aspartate aminotransferase, mitochondrial | 2 |
|  |  | K01738 | cysteine synthase A | 11 |
|  |  | K17398 | DNA (cytosine-5)-methyltransferase 3A | 1 |
|  |  | K00797 | spermidine synthase | 8 |
|  |  | K13034 | L-3-cyanoalanine synthase/ cysteine synthase | 2 |
|  |  | K00026 | malate dehydrogenase | 4 |
|  |  | K00558 | DNA (cytosine-5)-methyltransferase 1 | 3 |
| Brassinosteroid biosynthesis | ko00905 | K09588 | cytochrome P450, family 90, subfamily A, polypeptide 1 | 3 |
|  |  | K15639 | PHYB activation tagged suppressor 1 | 1 |
|  |  | K12637 | 3-epi-6-deoxocathasterone 23-monooxygenase | 1 |
|  |  | K09587 | steroid 22-alpha-hydroxylase | 1 |
|  |  | K12640 | brassinosteroid-6-oxidase 2 | 4 |
|  |  | K09591 | steroid 5-alpha-reductase | 1 |
| alpha-Linolenic acid metabolism | ko00592 | K10527 | enoyl-CoA hydratase/3-hydroxyacyl-CoA dehydrogenase | 5 |
|  |  | K05894 | 12-oxophytodienoic acid reductase | 5 |
|  |  | K16818 | phospholipase A1 | 1 |
|  |  | K08241 | jasmonate O-methyltransferase | 9 |
|  |  | K00232 | acyl-CoA oxidase | 5 |
|  |  | K01047 | secretory phospholipase A2 | 1 |
|  |  | K00454 | lipoxygenase | 17 |
|  |  | K10528 | hydroperoxide lyase | 2 |
|  |  | K01723 | hydroperoxide dehydratase | 4 |
|  |  | K10526 | OPC-8:0 CoA ligase 1 | 5 |
|  |  | K10529 | alpha-dioxygenase | 1 |
|  |  | K14674 | TAG lipase / steryl ester hydrolase / phospholipase A2 / LPA acyltransferase | 2 |
|  |  | K07513 | acetyl-CoA acyltransferase 1 | 3 |
|  |  | K10525 | allene oxide cyclase | 4 |
| Phenylalanine metabolism | ko00360 | K00276 | primary-amine oxidase | 8 |
|  |  | K14455 | aspartate aminotransferase, mitochondrial | 2 |
|  |  | K14454 | aspartate aminotransferase, cytoplasmic | 1 |
|  |  | K00074 | 3-hydroxybutyryl-CoA dehydrogenase | 1 |
|  |  | K00457 | 4-hydroxyphenylpyruvate dioxygenase | 1 |
|  |  | K15849 | bifunctional aspartate aminotransferase and glutamate/aspartate-prephenate aminotransferase | 4 |
|  |  | K00811 | aspartate aminotransferase, chloroplastic | 1 |
|  |  | K00815 | tyrosine aminotransferase | 4 |
|  |  | K00817 | histidinol-phosphate aminotransferase | 1 |
|  |  | K07253 | phenylpyruvate tautomerase | 1 |
|  |  | K00430 | peroxidase | 83 |
|  |  | K00487 | trans-cinnamate 4-monooxygenase | 5 |
|  |  | K00588 | caffeoyl-CoA O-methyltransferase | 5 |
|  |  | K01904 | 4-coumarate--CoA ligase | 12 |
|  |  | K10775 | phenylalanine ammonia-lyase | 7 |

Supplementary table 4 K-means cluster for DEGs

| subcluster1 | | | | | |
| --- | --- | --- | --- | --- | --- |
| geneID | AC | AM | AF | Gene Length | Swissprot Description |
| c46935_g1 | 0 | 0.085 | -1.11 | 2488 | Feruloyl CoA ortho-hydroxylase 1 OS=Arabidopsis thaliana GN=F6'H1 PE=1 SV=1 |
| c50341_g1 | 0 | 0.304 | -0.8 | 3256 | 3'-N-debenzoyl-2'-deoxytaxol N-benzoyltransferase OS=Taxus canadensis GN=TAX10 PE=1 SV=1 |
| c51064_g2 | 0 | 2.667 | -0.66 | 2391 | MATE efflux family protein FRD3 OS=Arabidopsis thaliana GN=FRD3 PE=1 SV=1 |
| c51110_g2 | 0 | 0.783 | -0.45 | 3770 | -- |
| c51528_g1 | 0 | 1.093 | -2.74 | 701 | -- |
| c52316_g5 | 0 | 0.06 | -0.96 | 1548 | Feruloyl CoA ortho-hydroxylase 1 OS=Arabidopsis thaliana GN=F6'H1 PE=1 SV=1 |
| c54944_g3 | 0 | 1.528 | -0.4 | 4745 | -- |
| c55106_g1 | 0 | 0.596 | -2.7 | 1572 | -- |
| c64489_g1 | 0 | 2.268 | -1.45 | 819 | Non-specific lipid-transfer protein 1 OS=Lens culinaris PE=3 SV=1 |
| subcluster2 | | | | | |
| geneID | AC | AM | AF |  |  |
| c22869_g1 | 0 | 0.384 | 1.394 | 1425 | Endochitinase OS=Pisum sativum PE=2 SV=1 |
| c2991_g1 | 0 | 1.2 | 1.5 | 861 | Organ-specific protein S2 OS=Pisum sativum PE=2 SV=1 |
| c31736_g2 | 0 | 0.964 | 1.458 | 657 | 1-aminocyclopropane-1-carboxylate oxidase 5 OS=Arabidopsis thaliana GN=At1g77330 PE=2 SV=1 |
| c35383_g1 | 0 | 0.669 | 1.073 | 609 | 14 kDa proline-rich protein DC2.15 OS=Daucus carota PE=2 SV=1 |
| c35383_g2 | 0 | 0.694 | 1.109 | 386 | -- |
| c35947_g1 | 0 | 0.921 | 1.147 | 588 | -- |
| c37787_g1 | 0 | 0.786 | 1.218 | 993 | Pro-hevein OS=Hevea brasiliensis GN=HEV1 PE=1 SV=2 |
| c37936_g1 | 0 | 1.582 | 2.236 | 1139 | -- |
| c37966_g2 | 0 | 0.708 | 1.047 | 898 | -- |
| c38095_g1 | 0 | 0.365 | 2.648 | 1805 | Glucan endo-1,3-beta-glucosidase OS=Glycine max PE=1 SV=1 |
| c38108_g1 | 0 | 0.45 | 1.168 | 631 | Defensin-like protein OS=Nelumbo nucifera PE=3 SV=1 |
| c38663_g1 | 0 | 0.493 | 0.949 | 1079 | Actin-3 OS=Pisum sativum PE=2 SV=1 |
| c41941_g1 | 0 | 1.01 | 0.632 | 818 | -- |
| c42704_g2 | 0 | 0.317 | 1.001 | 990 | Lectin OS=Leucomphalos mildbraedii PE=1 SV=1 |
| c45822_g1 | 0 | 0.671 | 1.243 | 1468 | Peroxidase 4 OS=Vitis vinifera GN=GSVIVT00023967001 PE=1 SV=1 |
| c47696_g1 | 0 | 1.081 | 0.265 | 1857 | High affinity nitrate transporter 2.4 OS=Arabidopsis thaliana GN=NRT2.4 PE=2 SV=1 |
| c48207_g1 | 0 | -0.23 | 0.884 | 1721 | Basic 7S globulin 2 OS=Glycine max PE=1 SV=1 |
| c49130_g1 | 0 | 1.186 | 0.891 | 2053 | Vacuolar-processing enzyme OS=Phaseolus vulgaris PE=1 SV=1 |
| c49605_g1 | 0 | 0.992 | 0.063 | 2199 | Protein NRT1/ PTR FAMILY 6.3 OS=Arabidopsis thaliana GN=NPF6.3 PE=1 SV=1 |
| c50194_g2 | 0 | 0.769 | 1.049 | 2097 | Aspartic proteinase A1 OS=Arabidopsis thaliana GN=APA1 PE=1 SV=1 |
| c50287_g1 | 0 | -0.67 | 0.645 | 2266 | 21 kDa seed protein OS=Theobroma cacao GN=ASP PE=2 SV=1 |
| c50764_g1 | 0 | 0.538 | 1.141 | 2327 | Primary amine oxidase OS=Pisum sativum PE=1 SV=1 |
| c50987_g1 | 0 | 0.726 | 1.037 | 2537 | Probable galactinol--sucrose galactosyltransferase 1 OS=Arabidopsis thaliana GN=RFS1 PE=2 SV=1 |
| c51167_g1 | 0 | 1.287 | 0.387 | 1651 | Siroheme synthase OS=Pseudomonas aeruginosa (strain PA7) GN=cysG PE=3 SV=1 |
| c51322_g1 | 0 | 0.803 | 1.061 | 2218 | 36.4 kDa proline-rich protein OS=Solanum lycopersicum GN=TPRP-F1 PE=2 SV=1 |
| c51408_g1 | 0 | 0.639 | 1.06 | 1737 | -- |
| c51529_g1 | 0 | 1.382 | 0.625 | 2324 | Probable nucleoredoxin 1 OS=Arabidopsis thaliana GN=At1g60420 PE=1 SV=1 |
| c52036_g1 | 0 | 0.613 | 1 | 2650 | D-3-phosphoglycerate dehydrogenase 2, chloroplastic OS=Arabidopsis thaliana GN=PGDH2 PE=1 SV=2 |
| c53696_g1 | 0 | 0.732 | 1.533 | 2627 | -- |
| c64400_g1 | 0 | 0.817 | 1.069 | 1495 | Probable polygalacturonase At3g15720 OS=Arabidopsis thaliana GN=At3g15720 PE=1 SV=1 |
| c73121_g1 | 0 | 0.497 | 1.002 | 393 | 14 kDa proline-rich protein DC2.15 OS=Daucus carota PE=2 SV=1 |
| c73315_g1 | 0 | 0.119 | 0.953 | 2256 | Asparagine synthetase [glutamine-hydrolyzing] 2 OS=Lotus japonicus GN=AS2 PE=2 SV=2 |
| subcluster3 | | | | | |
| geneID | AC | AM | AF |  |  |
| c24873_g1 | 0 | -1.06 | -0.8 | 2172 | Patatin-like protein 2 OS=Oryza sativa subsp. japonica GN=PLP2 PE=3 SV=1 |
| c31006_g1 | 0 | -1.22 | -0.47 | 1732 | -- |
| c32730_g1 | 0 | -1.34 | -1.02 | 669 | Calcium-binding protein PBP1 OS=Arabidopsis thaliana GN=PBP1 PE=1 SV=1 |
| c33880_g1 | 0 | -1.62 | -0.86 | 1409 | -- |
| c35450_g1 | 0 | -1.5 | -0.69 | 987 | -- |
| c36764_g1 | 0 | -1.21 | -0.91 | 843 | -- |
| c36884_g1 | 0 | -1.35 | -0.91 | 1075 | -- |
| c38244_g1 | 0 | -1.07 | -0.79 | 1301 | -- |
| c39522_g1 | 0 | -1.57 | -0.79 | 2200 | Syntaxin-121 OS=Arabidopsis thaliana GN=SYP121 PE=1 SV=1 |
| c39887_g1 | 0 | -1.23 | -0.93 | 1471 | -- |
| c40125_g2 | 0 | -1.2 | -0.56 | 945 | -- |
| c40371_g1 | 0 | -1.24 | -0.76 | 1259 | Ethylene-responsive transcription factor 4 OS=Nicotiana sylvestris GN=ERF4 PE=2 SV=1 |
| c41312_g1 | 0 | -1.17 | -0.58 | 872 | -- |
| c41482_g1 | 0 | -2.08 | -0.58 | 1329 | -- |
| c42402_g1 | 0 | -0.96 | -1.07 | 959 | Kunitz-type trypsin inhibitor-like 2 protein OS=Pisum sativum GN=PIP20-2 PE=1 SV=1 |
| c42690_g1 | 0 | -1.7 | -0.48 | 2274 | -- |
| c42820_g1 | 0 | -1.08 | -0.8 | 1890 | Protein EXORDIUM OS=Arabidopsis thaliana GN=EXO PE=2 SV=1 |
| c43107_g1 | 0 | -1.17 | -0.61 | 1674 | F-box protein At2g27310 OS=Arabidopsis thaliana GN=At2g27310 PE=2 SV=1 |
| c43849_g1 | 0 | -1.15 | -0.62 | 1865 | -- |
| c43993_g1 | 0 | -1.48 | -0.62 | 1541 | Zinc finger protein ZAT10 OS=Arabidopsis thaliana GN=ZAT10 PE=2 SV=1 |
| c44189_g1 | 0 | -1.23 | -0.43 | 1657 | Zinc finger protein ZAT10 OS=Arabidopsis thaliana GN=ZAT10 PE=2 SV=1 |
| c44598_g1 | 0 | -1.36 | -1.02 | 1070 | Probable WRKY transcription factor 2 OS=Arabidopsis thaliana GN=WRKY2 PE=2 SV=1 |
| c44855_g1 | 0 | -1.09 | -0.12 | 1918 | U-box domain-containing protein 20 OS=Arabidopsis thaliana GN=PUB20 PE=2 SV=1 |
| c44917_g1 | 0 | -1.33 | -0.98 | 2168 | BTB/POZ domain-containing protein At5g41330 OS=Arabidopsis thaliana GN=At5g41330 PE=2 SV=1 |
| c45567_g1 | 0 | -1.29 | -0.87 | 1377 | -- |
| c46184_g1 | 0 | -0.57 | -1.45 | 2540 | Protein LHY OS=Arabidopsis thaliana GN=LHY PE=1 SV=2 |
| c46303_g1 | 0 | -1 | -1.32 | 1385 | Zinc transporter 5 OS=Oryza sativa subsp. japonica GN=ZIP5 PE=2 SV=1 |
| c46324_g1 | 0 | -1.19 | -1.04 | 2012 | Oxysterol-binding protein-related protein 3A OS=Arabidopsis thaliana GN=ORP3A PE=1 SV=1 |
| c46800_g1 | 0 | -1.08 | -0.84 | 3505 | TGACG-sequence-specific DNA-binding protein TGA-1B (Fragment) OS=Nicotiana tabacum GN=TGA1B PE=2 SV=1 |
| c46844_g2 | 0 | -1.11 | -0.89 | 1959 | Protein DEHYDRATION-INDUCED 19 homolog 3 OS=Arabidopsis thaliana GN=DI19-3 PE=1 SV=1 |
| c46930_g1 | 0 | -1.2 | -1.05 | 2184 | Protein kinase 2B, chloroplastic OS=Arabidopsis thaliana GN=APK2B PE=2 SV=1 |
| c47012_g1 | 0 | -1.51 | -0.51 | 2093 | Probable receptor-like protein kinase At5g20050 OS=Arabidopsis thaliana GN=At5g20050 PE=2 SV=1 |
| c47117_g1 | 0 | -1.64 | -1.01 | 1426 | -- |
| c47536_g2 | 0 | -1.46 | -0.66 | 2234 | E3 ubiquitin-protein ligase ATL6 OS=Arabidopsis thaliana GN=ATL6 PE=1 SV=2 |
| c47560_g1 | 0 | -1.25 | -0.7 | 1871 | Protein YLS9 OS=Arabidopsis thaliana GN=YLS9 PE=2 SV=1 |
| c47627_g2 | 0 | -1.64 | -0.58 | 977 | Putative calcium-binding protein CML19 OS=Oryza sativa subsp. japonica GN=CML19 PE=3 SV=1 |
| c47901_g1 | 0 | -1.23 | -0.63 | 3738 | Scarecrow-like protein 13 OS=Arabidopsis thaliana GN=SCL13 PE=2 SV=2 |
| c48060_g1 | 0 | -1.51 | -0.68 | 2736 | COBRA-like protein 7 OS=Arabidopsis thaliana GN=COBL7 PE=1 SV=2 |
| c48190_g1 | 0 | -1.22 | -0.91 | 1683 | Arogenate dehydratase/prephenate dehydratase 6, chloroplastic OS=Arabidopsis thaliana GN=ADT6 PE=1 SV=1 |
| c48208_g2 | 0 | -1.5 | -0.5 | 2044 | Putative nuclease HARBI1 OS=Rattus norvegicus GN=Harbi1 PE=2 SV=1 |
| c48506_g1 | 0 | -1.07 | -0.6 | 3750 | Zinc finger CCCH domain-containing protein 29 OS=Arabidopsis thaliana GN=At2g40140 PE=2 SV=1 |
| c48651_g1 | 0 | -1.53 | -1.05 | 1968 | -- |
| c48657_g1 | 0 | -1.19 | -0.62 | 1771 | -- |
| c49002_g1 | 0 | -1.15 | -1.1 | 3889 | Transcription factor RF2b OS=Oryza sativa subsp. japonica GN=RF2b PE=1 SV=1 |
| c49163_g1 | 0 | -1.61 | -0.49 | 2408 | L-type lectin-domain containing receptor kinase IX.1 OS=Arabidopsis thaliana GN=LECRK91 PE=2 SV=1 |
| c49228_g1 | 0 | -1.26 | -0.91 | 1804 | Nudix hydrolase 17, mitochondrial OS=Arabidopsis thaliana GN=NUDT17 PE=2 SV=1 |
| c49675_g1 | 0 | -1.46 | -0.2 | 2540 | U-box domain-containing protein 19 OS=Arabidopsis thaliana GN=PUB19 PE=2 SV=1 |
| c50672_g1 | 0 | -1.16 | -1.01 | 2840 | Probable WRKY transcription factor 33 OS=Arabidopsis thaliana GN=WRKY33 PE=1 SV=2 |
| c50828_g1 | 0 | -0.66 | -1.16 | 3182 | Potassium channel SKOR OS=Arabidopsis thaliana GN=SKOR PE=1 SV=1 |
| c51648_g1 | 0 | -1.07 | -1.17 | 4282 | Calcium-transporting ATPase 12, plasma membrane-type OS=Arabidopsis thaliana GN=ACA12 PE=2 SV=1 |
| c51654_g1 | 0 | -1.75 | -0.65 | 2169 | Nematode resistance protein-like HSPRO2 OS=Arabidopsis thaliana GN=HSPRO2 PE=1 SV=1 |
| c51946_g1 | 0 | -1.25 | -0.93 | 2650 | Protein NRT1/ PTR FAMILY 7.3 OS=Arabidopsis thaliana GN=NPF7.3 PE=1 SV=2 |
| c52526_g1 | 0 | -1.19 | -1.08 | 2823 | Dual specificity protein kinase splA OS=Dictyostelium discoideum GN=splA PE=1 SV=3 |
| c52929_g1 | 0 | -1.36 | -0.9 | 3501 | Ras GTPase-activating protein 4 OS=Homo sapiens GN=RASA4 PE=2 SV=2 |
| c53732_g1 | 0 | -0.89 | -1.08 | 3928 | Glycosyltransferase family protein 64 protein C5 OS=Arabidopsis thaliana GN=At5g04500 PE=2 SV=1 |
| c53783_g1 | 0 | -1.36 | -1.14 | 3220 | Probable serine/threonine-protein kinase At1g18390 OS=Arabidopsis thaliana GN=At1g18390 PE=2 SV=2 |
| c54131_g1 | 0 | -1.02 | -0.94 | 4367 | Probable disease resistance protein At5g66900 OS=Arabidopsis thaliana GN=At5g66900 PE=3 SV=1 |
| c54300_g1 | 0 | -1.31 | -1.17 | 1111 | -- |
| c54377_g1 | 0 | -1.37 | -0.76 | 3411 | Receptor-like protein kinase FERONIA OS=Arabidopsis thaliana GN=FER PE=1 SV=1 |
| c54642_g4 | 0 | -1.01 | -0.61 | 3403 | Transcription factor bHLH13 OS=Arabidopsis thaliana GN=BHLH13 PE=2 SV=1 |
| c54741_g2 | 0 | -0.85 | -0.82 | 6448 | Dynamin-related protein 4C OS=Arabidopsis thaliana GN=DRP4C PE=2 SV=1 |
| c54866_g1 | 0 | -1.71 | -0.78 | 3619 | Receptor-like protein 12 OS=Arabidopsis thaliana GN=RLP12 PE=2 SV=2 |
| c55146_g2 | 0 | -1.78 | -0.71 | 2946 | U-box domain-containing protein 21 OS=Arabidopsis thaliana GN=PUB21 PE=2 SV=1 |
| c55830_g1 | 0 | -1.32 | -1.11 | 1714 | Ethylene-responsive transcription factor 1A OS=Arabidopsis thaliana GN=ERF1A PE=1 SV=2 |
| c68816_g1 | 0 | -1.6 | -0.93 | 2082 | Transcription factor MYC4 OS=Arabidopsis thaliana GN=MYC4 PE=1 SV=1 |
| subcluster4 | | | | | |
| geneID | AC | AM | AF |  |  |
| c45030_g1 | 0 | 3.859 | 1.379 | 1623 | WAT1-related protein At1g70260 OS=Arabidopsis thaliana GN=At1g70260 PE=2 SV=1 |
| c46942_g1 | 0 | 4.47 | 2.32 | 1333 | Lectin OS=Leucomphalos mildbraedii PE=1 SV=1 |
| c52585_g1 | 0 | 2.26 | 1.18 | 3464 | Inducible nitrate reductase [NADH] 2 OS=Glycine max GN=INR2 PE=2 SV=1 |
| c7242_g1 | 0 | 3.248 | 0.868 | 1891 | -- |
| subcluster5 | | | | | |
| geneID | AC | AM | AF |  |  |
| c35294_g1 | 0 | -3.42 | -3.39 | 658 | -- |
| c38925_g1 | 0 | -5.77 | -3.73 | 1570 | Dehydration-responsive element-binding protein 1C OS=Arabidopsis thaliana GN=DREB1C PE=2 SV=2 |
| c41694_g1 | 0 | -4.24 | -3.52 | 2142 | Tryptophan synthase beta chain 2, chloroplastic OS=Camptotheca acuminata GN=TSB PE=2 SV=1 |
| c43589_g1 | 0 | -3.83 | -2.02 | 1156 | Ethylene-responsive transcription factor ERF109 OS=Arabidopsis thaliana GN=ERF109 PE=1 SV=1 |
| c44072_g1 | 0 | -3.29 | -2.48 | 2139 | Myb-related protein Myb4 OS=Oryza sativa subsp. japonica GN=MYB4 PE=2 SV=2 |
| c44588_g1 | 0 | -4.08 | -3.03 | 1344 | Dehydration-responsive element-binding protein 1C OS=Arabidopsis thaliana GN=DREB1C PE=2 SV=2 |
| c47588_g1 | 0 | -3.23 | -2.78 | 1473 | F-box protein PP2-B15 OS=Arabidopsis thaliana GN=PP2B15 PE=2 SV=2 |
| c47813_g3 | 0 | -4.05 | -3.31 | 1694 | -- |
| c48161_g1 | 0 | -4.94 | -3.18 | 1219 | Dehydration-responsive element-binding protein 1F OS=Arabidopsis thaliana GN=DREB1F PE=2 SV=1 |
| c51411_g1 | 0 | -3.38 | -2.48 | 2358 | Abscisic acid 8'-hydroxylase 1 OS=Arabidopsis thaliana GN=CYP707A1 PE=2 SV=1 |
| c51727_g1 | 0 | -2.77 | -3.04 | 1980 | -- |
| c51904_g1 | 0 | -3.8 | -3.3 | 2382 | Transcription factor bHLH25 OS=Arabidopsis thaliana GN=BHLH25 PE=2 SV=2 |
| c53320_g1 | 0 | -3.25 | -2.68 | 2075 | -- |
| c53770_g4 | 0 | -3.46 | -3.05 | 2584 | Probable serine/threonine-protein kinase Cx32, chloroplastic OS=Arabidopsis thaliana GN=At4g35600 PE=1 SV=2 |
| c54322_g1 | 0 | -2.81 | -3.66 | 2969 | Probable 1-deoxy-D-xylulose-5-phosphate synthase 2, chloroplastic OS=Oryza sativa subsp. japonica GN=Os07g0190000 PE=2 SV=1 |
| subcluster6 | | | | | |
| geneID | AC | AM | AF |  |  |
| c20251_g1 | 0 | -1.83 | -1.18 | 3792 | Disease resistance protein RPM1 OS=Arabidopsis thaliana GN=RPM1 PE=1 SV=1 |
| c31769_g1 | 0 | -2.35 | -0.93 | 663 | Putative calcium-binding protein CML19 OS=Oryza sativa subsp. japonica GN=CML19 PE=3 SV=1 |
| c36413_g1 | 0 | -1.96 | -1.51 | 1475 | Protein LURP-one-related 15 OS=Arabidopsis thaliana GN=At5g01750 PE=1 SV=1 |
| c37110_g1 | 0 | -1.67 | -1.31 | 1461 | -- |
| c38184_g1 | 0 | -2.14 | -1.28 | 889 | -- |
| c39067_g2 | 0 | -1.71 | -1.58 | 3828 | TMV resistance protein N OS=Nicotiana glutinosa GN=N PE=1 SV=1 |
| c39301_g1 | 0 | -1.6 | -1.2 | 1433 | Protein TIFY 11B OS=Arabidopsis thaliana GN=TIFY11B PE=1 SV=1 |
| c41115_g1 | 0 | -1.56 | -1.11 | 1852 | Probable protein phosphatase 2C 63 OS=Arabidopsis thaliana GN=At4g33920 PE=2 SV=1 |
| c42857_g1 | 0 | -1.45 | -1.43 | 2436 | -- |
| c45099_g1 | 0 | -1.68 | -1.13 | 1234 | Protein EXORDIUM OS=Arabidopsis thaliana GN=EXO PE=2 SV=1 |
| c46110_g1 | 0 | -0.38 | -1.95 | 1400 | Nicotianamine synthase OS=Solanum lycopersicum GN=CHLN PE=2 SV=1 |
| c47431_g1 | 0 | -1.58 | -1.22 | 2362 | -- |
| c47801_g1 | 0 | -1.69 | -1.46 | 2027 | -- |
| c48114_g1 | 0 | -1.25 | -1.39 | 3315 | Probable serine/threonine-protein kinase At1g54610 OS=Arabidopsis thaliana GN=At1g54610 PE=1 SV=1 |
| c48137_g1 | 0 | -1.93 | -1.63 | 2377 | Probable WRKY transcription factor 40 OS=Arabidopsis thaliana GN=WRKY40 PE=1 SV=1 |
| c48509_g1 | 0 | -1.72 | -1.36 | 2572 | CBL-interacting serine/threonine-protein kinase 12 OS=Arabidopsis thaliana GN=CIPK12 PE=1 SV=1 |
| c48512_g2 | 0 | -1.53 | -1.66 | 2231 | L-ascorbate oxidase homolog OS=Nicotiana tabacum PE=1 SV=1 |
| c48568_g2 | 0 | -1.79 | -1.52 | 2173 | Protein TIFY 10A OS=Arabidopsis thaliana GN=TIFY10A PE=1 SV=1 |
| c48617_g1 | 0 | -2.05 | -1.31 | 3787 | Probable WRKY transcription factor 41 OS=Arabidopsis thaliana GN=WRKY41 PE=2 SV=2 |
| c48779_g1 | 0 | -1.84 | -1.47 | 3054 | Lipid phosphate phosphatase delta OS=Arabidopsis thaliana GN=LPPD PE=2 SV=1 |
| c49193_g1 | 0 | -1.69 | -1.3 | 2372 | Ferredoxin-thioredoxin reductase catalytic chain, chloroplastic OS=Glycine max GN=FTRC PE=1 SV=1 |
| c49406_g1 | 0 | -1.62 | -1.19 | 1919 | CBS domain-containing protein CBSX5 OS=Arabidopsis thaliana GN=CBSX5 PE=2 SV=2 |
| c49468_g1 | 0 | -1.9 | -1.48 | 2273 | -- |
| c49475_g1 | 0 | -1.85 | -1.07 | 2276 | Probable protein phosphatase 2C 25 OS=Arabidopsis thaliana GN=At2g30020 PE=1 SV=1 |
| c49482_g1 | 0 | -1.4 | -1.34 | 2143 | Ammonium transporter 2 OS=Arabidopsis thaliana GN=AMT2 PE=1 SV=2 |
| c50006_g1 | 0 | -1.72 | -1.73 | 2181 | -- |
| c50433_g1 | 0 | -1.72 | -1.41 | 1549 | Probable WRKY transcription factor 33 OS=Arabidopsis thaliana GN=WRKY33 PE=1 SV=2 |
| c50598_g4 | 0 | -1.57 | -1.33 | 1973 | Omega-3 fatty acid desaturase, chloroplastic OS=Glycine max GN=FAD7 PE=2 SV=1 |
| c50650_g1 | 0 | -1.76 | -1.39 | 2589 | -- |
| c51373_g1 | 0 | -1.81 | -1.12 | 1818 | Putative UDP-glucose flavonoid 3-O-glucosyltransferase 3 OS=Fragaria ananassa GN=GT3 PE=2 SV=1 |
| c51871_g1 | 0 | -1.65 | -1.79 | 2705 | Calcium-dependent protein kinase 28 OS=Arabidopsis thaliana GN=CPK28 PE=1 SV=1 |
| c51918_g1 | 0 | -1.38 | -1.57 | 3726 | Testis-expressed sequence 2 protein OS=Mus musculus GN=Tex2 PE=1 SV=2 |
| c52241_g1 | 0 | -1.89 | -1.14 | 2289 | Protein EXORDIUM OS=Arabidopsis thaliana GN=EXO PE=2 SV=1 |
| c52340_g3 | 0 | -1.77 | -1.13 | 1461 | -- |
| c53173_g2 | 0 | -1.29 | -1.44 | 2199 | Aspartic proteinase-like protein 1 OS=Arabidopsis thaliana GN=At5g10080 PE=1 SV=1 |
| c53468_g1 | 0 | -1.23 | -1.61 | 2048 | Aspartic proteinase Asp1 OS=Oryza sativa subsp. japonica GN=ASP1 PE=2 SV=1 |
| c53477_g2 | 0 | -1.98 | -1.73 | 2500 | NAC domain-containing protein 86 OS=Arabidopsis thaliana GN=NAC086 PE=2 SV=1 |
| c53694_g1 | 0 | -1.44 | -1.35 | 2374 | Mitogen-activated protein kinase kinase kinase YODA OS=Arabidopsis thaliana GN=YDA PE=1 SV=1 |
| c53730_g1 | 0 | -1.62 | -1.16 | 5237 | Uncharacterized protein At2g39910 OS=Arabidopsis thaliana GN=At2g39910 PE=2 SV=2 |
| c53851_g2 | 0 | -1.49 | -1.5 | 3988 | Extra-large guanine nucleotide-binding protein 1 OS=Arabidopsis thaliana GN=XLG1 PE=1 SV=2 |
| c54366_g1 | 0 | -1.34 | -1.69 | 7867 | Calcium-transporting ATPase 2, plasma membrane-type OS=Arabidopsis thaliana GN=ACA2 PE=1 SV=1 |
| c54927_g1 | 0 | -1.4 | -1.58 | 2891 | WD repeat-containing protein 82 OS=Danio rerio GN=wdr82 PE=2 SV=1 |
| c55039_g1 | 0 | -1.49 | -1.76 | 2626 | TMV resistance protein N OS=Nicotiana glutinosa GN=N PE=1 SV=1 |
| c55163_g3 | 0 | -1.04 | -1.64 | 2413 | Probable ADP-ribosylation factor GTPase-activating protein AGD11 OS=Arabidopsis thaliana GN=AGD11 PE=2 SV=1 |
| c55270_g1 | 0 | -1.79 | -1.07 | 2699 | -- |
| c55432_g1 | 0 | -1.55 | -1.68 | 2317 | TMV resistance protein N OS=Nicotiana glutinosa GN=N PE=1 SV=1 |
| c55525_g2 | 0 | -1.59 | -2.17 | 2189 | Putative disease resistance protein At4g11170 OS=Arabidopsis thaliana GN=At4g11170 PE=2 SV=1 |
| c55625_g1 | 0 | -1.37 | -1.2 | 5575 | TMV resistance protein N OS=Nicotiana glutinosa GN=N PE=1 SV=1 |
| c55690_g1 | 0 | -1.66 | -1.26 | 4574 | Putative disease resistance protein At4g11170 OS=Arabidopsis thaliana GN=At4g11170 PE=2 SV=1 |
| subcluster7 | | | | | |
| geneID | AC | AM | AF |  |  |
| c10189_g1 | 0 | -2.23 | -1.73 | 719 | Protein TIFY 10B OS=Arabidopsis thaliana GN=TIFY10B PE=1 SV=1 |
| c20023_g1 | 0 | -2.14 | -1.83 | 4077 | Cellulose synthase-like protein D3 OS=Arabidopsis thaliana GN=CSLD3 PE=1 SV=1 |
| c38757_g1 | 0 | -2.53 | -2.32 | 1051 | Calcium-binding protein PBP1 OS=Arabidopsis thaliana GN=PBP1 PE=1 SV=1 |
| c38929_g1 | 0 | -2.6 | -2.21 | 964 | Protein TIFY 5A OS=Arabidopsis thaliana GN=TIFY5A PE=1 SV=1 |
| c40732_g1 | 0 | -2.06 | -2.07 | 2273 | -- |
| c41525_g1 | 0 | -3.23 | -1.85 | 977 | -- |
| c44622_g1 | 0 | -3.1 | -2.01 | 890 | -- |
| c45439_g2 | 0 | -3.13 | -2.13 | 1254 | Probable ADP-ribosylation factor GTPase-activating protein AGD13 OS=Arabidopsis thaliana GN=AGD13 PE=2 SV=1 |
| c45734_g1 | 0 | -2.69 | -1.87 | 1243 | Protein TIFY 5A OS=Arabidopsis thaliana GN=TIFY5A PE=1 SV=1 |
| c45769_g1 | 0 | -2.33 | -1.9 | 1979 | Vacuolar amino acid transporter 1 OS=Saccharomyces cerevisiae (strain ATCC 204508 / S288c) GN=AVT1 PE=1 SV=1 |
| c48395_g1 | 0 | -2.11 | -1.61 | 2507 | StAR-related lipid transfer protein 7, mitochondrial OS=Mus musculus GN=Stard7 PE=2 SV=2 |
| c48568_g1 | 0 | -2.35 | -2.08 | 1627 | Protein TIFY 10A OS=Arabidopsis thaliana GN=TIFY10A PE=1 SV=1 |
| c48910_g1 | 0 | -2.23 | -1.87 | 3070 | F-box protein PP2-B1 OS=Arabidopsis thaliana GN=PP2B1 PE=1 SV=1 |
| c49953_g1 | 0 | -2.32 | -1.66 | 2604 | MACPF domain-containing protein NSL1 OS=Arabidopsis thaliana GN=NSL1 PE=2 SV=1 |
| c49960_g1 | 0 | -2.33 | -1.39 | 2320 | Chitin-inducible gibberellin-responsive protein 1 OS=Oryza sativa subsp. japonica GN=CIGR1 PE=2 SV=1 |
| c50126_g1 | 0 | -2.07 | -1.75 | 1311 | Probable xyloglucan endotransglucosylase/hydrolase protein 23 OS=Arabidopsis thaliana GN=XTH23 PE=2 SV=1 |
| c50236_g1 | 0 | -2.62 | -2.02 | 2291 | Lipase-like PAD4 OS=Arabidopsis thaliana GN=PAD4 PE=1 SV=1 |
| c50377_g1 | 0 | -2.37 | -1.8 | 4533 | Protein FAR1-RELATED SEQUENCE 5 OS=Arabidopsis thaliana GN=FRS5 PE=2 SV=1 |
| c50878_g1 | 0 | -2.72 | -1.52 | 2132 | Serine/threonine-protein phosphatase 6 regulatory ankyrin repeat subunit B OS=Homo sapiens GN=ANKRD44 PE=1 SV=3 |
| c51526_g1 | 0 | -2.44 | -1.7 | 2770 | -- |
| c52142_g2 | 0 | -2.7 | -2.01 | 3092 | Scarecrow-like protein 5 OS=Arabidopsis thaliana GN=SCL5 PE=2 SV=1 |
| c52264_g5 | 0 | -2.17 | -2.72 | 2362 | Probable disease resistance protein At5g66900 OS=Arabidopsis thaliana GN=At5g66900 PE=3 SV=1 |
| c52332_g1 | 0 | -2.85 | -2 | 3437 | Putative calcium-transporting ATPase 13, plasma membrane-type OS=Arabidopsis thaliana GN=ACA13 PE=3 SV=1 |
| c52535_g1 | 0 | -2.14 | -2.3 | 3722 | Phosphoinositide phospholipase C 6 OS=Arabidopsis thaliana GN=PLC6 PE=2 SV=2 |
| c53792_g1 | 0 | -2.89 | -1.55 | 3412 | Calcium-transporting ATPase 12, plasma membrane-type OS=Arabidopsis thaliana GN=ACA12 PE=2 SV=1 |
| c54458_g1 | 0 | -2.19 | -2.29 | 5976 | F-box protein At1g78280 OS=Arabidopsis thaliana GN=At1g78280 PE=2 SV=3 |
| c5985_g1 | 0 | -2.16 | -1.75 | 1920 | Transcription factor bHLH25 OS=Arabidopsis thaliana GN=BHLH25 PE=2 SV=2 |
| c68944_g1 | 0 | -2.55 | -1.48 | 972 | Zinc finger protein ZAT11 OS=Arabidopsis thaliana GN=ZAT11 PE=2 SV=1 |
| subcluster8 | | | | | |
| geneID | AC | AM | AF |  |  |
| c54146_g5 | 0 | 19.83 | 19.87 | 2822 | -- |
